# Antiproliferative and anti-inflammatory polyhydroxylated spirostanol saponins from *Tupistra chinensis*

**DOI:** 10.1038/srep31633

**Published:** 2016-08-17

**Authors:** Limin Xiang, Xiaomin Yi, Yihai Wang, Xiangjiu He

**Affiliations:** 1 School of Pharmacy, Guangdong Pharmaceutical University, Guangzhou 510006, China

## Abstract

*Tupistra chinensis* is widely distributed in southwestern China and its rhizome is a famous folk medicine for the treatment of carbuncles and pharyngitis. Its chemical identity of potent antiproliferative and anti-inflammatory constituents has been carried out in this study. Twenty-three polyhydroxylated spirostanol saponins, including nine novels, were isolated and identified. The new spirostanol saponins were elucidated as spirost-25(27)-en-1*β*,2*β*,3*β*,4*β*,5*β*-pentol-2-*O*-*β*-D-xylopyranoside (**1**), spirost-25(27)- en-1*β*,2*β*,3*β*,4*β*,5*β*-pentol-2-*O*-*α-*L-arabinopyranoside (**2**), spirost-25(27)-en- 1*β*,3α,5*β*-triol (**12**), spirost-25(27)-en-1*β*,3α,4*β*,5*β*,6*β*-pentol (**13**), spirost-25(27)-en- 1*β*,2*β*,3*β*,5*β*-tetraol-5-*O*-*β*-D-glucopyranoside (**16**), 5*β*-spirost-25(27)-en-1*β*,3*β*-diol- 3-*O*-*β*-D-glucopyranosyl-(1 → 4)-*β*-D-glucopyranoside (**17**), (25*R*)-5*β*-spirostan- 1*β*,3*β*-diol-3-*O*-*β*-D-glucopyranosyl-(1 → 6)-*β*-D-glucopyranoside (**18**), (25*R*)-5*β*- spirostan-1*β*,3*β*-diol-3-*O*-*β*-D-fructofuranosyl-(2 → 6)-*β*-D-glucopyranoside (**19**), 5*β*-spirost-25(27)-en-3*β*-ol-3-*O*-*β*-D-glucopyranosyl-(1 → 4)-*β*-D-glucopyranoside (**20**). The antiproliferative effects against seven human cancer cell lines and inhibitory activities on nitric oxide (NO) production induced by lipopolysaccharide (LPS) in a macrophage cell line RAW 264.7 were assayed for all the isolated compounds. Compounds **17**, **19** and **21** exhibited potential antiproliferative activities against all of human cancer cell lines tested. Compounds **21** showed significant inhibition on NO production with IC_50_ values of 11.5 μM. These results showed that the spirostanol saponins isolated from the dried rhizomes of *T. chinensis* have potent antiproliferative and anti-inflammatory activities and *T. chinensis* might be used as anticancer and.anti-inflammatory supplement.

Medicinal plants have been used for treating human diseases and they produced a large variety of secondary metabolites such as alkaloids, flavonoids, triterpenoids and steroidal glycosides^1^. Steroidal glycosides as the important secondary metabolites of medicinal plants have been reported to possess a wide range of biological activities including anticancer, anti-inflammatory, platelet aggregation inhibition, antihypertensive, cholesterol lowering, antifungal and antiviral^2^. Additionally, steroidal glycosides have a wide variety of commercial uses such as surfactants, foaming agents and precursors for the industrial production of pharmaceutical drugs^3^,^4^.

A series of widespread chronic diseases such as cancer are now the leading causes of morbidity and mortality worldwide. Many cancers arise from sites of infection, chronic irritation and inflammation. Recent data have expanded the concept that inflammation is a critical component of tumor progression. Anti-inflammatory therapy is efficacious towards early neoplastic progression and malignant conversion^5^,^6^. Many studies have showed that biological activities of phytochemicals are comparable to previous synthetic compounds, and they have low side effects. Therefore, recent interests in medicinal plants have been focused on identifying active compounds and elucidating underlying molecular mechanisms of action^7^,^8^.

The genus *Tupistra* (Liliaceae) has 12 species in southern China. These species possess similar morphologic characteristics and some can be substituted for each other as a folk medicine to treat pharyngolaryngitis, rheumatic diseases and snake-bite^9^. *Tupistra chinensis* is widely distributed in southwestern China and its dried rhizome is a famous folk medicine for the treatment of carbuncles and pharyngitis. Previous phytochemical investigations on *T. chinensis* have led to the isolation of a variety of biologically active compounds, including steroidal sapogenins and their glycosides^10^,^11^,^12^,^13^,^14^,^15^, cardenolides^16^, a pregnane genin and its glycoside^17^,^18^, flavonoids^17^,^18^,^19^, which have anti-inflammatory^20^, cytotoxicity^21^,^22^,^23^ and antifungal activities^24^,^25^. Steroidal saponins that consist of spirostanol saponins and furostanol saponins were the most abundant active constituents in *T. chinensis*.

In the course of our continuing search for bioactive leading compounds from the medicinal herbs, we focused on steroidal saponins from the dried rhizomes of *T. chinensis* and obtained twenty-three polyhydroxylated spirostanol saponins including nine new compounds. The structures of all the isolated compounds were elucidated on the basis of spectroscopic data and chemical methods, including IR, NMR, MS, and GC analysis. Moreover, all of the isolated compounds were evaluated for their antiproliferative activity against seven human cancer cell lines and the inhibitory activities on NO production induced by LPS in a macrophage cell line RAW 264.7.

## Results and Discussion

### Structure identification

**Figure 1:**
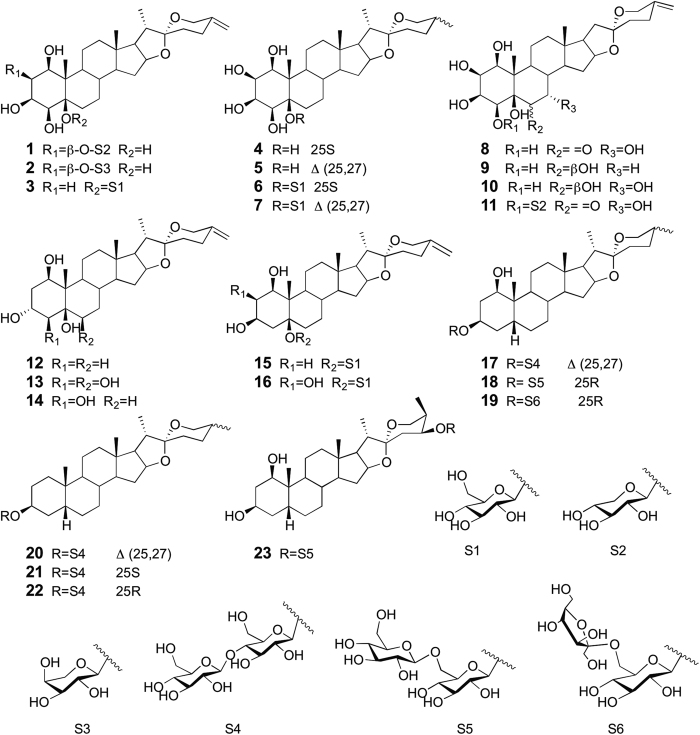
Structures of isolated compounds **1**–**23**

Nine new spirostanol saponins (**1**, **2**, **12**, **13**, **16**–**20**) and fourteen congeners (**3**–**11**, **14**, **15**, **21**–**23**) were obtained from the 60% ethanol extract of the rhizomes of *T. chinensis*. Their chemical structures are shown in Fig. 1. The structures of all the isolated compounds were elucidated on the basis of spectroscopic data and chemical methods, including IR, NMR, MS, and GC analysis. The fourteen known spirostanols were identified by comparison of their reported spectroscopic data as spirost-25(27)-en-1*β*,3*β*,4*β*,5*β*-tetrol-5-*O*-*β*-D-glucopyranoside (**3**)^23^, neopentrogenin (**4**)^26^, Δ^25(27)^-pentrogenin (**5**)^12^, neopentrogenin-5-O-*β*-D- glucopyranoside (**6**)^26^, spirost-25(27)-en-1*β*,2*β*,3*β*,4*β*,5*β*-pentol-5-*O*-*β*-D- glucopyranoside (**7**)^26^, spirost-25(27)-en-1*β*,2*β*,3*β*,4*β*,5*β*,7*α*-hexaol-6-one (**8**)^15^, wattigenin A (**9**)^27^, spirost-25(27)-en-1*β*,2*β*,3*β*,4*β*,5*β*,6*β*,7*α*-heptol (**10**)^28^, spirost-25(27)-en-1*β*,2*β*,3*β*,4*β*,5*β*,7*α*-hexaol-6-one-4-*O*-*β*-D-xylopyranoside (**11**)^29^, tupichigenin D (**14**)^18^, spirost-25(27)-en-1*β*,3*β*,5*β*-triol-5-*O*-*β*-D-glucopyranoside (**15**)^23^, (25*S*)-5*β*-spirostan-3*β*-ol-3-*O*-*β*-D-glucopyranosyl-(1 → 4)-*β*-D- glucopyranoside (**21**)^30^, (25*R*)-5*β*-spirostan-3*β*-ol-3-*O*-*β*-D-glucopyranosyl-(1 → 4)- *β*-D-glucopyranoside (**22**)^30^, wattoside I (**23**)^9^. Compounds **4**, **6**, **7**, **21** and **22** are reported for the first time from *T. chinensis*, to our knowledge. The NMR data of the known compounds could be found in the supplementary material.

**Figure 2:**
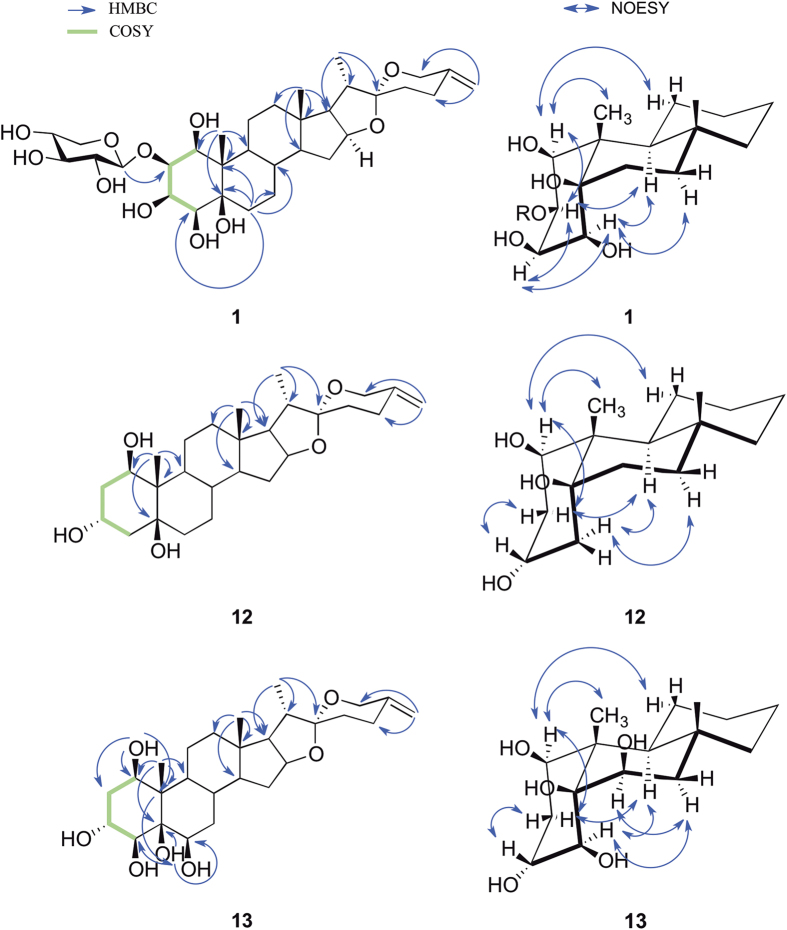
Selected ^1^H − ^1^H COSY, HMBC and NOESY correlations of compounds **1**, **12** and **13**.

Compound **1** was isolated as white amorphous powder, 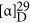 −43.3 (*c* 0.25, MeOH). The molecular formula was inferred as C_32_H_50_O_11_ according to the positive-ion HRESI-MS peak at *m*/*z* [M + H]^+^ 611.3437 (calcd for C_32_H_51_O_11_ [M + H]^+^, 611.3431). The IR spectrum showed a strong absorption band at 3364 cm^−1^, ascribable to hydroxyl functionalities. Acid hydrolysis and GC analysis of **1** gave D-xylose. The ^1^H NMR spectrum showed three typical steroidal methyl signals at *δ*_H_ 1.59 (3H, s, Me-19), 1.09 (3H, d, *J* = 6.9 Hz, Me-21) and 0.84 (3H, s, Me-18) (Table 1). Four methane protons indicative of secondary alcoholic functions at *δ*_H_ 4.57 (1H, brs, H-1), 4.41 (1H, m, H-2), 5.03 (1H, brs, H-3) and 4.31 (1H, brs, H-4) were observed. The germinal protons at C-27 were observed at *δ*_H_ 4.57 and 4.82 as two singlets, which are characteristic of an exocyclic methylene. The ^13^C NMR spectrum exhibited characteristic spirostanol carbon signal at *δ*_C_ 109.8 (C-22) and three methyl (*δ*_C_ 16.8, 13.9, and 15.3), and two olefinic carbons *δ*_C_ 144.7 and 109.1 were assigned to the C-25 and C-27 positions, respectively, diagnostic of C-25 (27)-unsaturated sapogenin. The NMR spectrum of **1** showed an anomeric proton signal at *δ*_H_ 5.15 (1H, d, 7.5 Hz), corresponding to an anomeric carbon at *δ*_C_ 103.5 in the HSQC spectrum, indicated the presence of one sugar moiety. Unambiguous complete assignments for the ^1^H and ^13^C NMR signals were made by combination of DEPT, ^1^H − ^1^H COSY, HSQC, HMBC, and NOESY spectra. The ^1^H − ^1^H COSY spectrum showed that the oxymethine proton *δ*_H_ 4.41 (H-2) was coupled to *δ*_H_ 4.57 (1H, brs, H-1) and 5.03 (1H, brs, H-3), and the oxymethine proton *δ*_H_ 5.03 (H-3) was coupled to the oxymethine proton at *δ*_H_ 4.31 (H-4). These findings supported the location of the hydroxyl groups at C-1, C-2, C-3, C-4, and C-5, together with the long-range correlations observed in the HMBC spectrum (Fig. 2). The HMBC correlations of H-1 (*δ*_H_ 5.15) of xylose with C-2 (*δ*_C_ 74.4) of the aglycon revealed the sugar residue attached to C-2 of the aglycon. The stereochemistry of the ring junctions and substituents of the aglycon of **1** were determined via cross-peaks observed in a NOESY spectrum according to the refs 12,15 and 23 (Fig. 2). The key correlations between H-4_α_ and H-7_α_/H-9_α_, and between H-2_α_/H-9_α_, supported the A/B *cis* ring junction pattern. Thus, the hydroxyl groups at C-5 and C-2 have a *β*-orientation. Additional NOE correlations between H-1_α_ and Me-19/H-11, and between H-3_α_ and H-2_α_/H-4_α_, were indicative of *β*-orientations for OH-1, OH-3 and OH-4. Thus, compound **1** was elucidated as spirost-25(27)-en-1*β*,2*β*,3*β*,4*β*,5*β*-pentol-2-*O*-*β*-D-xylopyranoside.

Compound **2** was isolated as white amorphous powder, with the same molecular formula of C_32_H_50_O_11_ as **1** on the basis of HRESIMS (*m*/*z* 611.3412 [M + H]^+^). Acid hydrolysis of **2** gave L-arabinose. From a comparison of ^1^H and ^13^C NMR data of **2** with those of **1** (Table 1), it was apparent that **2** contained the same aglycon as **1**, except for a little different in the monosaccharide chain. Instead of the signals for a xylopyranosyl moiety, signals assignable to an α-L-arabinopyranosyl residue were observed at *δ*_H_ 5.13 (1H, d, *J* = 7.0 Hz, H-1), 4.56 (1H, m, H-2), 4.22 (1H, m, H-3), 4.36 (1H, m, H-4), 4.38 (1H, m, H-5a) and 3.85 (1H, m, H-5b), and *δ*_C_ 103.4 (C-1), 72.8 (C-2), 74.8 (C-3),69.7 (C-4), 67.3 (C-5). The linkage position of the arabinosyl moiety to C-2 of the aglycone was confirmed by an HMBC correlation between the anomeric proton at *δ*_H_ 5.13 and the C-2 carbon of the aglycone at *δ*_C_ 74.0. Therefore, the structure of **2** was elucidated as spirost-25(27)-en-1*β*,2*β*,3*β*,4*β*,5*β*-pentol- 2-*O*-*α*-L-arabinopyranoside.

Compound **12** was isolated as white amorphous powder, 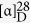 −33.1 (*c* 0.25, MeOH). The HRESIMS gave an ion at *m*/*z* 447.3112 [M + H]^+^ (calcd for C_27_H_43_O_5_, 447.3110), consistent with the molecular formula C_27_H_42_O_5_. The ^1^H NMR spectrum showed three typical steroidal methyl signals at *δ*_H_ 1.50 (3H, s, Me-19), 1.12 (3H, d, *J* = 7.0 Hz, Me-21) and 0.87 (3H, s, Me-18) (Table 2). The ^13^C NMR spectrum exhibited characteristic spirostanol carbon signal at *δ*_C_ 109.8 (C-22) and three methyl (*δ*_C_ 16.9, 13.7, and 15.4), and two olefinic carbons *δ*_C_ 144.8 and 109.1 were observed, diagnostic of C-25 (27)-unsaturated sapogenin. The ^1^H − ^1^H COSY spectrum showed that two methylene protons at *δ* 2.12 (H-2_α_) and 2.61 (H-2_β_) were coupled to *δ* 4.31 (H-1) and 5.13 (H-3), and the oxymethine proton *δ* 5.13 (H-3) was coupled to two methylene protons at *δ* 2.26 (H-4_β_) and 2.61 (H-4_α_). These findings indicated the location of the hydroxyl groups at C-1, C-3, and C-5, together with the long-range correlations observed in the HMBC spectrum (Fig. 2). The key NOESY correlations (Fig. 2) between H-4_α_ and H-7_α_/H-9_α_, and between H-2_α_ and H-9_α_, supported the A/B *cis* ring junction pattern. Thus, the hydroxyl group at C-5 has a *β*-orientation. Additional NOE correlations between H-1_α_ and Me-19/H-11, and between H-3_β_ and H-2_β_/H-4_β_, were indicative of *β*-orientation for OH-1 and *α*-orientation for OH-3. On the basis of the above analysis, compound **12** was finally assigned to be spirost-25(27)-en-1*β*,3*α*,5*β*-triol.

Compound **13** was isolated as white amorphous powder, 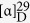 −34.9 (*c* 0.25, MeOH). The molecular formula, C_27_H_42_O_7_, was deduced from the HRESI-MS peak (*m*/*z* [M + H]^+^ 479.3018, calcd for C_27_H_43_O_7_, 479.3009). The ^1^H NMR spectrum showed three typical steroidal methyl signals at *δ*_H_ 1.86 (3H, s, Me-19), 1.10 (3H, d, *J* = 7.0 Hz, Me-21) and 0.87 (3H, s, Me-18) (Table 2). The down-field shifted hydrogen chemical shift of Me-19 in combination with an oxymethine proton (*δ*_H_ 4.94) at B ring confirmed the presence of the OH group attached at C-6 in compound 13, which was consistent with previous reports^12^. The ^13^C NMR spectrum exhibited characteristic spirostanol carbon signal at *δ*_C_ 109.8 (C-22) and three methyl (*δ*_C_ 16.9, 16.9, and 15.4), and two olefinic carbons *δ*_C_ 144.8 and 109.1 were observed, diagnostic of C-25 (27)-unsaturated sapogenin. These ^1^H-NMR data and ^13^C-NMR signals suggested that **13** was a C-25(27) unsaturated spirostane type steroidal sapogenin. The ^1^H-^1^H COSY spectrum showed that two methylene protons at *δ* 2.23 (H-2_α_) and 2.62 (H-2_β_) were coupled to *δ* 4.19 (H-1) and 4.85 (H-3), and the oxymethine proton *δ* 4.34 (H-4) was in turn coupled with the oxygenated methane proton at *δ* 4.85 (H-3). The oxygenated methane proton at *δ* 4.94 (H-6) was coupled with two methylene protons at *δ* 1.55 (H-7_α_) and 2.07 (H-7_β_). These findings supported the location of the hydroxyl groups at C-1, C-3, C-4, and C-6. The key NOESY correlations (Fig. 2) between H-4_α_ and H-7_α_/H-9_α_, and between H-2_α_ and H-9_α_, supported the A/B *cis* ring junction pattern. Thus, the hydroxyl groups at C-4 and C-5 have *β*-orientation. Additional NOE correlations between H-1_α_ and Me-19/H-11, and between H-3_β_ and H-2_β_, between H-6_α_ and H-7_α_, were indicative of *β*-orientation for OH-1, OH-6 and *α*-orientation for OH-3. On the basis of the above analysis, compound **13** was formulated as spirost-25(27)-en-1*β*,3*α*,4*β*,5*β*,6*β*-pentol.

Compound **16** was isolated as white amorphous powder. The molecular formula of C_33_H_52_O_11_ was determined by the positive-ion HRESIMS peak at *m*/*z* [M + H]^+^ 625.3552 (calcd for C_33_H_53_O_11_, 625.3588). The ^1^H NMR spectrum showed three typical steroidal methyl signals at *δ*_H_ 1.62 (3H, s, Me-19), 1.08 (3H, d, *J* = 7.0 Hz, Me-21) and 0.83 (3H, s, Me-18) (Table 3). The ^1^H NMR spectrum of **16** was similar to that of **1**, except for the absence of one oxymethine proton. This suggests that both compounds possessed the same C-25(27) unsaturated spirostane type skeleton. The ^13^C NMR spectrum exhibited characteristic spirostanol carbon signal at *δ*_C_ 109.8 (C-22) and three methyl (*δ*_C_ 16.8, 14.1, and 15.3), and two olefinic carbons *δ*_C_ 144.8 and 109.1 were assigned to the C-25 and C-27 positions, respectively. Compared the NMR data with those of spirost-25(27)-en-1*β*,2*β*,3*β*,5*β*-tetraol-5-*O*-*β*-D-galactopyranoside^31^, the NMR features of those two compounds were almost same except for the signals of the sugar moiety. The NMR spectrum of **16** showed an anomeric proton signal at *δ*_H_ 5.29 (1H, d, *J* = 7.7 Hz, H-1-Glc), corresponding to an anomeric carbon at *δ*_C_ 97.7 in the HSQC spectrum, indicated the presence of one sugar moiety. The sugar was identified as D-glucose by GC analysis of its chiral derivatives after acid hydrolysis. Thus, compound **16** was inferred as spirost-25(27)-en-1*β*,2*β*,3*β*,5*β*-tetraol-5-*O*-*β*-D- glucopyranoside.

Compound **17** was isolated as white amorphous powder. The HRESIMS of **17** showed the ion at m/z 755.4275 [M + H]^+^, and its molecular formula was inferred as C_39_H_62_O_14_ on the basis of the analysis of ^1^H and ^13^C NMR and DEPT spectra. Acid hydrolysis and GC analysis of **17** gave D-glucose. Compared the ^1^H and ^13^C NMR data with those of isorhodeasapogenin 3-*O*-*β*-D-glucopyranosyl-(1 → 4)-*β*-D- glucopyranoside^32^, the NMR features of those two compounds were almost same except for the signals of the F-ring, which was suggested the same A-E ring substitution in the two compounds. The signals due to the methyl of C-27 in isorhodeasapogenin 3-*O*-*β*-D-glucopyranosyl-(1 → 4)-*β*-D-glucopyranoside were replaced by the signals assigned to the C-25(27) exo-methylene group at *δ*_H_ 4.79 (1H, s) and 4.82(1H, s), and *δ*_C_ 144.8 (C-25) and 109.1 (C-27) in **17**. Hence, the structure of **17** was elucidated as 5*β*-Spirost-25(27)-en-1*β*,3*β*-diol-3-*O*-*β*-D-glucopyranosyl- (1 → 4)-*β*-D-glucopyranoside.

Compound **18** was obtained as white amorphous powder. The molecular formula of C_39_H_64_O_14_ was assigned from the positive-ion HRESIMS peak (*m*/*z* 757.4403 [M + H]^+^, calcd for C_39_H_65_O_14_ [M + H]^+^, 757.4374). The IR spectrum showed the characteristic absorption of hydroxyl group at 3411 cm^−1^ and the four steroidal sapogenins absorption bands 986, 919, 899, 873 cm^−1^, and the 899 band was stronger than the 919 band. It was suggested that **18** was a sapogenin with “iso” configuration of the F ring^33^. Acid hydrolysis and GC analysis of **18** gave D-glucose. From a comparison of ^1^H and ^13^C NMR data of **18** (Table 4) with those of isorhodeasapogenin 3-*O*-glucopyranosyl-(1 → 4)-*β*-D-glucopyranoside^32^, it was apparent that **18** contained the same aglycon as isorhodeasapogenin 3-*O*-glucopyranosyl-(1 → 4)-β-D- glucopyranoside, except for a little different in the saccharide chains. The linkage of the sugar units was established from the following HMBC correlations: H-1 (*δ*_H_ 5.02) of the additional glucose′ with C-6 (*δ*_C_ 70.7) of the glucose, H-1 (*δ*_H_ 4.93) of the glucose with C-3 (*δ*_C_ 76.0) of the aglycon. Thus, compound **18** was elucidated as (25*R*)-5*β*-spirostan-1*β*,3*β*-diol-3-*O*-*β*-D-glucopyranosyl-(1 → 6)-*β*-D-glucopyranoside.

Compound **19** was isolated as white amorphous powder. The HRESIMS of **19** showed the ion at *m*/*z* 757.4401 [M + H]^+^, and its molecular formula was inferred as C_39_H_64_O_14_ on the basis of the analysis of ^1^H and ^13^C NMR and DEPT spectra. On acid hydrolysis, **19** liberated D-fructose and D-glucose, identified by GC analysis of their chiral derivatives. From comparison of the NMR data of **19** with those of **18**, it was indicated that **19** contained the same aglycone as **18**. The HMBC correlations between the H-6 (*δ*_H_ 4.85, 4.55) of the glucose and C-2 (*δ*_C_ 106.2) of the fructose indicated the saccharide attached to C-3 of the aglycone was *β*-D-fructofuranosyl-(2 → 6)-*β*-D- glucopyranosyl. Thus, compound **19** was elucidated as (25*R*)-5*β*-spirostan-1*β*,3*β*-diol- 3-*O*-*β*-D-fructofuranosyl-(2 → 6)-*β*-D-glucopyranoside.

Compound **20** was isolated as white amorphous powder. The HRESIMS of **20** showed the ion at *m*/*z* 739.4254 [M + H]^+^, and its molecular formula was inferred as C_39_H_62_O_13_ on the basis of the analysis of ^1^H and ^13^C NMR and DEPT spectra. Compared the ^1^H and ^13^C NMR data with those of (25*R*)-5*β*-spirostan-3*β*-ol-3-*O*-*β*-D- glucopyranosyl-(1 → 4)-*β*-D-glucopyranoside^34^, the NMR features of those two compounds were almost same except for the signals of the F-ring, which was suggested the same A-E ring substitution in the two compounds. The signals due to the methyl of C-27 in (25*R*)-5*β*-spirostan-3*β*-ol-3-*O*-*β*-D-glucopyranosyl-(1 → 4)-*β*-D- glucopyranoside were replaced by the signals assigned to the C-25(27) exo-methylene group at *δ*_H_ 4.78 (1H, s) and 4.82(1H, s), and *δ*_C_ 144.8 (C-25) and 109.1 (C-27) in **20**. Hence, the structure of **20** was elucidated as 5*β*-Spirost-25(27)-en-3*β*-ol-3-*O*-*β*-D- glucopyranosyl-(1 → 4)-*β*-D-glucopyranoside.

### Antiproliferative activities

TB, TC, TD and all the isolated compounds were evaluated for antiproliferative activities against seven human tumor cell lines including FaDu (human hypopharyngeal carcinoma), Detroit 562 (human metastatic pharyngeal squamous cell carcinoma), CNE-1 (high differentiation human nasopharyngeal carcinoma), CNE-2 (low differentiation human nasopharyngeal carcinoma), HepG2 (human hepatocellular carcinoma), K562 (human chronic leukemia), SPC-A-1 (human lung adenocarcinoma) with a modified MTT method according to reported protocols, and cis-dichlorodiammineplatinum (II) was used as a positive control. The results were shown in Table 5 and Table S2 for the isolated compound and the crude extract of *T. chinensis*, respectively. TD showed moderate activity against five out of seven cancer cell lines (Fadu, Detroit 562, HepG2, K562 and SPC-A-1) with IC_50_ values of 20.9–29.3 μg/mL. TC exhibited selective antiproliferative activity against Fadu, Detroit 562, and K562 with IC_50_ values of 6.7 ± 0.5, 45.9 ± 1.7, and 6.5 ± 2.3 μg/mL, respectively. Compounds **17**, **19** and **21** exhibited moderate antiproliferative effects against all of human cancer cell lines tested. Compound **22** showed moderate inhibitory activity against six out of seven human cancer cell lines tested excepted CNE-2. Compound **18** displayed weak inhibitory activities against Detroit 562, CNE-2 and HepG2 with IC_50_ values of 39.4 ± 0.7, 40.7 ± 3.1 and 46.5 ± 1.0 μM, respectively. Compound **20** showed moderate antiproliferative activity against FaDu, Detroit 562 and HepG2. Compound **4** exhibited moderate inhibitory activity against FaDu and HepG2 with IC_50_ values of 27.6 ± 3.6 and 29.7 ± 3.1 μM, respectively. Compounds **3**, **5** and **12** exhibited potential antiproliferative activities against FaDu with IC_50_ values of 28.6 ± 1.6, 41.2 ± 1.8 and 12.1 ± 1.2 μM, respectively. The other compounds exhibited IC_50_ values larger than 50.0 μM against the seven tested cell lines and were considered to be inactive. Compounds **17**–**22** showed higher antiproliferative activities against human tumor cell lines than other compounds, suggesting that glycosylation at C-3 of the aglycon play an important role in the growth inhibition on cancer cells of spirostanol saponins. The structure-activity relationship of the isolated compounds (**1**–**23**) indicated that the glycosylation at C-3 of the aglycon was the most important structural feature for the high antiproliferative activity and other structural features (e.g., OH position and number) played a modified role in enhancing or reducing the activity. However comparison of antiproliferative activity is only an approximation as there are notable differences in IC_50_ values between control compounds. A choice of reference and cell lines may lead to various conclusions with respect to the potency of antiproliferative compounds^35^.

### Anti-inflammatory activities

Nitric oxide (NO) is a signaling molecule that plays a key role in the pathogenesis of inflammation^36^. NO production by immune cells has been used as an indicator of the presence and extent of inflammation as well as the effectiveness of anti-inflammatory agents^37^.

Compounds **1**–**23** were tested for their inhibitory effects on NO production induced by LPS in a macrophage cell line RAW 264.7. Cell viability was first determined by the MTT method to find whether inhibition of NO production was due to the cytotoxicity of the tested samples. The anti-inflammatory activities were summarized in Table 6. All the tested samples exhibited no cytotoxicity against RAW 264.7 macrophage cells at their effective concentrations. The crude extracts of *T. chinensis* TB, TC and TD showed moderate inhibition on NO production with IC_50_ values of 32.6 ± 3.7, 37.5 ± 5.4 and 36.6 ± 1.8 μg/mL (see Table S3 in the supporting information). Compound **21** showed significant inhibition on NO production with IC_50_ values of 11.5 μM, comparable to that of the positive control indomethacin.at 47.4 μM^38^. Compounds **1**, **4**, **9**, **10**, **13**, **14**, **17** and **20** exhibited moderate inhibition with IC_50_ values between 21.7 and 33.6 μM. Compounds **5**, **7**, **15**, **18** and **19** displayed inhibition activity, but quite limited with IC_50_ values of 45.9 ± 3.1, 45.2 ± 5.7, 46.1 ± 13.1, 41.6 ± 13.5 and 45.4 ± 11.2 μM, respectively. Other compounds showed weak activity with IC_50_ value above than 50 μM. The structure-activity relationship of the isolated spirostanol saponins shows that small changes such as different positions or the number of the hydroxyl groups, *R*/*S* configuration on the aglycone led to changes in activity. The most active compound is **21**, and comparison with **22** shows that change in the orientation or of the methyl group at C-25 from axial in **21** to equatorial in **22** diminishes the activity^39^. Comparison of the activity of **4** and **5** shows that **4** is more active than **5** due to the exocyclic double in **5**, which can be illustrated by the relative higher IC_50_ value of compound **20** and the relative lower IC_50_ value of compound **21**. The kinds, the number and the linkage of sugar also play important roles in their anti-inflammatory activity^40^.

## Conclusion

Twenty-three polyhydroxylated spirostanol saponins, including nine new compounds (**1**, **2**, **12**, **13**, **16**–**20**) were isolated from the rhizomes of *T. chinensis* and their structures were determined by spectroscopic methods. Among the isolated compounds, Compounds **17**, **19** and **21** exhibited potential antiproliferative activities against all of human cancer cell lines tested. Compound **21** showed significant inhibition on NO production with IC_50_ values of 11.5 μM. The present investigation suggested that *T. chinensis* can be a potential source of natural antiproliferative and anti-inflammatory agents and its spirostanol saponins may be responsible for the biological activity. However, further studies are required, not only to assess the bioactivities of the isolates *in vivo*, but also to investigate the mechanisms underlying the observed biological activities of isolated compounds.

## Materials and Methods

### Chemicals

HPLC-grade methanol was purchased from Oceanpak Chemical Co. (Gothenburg, Sweden). D101 macroporus resin (Xi′an Lanxiao Resin Corporation Ltd., Xi′an, China), silica gel (200–300 mesh, Anhui Liangchen Silicon Material Co. Ltd., Lu’an, China) and ODS (40–60 μm, Merck KGaA, Darmstadt, Germany) were used for column chromatography. All solvents used for column chromatography were of analytical grade and purchased from Sinopharm Chemical Reagent Co. (Shanghai, China). Foetal bovine serum (FBS) and Dulbecco’s modified Eagle’s medium (DMEM) were purchased from HyClone Laboratories (Logan, UT, USA). Sugar reagents used for GC analysis, 3-(4,5-dimethylthiazol-2-yl)-2,5-diphenyltetrazolium bromide (MTT), cis-dichlorodiammineplatinum (II) and indomethacin were purchased from Sigma Chemical Co. (St. Louis, MO, USA).

### General experimental procedures

Optical rotations were measured with a JASCO P-1020 digital polarimeter. IR spectra were recorded on a PerkinElmer 100 IR spectrometer with KBr. NMR spectra were obtained on a Bruker Ultrashield 500 Plus instrument, using tetramethylsilane as internal standard. Waters AQUITY UPLC/Q-TOF mass spectrometer was used to record HRESIMS spectra. Semi-preparative HPLC was performed using a RAININ pump equipped with a Gilson 133 refractive index detector. A semi-preparative column (COSMOSIL-Pack 5C_18_-MS-II, 10ID × 250 mm, 5 μm) was used for HPLC. Microplate reader (Kehua Technologies, Inc., Shanghai, China) was used for bioassay.

### Plant material

The rhizomes of *T. chinensis* Baker was purchased from Shennongjia Forest District (Shennongjia, China), and identified by Prof. Xiangjiu He, the School of Pharmacy, Guangdong Pharmaceutical University. A voucher specimen (No. GDPU-NPR-2013002) was deposited in the Department of Medicinal Chemistry, Guangdong Pharmaceutical University, Guangzhou, China.

### Extraction and isolation

The air-dried rhizomes of *T. chinensis* (17.0 kg) were extracted four times with 60% EtOH (each 70 L × 4 h) under reflux. The combined EtOH extracts were evaporated under reduced pressure (for complete removal of organic solvent) to yield an aqueous suspension (20 L), and then partitioned between H_2_O (20 L) and EtOAc (20 L ×  4) to give an EtOAc fraction (218.0 g). The aqueous fraction was applied to a D101 macroporous resin column, eluted with H_2_O (50 L), 20% EtOH (50 L), 60% EtOH (50 L) and 80% EtOH (50 L), repectively. The solvents were evaporated under vacuum to yield 20% EtOH eluted fraction (TB, 194.5 g) and 60% EtOH eluted fraction (TC, 458 g) and 80% EtOH eluted fraction (TD, 83.8 g).

A half part of fraction TC (200.0 g) was subjected to silica gel column chromatography (200 ~ 300 mesh, 3 kg, 95 × 1140 mm) eluted with a gradient of CHCl_3_/MeOH (20:1 to 2:1, followed by MeOH) to yield 18 fractions (C1-C18). Fraction C3 (108.2 mg) was further purified by ODS and semi-preparative HPLC (3.0 mL/min, 85% MeOH in H_2_O isocratic elution) to afford compound **12** (2.7 mg). Fraction C4 (157.5 mg) was separated by an ODS column eluted with a gradient of MeOH-H_2_O (3:7 to 5:5, v/v) to afford compound **14** (23.0 mg). Fraction C5 (625.8 mg) was separated by an ODS column eluted with a gradient of MeOH-H_2_O (1:9 to 2:8, v/v) to afford compound **13** (20.9 mg). Fraction C12 (5.1 g) was subjected to ODS eluted with a gradient of MeOH-H_2_O (3:7 to 8:2, v/v) to afford eight fractions (C12-1 to C12-8). Subfraction C-12-3 (335.5 mg) was further purified by semi-preparative HPLC (3.0 mL/min, 60% MeOH in H_2_O isocratic elution) to afford compound **7** (194.7 mg).

Fraction TD (83.3 g) was separated by silica gel column chromatography (200 ~ 300 mesh, 2 kg, 95 × 745 mm) eluted with a gradient of CHCl_3_/MeOH (100:1 to 1:1, v/v) to obtain twenty-one fractions (D1-D21). Fraction D13 (15.8 g) was separated by an ODS column eluted with a gradient of MeOH-H_2_O (1:9 to 7:3, v/v) to afford nine fractions (D13-1 to D13-9). Compound **5** (15.3 mg) was crystallized from subfraction D13-4 (90.2 mg) in MeOH. Fraction D14 (21.5 g) was subjected to ODS eluted with a gradient of MeOH-H_2_O (1:9 to 8:2, v/v) to afford nine fractions (D14-1 to D14-9). Compound **8** (18.6 mg) was crystallized from subfraction D14-5 in MeOH. Subfraction D-14-7 (335.5 mg) was further purified by semi-preparative HPLC (3.0 mL/min, 70% MeOH in H_2_O isocratic elution) to afford compound **4** (14.3 mg) and compound **15** (32.4 mg). Fraction D15 (1.8 g) was purified by ODS to obtain eleven fractions (D15-1 to D15-11). Subfraction D15-6 (87.4 mg) was purified by semi-preparative HPLC (3.0 mL/min, 80% MeOH in H_2_O isocratic elution) to yield compound **3** (51.8 mg). Fraction D16 (1.5 g) was purified by ODS to obtain three fractions (D16-1 to D16-3). Subfraction D16-3 (1.18 g) was purified by semi-preparative HPLC (3.0 mL/min, 75% MeOH in H_2_O isocratic elution) to yield compounds **20** (17.2 mg), **21** (14.2 mg) and **22** (23.7 mg). Fraction D18 (11.4 g) was subjected to ODS to obtain seventeen fractions (D18-1 to D18-17). Subfraction D18-9 (1.3 g) was further purified by semi-preparative HPLC (3.0 mL/min, 60% MeOH in H_2_O isocratic elution) to yield compounds **9** (87.3 mg). Subfraction D18-10 (649.5 mg) was further purified by semi-preparative HPLC (3.0 mL/min, 60% MeOH in H_2_O isocratic elution) to yield compounds **6** (55.3 mg) and **16** (25.5 mg). Subfraction D18-14 (243.8 mg) was further purified by semi-preparative HPLC (3.0 mL/min, 75% MeOH in H_2_O isocratic elution) to yield compounds **17** (27.7 mg), **18** (22.0 mg) and **19** (58.1 mg). Fraction D19 (3.6 g) was subjected to silica gel column chromatography (300 ~ 400 mesh, 150 g, 95 × 745 mm) eluted with a gradient of CHCl_3_/MeOH (10:1 to 3:1, v/v) to obtain eight fractions (D19-1 to D19-8). Subfraction D19-7 was further purified by semi-preparative HPLC (3.0 mL/min, 70% MeOH in H_2_O isocratic elution) to yield compounds **1** (45.4 mg) and **23** (10.1 mg). Fraction D20 (4.9 g) was subjected to ODS eluted with a gradient of MeOH-H_2_O (1:9 to 8:2, v/v) to obtain ten fractions (D20-1 to D20-10). Subfraction D20-6 was further purified by semi-preparative HPLC (3.0 mL/min, 70% MeOH in H_2_O isocratic elution) to yield compound **2** (28.1 mg). Fraction D21 (4.6 g) was subjected to ODS eluted with a gradient of MeOH-H_2_O (1:9 to 7:3, v/v) to obtain eleven fractions (D21-1 to D21-11). Subfraction D21-6 (1.2 g) was further purified by semi-preparative HPLC (3.0 mL/min, 60% MeOH in H_2_O isocratic elution) to yield compounds **10** (62.0 mg) and **11** (29.2 mg).

*Spirost-25*(*27*)*-en-1β*,*2β*,*3β*,*4β*,*5β-pentol-2-O-β-D-xylopyranoside (**1***) White amorphous powder; 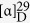 −43.3 (*c* 0.25, MeOH); IR (KBr) *ν*_max_ 3364, 2940, 1645, 1454, 1380, 1045, 920cm^−1^; ^1^H and ^13^C NMR data, see Table 1; HRESIMS *m*/*z* [M + H]^+^ 611.3437 (calcd for C_32_H_51_O_11_, 611.3431).

*Spirost-25*(*27*)*-en-1β*,*2β*,*3β*,*4β*,*5β-pentol-2-O-α-L-arabinopyranoside (**2***) White amorphous powder; 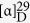 −49.2 (*c* 0.25, MeOH); IR (KBr) *ν*_max_ 3422, 2949, 1650, 1452, 1380, 1047, 923 cm^−1^; ^1^H and ^13^C NMR data, see Table 1; HRESIMS *m*/*z* [M + H]^+^ 611.3412 (calcd for C_32_H_51_O_11_, 611.3431).

*Spirost-25*(*27*)*-en-1β*,*3α*,*5β-triol (**12***) White amorphous powder; 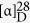 −33.1 (*c* 0.25, MeOH); IR (KBr) *ν*_max_ 3300, 2950, 1650, 1592, 1455, 1381, 1046, 926, 899, 878 cm^−1^; ^1^H and ^13^C NMR data, see Table 2; HRESIMS *m*/*z* [M + H]^+^ 447.3112 (calcd for C_27_H_43_O_5_, 447.3110).

*Spirost-25*(*27*)*-en-1β*,*3α*,*4β*,*5β*,*6β-pentol (**13***) White amorphous powder; 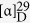 −34.9 (*c* 0.25, MeOH); IR (KBr) *ν*_max_ 3398, 2954, 1717, 1655, 1450, 1381, 1043, 924 cm^−1^; ^1^H and ^13^C NMR data, see Table 2; HRESIMS *m*/*z* [M + H]^+^ 479.3018 (calcd for C_27_H_43_O_7_, 479.3009).

*Spirost-25*(*27*)*-en-1β*,*2β*,*3β*,*5β-tetraol-5-O-β-D-glucopyranoside (**16***) White amorphous powder; 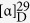 −44.9 (*c* 0.50, MeOH); IR (KBr) *ν*_max_ 3424, 2941, 1651, 1452, 1372, 1046, 922 cm^−1^; ^1^H and ^13^C NMR data, see Table 3; HRESIMS *m*/*z* [M + H]^+^ 625.3552 (calcd for C_33_H_53_O_11_, 625.3588).

*5β-Spirost-25*(*27*)*-en-1β*,*3β-diol-3-O-β-D-glucopyranosyl-*(*1* → *4*)*-β-D-glucopyranoside (**17***) White amorphous powder; 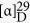 −52.9 (*c* 0.25, MeOH); IR (KBr) *ν*_max_ 3419, 2921, 1645, 1452, 1047, 920 cm^−1^; ^1^H and ^13^C NMR data, see Table 4; HRESIMS *m*/*z* [M + H]^+^ 755.4275 (calcd for C_39_H_63_O_14_, 755.4218).

(*25R*)*-5β-Spirostan-1β*,*3β-diol-3-O-β-D-glucopyranosyl-*(*1* → *6*)*-β-D-glucopyranoside (**18***) White amorphous powder; 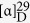 −27.0 (*c* 0.12, MeOH); IR (KBr) *ν*_max_ 3411, 2935, 1641, 1451, 1050, 986, 919, 899, 873 cm^−1^; ^1^H and ^13^C NMR data, see Table 4; HRESIMS *m*/*z* [M + H]^+^ 757.4403 (calcd for C_39_H_65_O_14_, 757.4374).

(*25R*)*-5β-Spirostan-1β*,*3β-diol-3-O-β-D-fructofuranosyl-*(*2* → *6*)*-β-D-glucopyranoside (**19***) White amorphous powder; 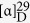 −66.8 (*c* 0.50, MeOH); IR (KBr) *ν*_max_ 3391, 2929, 1632, 1451, 1053, 984, 918, 899, 865 cm^−1^; ^1^H and ^13^C NMR data, see Table 4; HRESIMS *m*/*z* [M + H]^+^ 757.4401 (calcd for C_39_H_65_O_14_, 757.4374).

*5β-Spirost-25*(*27*)*-en-3β-ol-3-O-β-D-glucopyranosyl-*(*1* → *4*)*-β-D- glucopyranoside (**20***) White amorphous powder; 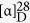 −30.6 (*c* 0.50, MeOH); IR (KBr) *ν*_max_ 3423, 2925, 1634, 1452, 1043, 921, 896 cm^−1^; ^1^H and ^13^C NMR data, see Table 4; HRESIMS *m*/*z* [M + H]^+^ 739.4254 (calcd for C_39_H_63_O_13_, 739.4269).

### Acid hydrolysis and GC analysis

Compounds **1**, **2**, **12**, **13** and **16**–**20** (1–2 mg) were dissolved in 5 mL 2 M HCl and heated at 90 °C for 8 h. The reaction mixture was extracted with EtOAc (5 mL × 3) and the aqueous residue was evaporated under vacuum at 60 °C. Then 5 mg NH_2_OH.HCl was added to the residue and the mixture was dissolved in 600 μL pyridine. After heating at 90 °C for 30 min, 300 μL Ac_2_O was added. After homogenized, the mixture was heated at 90 °C for another 1 h. The reaction mixture was analyzed by GC using standard aldononitrile peracetates as reference samples^41^.

### Measurement of inhibition activity on tumor cell proliferation

Antiproliferative activities against human nasopharyngeal cancer cells (CNE-1, CNE-2, FaDu, Detroit 562), human liver cancer cells (HepG2), human chronic leukemia cells (K562) and human lung adenocarcinoma cells (SPC-A-1) of the pure spirostanol saponins isolated from *T. chinensis* were measured by the modified MTT assay as described previously^42^.

### Measurement of anti-inflammatory activity

Determination of NO production was performed by measuring the accumulation of nitrite in the culture supernatant using the Griess reagent, as previously described^43^.

### Statistical analysis

All the experiments were conducted for three independent replicates, and data were expressed as mean ± SD. Statistical analyses were performed by one-way ANOVA. Dunnett’s Multiple Comparison Test was used to determine the significance of differences between the groups. Differences at P < 0.05 were considered statistically significant.

## Additional Information

**How to cite this article**: Xiang, L. *et al*. Antiproliferative and anti-inflammatory polyhydroxylated spirostanol saponins from *Tupistra chinensis. Sci. Rep.* **6**, 31633; doi: 10.1038/srep31633 (2016).

## Supplementary Material

Supplementary Information

## Figures and Tables

**Table 1:** NMR spectroscopic data for compounds **1** and **2** (pyridine-*d*_*5*_).

Position	1		2	
	*δ*_*C*_	*δ*_H_ (*J* in Hz)	*δ*_*C*_	*δ*_H_ (*J* in Hz)
1	77.8	4.57, brs	77.8	4.58, m
2	74.4	4.41, m	74.0	4.41, m
3	72.8	5.03, brs	72.8	5.03, brs
4	68.7	4.31, brs	68.7	4.31, brs
5	78.2		78.2	
6	30.5	2.48, d (13.2)	30.5	2.48, d (13.2)
		1.67, m		1.67, m
7	28.8	1.54, m	28.8	1.50, m
		1.14, m		1.10, m
8	35.2	1.71, m	35.2	1.71, m
9	45.7	1.22, m	45.7	1.20, m
10	45.5		45.5	
11	21.9	1.58, m	21.9	1.57, m
		1.45, m		1.41, m
12	40.2	1.59, m	40.2	1.58, m
		1.03, m		1.01, m
13	40.9		40.9	
14	56.4	1.03, m	56.4	1.01, m
15	32.5	2.03, m	32.5	2.02, m
		1.44, m		1.43, m
16	81.7	4.59, m	81.8	4.58, m
17	63.3	1.81, m	63.4	1.80, m
18	16.8	0.84, s	16.8	0.83, s
19	13.9	1.59, s	13.9	1.58, s
20	42.2	1.96, m	42.2	1.95, m
21	15.3	1.09, d (7.0)	15.3	1.09, d (7.0)
22	109.8		109.8	
23	33.5	1.81, m	33.5	1.76–1.79, m
24	29.3	2.70, m	29.3	2.70, m
		2.25, d (11.5)		2.24, d (11.5)
25	144.7		144.7	
26	65.4	4.47, d (12.1)	65.4	4.47, d (12.1)
		4.04, d (12.1)		4.04, d (12.1)
27	109.1	4.82, s	109.1	4.81, s
		4.78, s		4.78, s
	2-O-Xyl		2-O-Ara	
1	103.5	5.15, d (7.5)	103.4	5.13, d (7.0)
2	75.4	4.08, m	72.8	4.56, m
3	78.7	4.18, m	74.8	4.22, m
4	71.4	4.24, m	69.7	4.36, m
5	67.7	4.40, m	67.3	4.38, m
		3.76, m		3.85, m

**Table 2:** NMR spectroscopic data for compounds **12** and **13** (pyridine-*d*_*5*_).

Position	12		13	
	*δ*_*C*_	*δ*_H_ (*J* in Hz)	*δ*_*C*_	*δ*_H_ (*J* in Hz)
1	75.9	4.31, brs	76.2	4.19, brs
2	38.9	2.61, m	36.6	2.62, m
		2.12, t (11.1)		2.23, m
3	63.7	5.13, m	69.2	4.85, m
4	44.0	2.61, m	75.9	4.34, d (9.1)
		2.26, m		
5	77.4		80.7	
6	36.7	1.98, m	69.5	4.94, s
		1.58, d (13.2)		
7	29.2	1.49, m	35.4	2.07, m
		1.07, m		1.55, m
8	35.4	1.66, m	30.7	2.38, d (8.4)
9	45.8	1.45, m	45.9	1.50, m
10	43.3		44.9	
11	21.7	1.38, m	21.4	1.50, m
				1.40, m
12	40.3	1.66, m	40.1	1.66, m
		0.99, m		1.00, m
13	41.1		41.1	
14	56.6	0.97, m	56.3	1.02, m
15	32.5	2.05, dd (12.1, 5.5)	32.5	2.07, m
		1.46, m		1.43, m
16	81.8	4.65, dd (14.6, 7.7)	81.8	4.62, dd (14.5, 7.6)
17	63.4	1.85, m	63.4	1.85, m
18	16.9	0.87, s	16.9	0.87, s
19	13.7	1.50, s	16.9	1.86, s
20	42.3	1.98, m	42.2	1.96, m
21	15.4	1.12, d (7.0)	15.4	1.10, d (7.0)
22	109.8		109.8	
23	33.6	1.80, m	33.6	1.77, m
24	29.3	2.73, td (13.2, 5.8)	29.3	2.69, td (13.6, 5.5)
		2.26, m		2.23, m
25	144.8		144.8	
26	65.4	4.51, d (12.0)	65.4	4.47, d (12.1)
		4.06, d (12.0)		4.03, d (11.9)
27	109.1	4.83, s	109.1	4.81, s
		4.80, s		4.78, s

**Table 3:** NMR spectroscopic data for compound **16** (pyridine-*d*_*5*_).

Position	*δ*_*C*_	*δ*_H_ (*J* in Hz)	Position	*δ*_*C*_	*δ*_H_ (*J* in Hz)
1	78.0	4.20, d (7.8)	18	16.8	0.83, s
2	68.4	3.95, m	19	14.1	1.62, s
3	71.4	4.51, brs	20	42.3	1.96, m
4	37.0	2.50, m	21	15.3	1.08, d (7.0)
5	83.4		22	109.8	
6	31.0	2.11, td (13.5)	23	33.6	1.82, m
		2.03, m			1.78, m
7	29.3	1.53, d (13.7)	24	29.3	2.72, td (13.4)
		0.88, dd (12.7, 3.9)			2.25, d (11.5)
8	34.8	1.59, m	25	144.8	
9	45.9	1.17, m	26	65.4	4.48, d (12.2)
10	47.0				4.05, d (12.2)
11	21.8	1.42, m	27	109.1	4.83, s
12	40.2	1.59, m			4.79, s
		1.02, m	5-O-Glc		
13	40.8		1	97.7	5.29, d (7.7)
14	56.3	1.02, m	2	75.9	3.95, m
15	32.5	2.03, m	3	79.1	4.26, t (8.6)
		1.45, m	4	72.0	4.08, m
16	81.8	4.61, m	5	79.1	4.08, m
17	63.3	1.84, m	6	63.1	4.60, m
					4.31, dd (11.5)

**Table 4:** NMR spectroscopic data for compounds **17**–**20** (pyridine-*d*_*5*_).

Position	17		18		19		20	
	*δ*_*C*_	*δ*_H_ (*J* in Hz)	*δ*_*C*_	*δ*_H_ (*J* in Hz)	*δ*_*C*_	*δ*_H_ (*J* in Hz)	*δ*_*C*_	*δ*_H_ (*J* in Hz)
1	72.7	3.89, d (9.1)	72.3	3.95, m	72.3	3.94, brs	31.3	1.70, m
								1.46, m
2	32.3	2.30, m	31.7	2.35, d (11.3)	31.8	2.39, d (14.7)	27.3	1.91, m
		1.84, m		1.82, m		1.80, m		1.52, m
3	75.2	4.53, m	76.0	4.55, m	75.6	4.52, brs	74.9	4.32, m
4	29.5	1.90, m	30.4	1.92, m	30.1	1.89, m	30.9	1.79, m
		1.82, m		1.74, m		1.73, m		1.71, m
5	31.3	2.38, d (11.5)	31.4	2.40, d (14.9)	31.4	2.34, d (12.4)	37.3	2.04, m
6	26.8	1.73, m	26.8	1.72, m	26.8	1.69, m	27.3	1.73, m
		1.15, m		1.10, m		1.10, m		1.06, m
7	26.8	1.28, m	26.6	1.23, m	26.7	1.23, m	27.1	1.24, m
		1.01, m		0.94, m		0.97, m		0.96, m
8	36.1	1.59, m	36.1	1.56, m	36.1	1.56, m	35.9	1.49, m
9	42.5	1.20, m	42.2	1.15, m	42.3	1.15, m	40.6	1.29, m
10	40.8		40.6		40.6		35.6	
11	21.4	1.28, m	21.4	1.23, m	21.4	1.23, m	21.5	1.32, m
								1.19, m
12	40.5	1.66, m	40.5	1.61, m	40.6	1.62, m	40.6	1.68, m
		1.06, m		1.00, m		1.03, m		1.08, m
13	41.0		41.0		41.0		41.3	
14	56.7	1.08, m	56.6	1.05, m	56.6	1.06, m	56.8	1.08, m
15	32.5	2.03, m	32.5	2.02, m	32.5	2.03, m	32.5	2.04, m
		1.43, m		1.42, m		1.14, m		1.42, m
16	81.8	4.60, m	81.5	4.60, m	81.5	4.62, d (7.3)	81.9	4.62, dd (14.7, 7.5)
17	63.5	1.84, m	63.5	1.83, m	63.5	1.86, m	63.5	1.85, m
18	17.0	0.84, s	17.0	0.81, s	17.0	0.81, s	16.9	0.82, s
19	19.5	1.26, s	19.5	1.24, s	19.4	1.23, s	24.2	0.84, s
20	42.2	1.97, m	42.3	1.95, m	42.4	1.96, m	42.2	1.97, m
21	15.4	1.10, d (7.0)	15.4	1.14, d (6.9)	15.4	1.14, d (7.0)	15.4	1.10, d (6.9)
22	109.8		109.6		109.6		109.8	
23	33.6	1.78, m	32.2	1.68, m	32.2	1.68, m	33.6	1.78, m
24	29.3	2.72, m	29.6	1.56, m	29.6	1.56, m	29.3	2.72, td (13.5, 5.4)
		2.26, m						
25	144.8		30.9	1.56, m	31.0	1.56, m	144.8	
26	65.4	4.48, d (12.1)	67.2	3.58, m	67.2	3.60, m	65.4	4.49, m
		4.05, d (12.1)	3.51, t (10.5)	3.51, m		3.51, m		4.05, m
27	109.1	4.82, s	17.7	0.68, d (5.6)	17.7	0.69, d (5.6)	109.1	4.82, s
		4.79, s						4.78, s
	3-O Glc		3-O Glc		3-O Glc		3-O Glc	
1	101.3	4.97, d (7.8)	102.4	4.93, d (7.8)	102.1	4.89, d (7.8)	103.2	4.91, d (7.8)
2	74.9	3.96, m	75.2	3.93, m	75.3	3.91, m	75.1	4.08, m
3	77.2	4.31, m	78.8	4.20, m	78.8	4.20, m	77.3	4.32, m
4	81.5	4.33, m	72.2	4.05, m	72.4	4.07, m	81.7	4.39, t (9.1)
5	77.0	3.96, m	77.3	4.15, m	77.1	4.07, m	76.8	3.92, dt (9.5, 3.2)
6	62.4	4.58, m	70.7	4.89, d (9.1)	63.1	4.85, d (9.1)	62.6	4.60, m
		4.31, m		4.18, m		4.55, m		4.51, m
	Glc(1 → 4)		Glc(1 → 6)		Fru(2 → 6)		Glc(1 → 4)	
1′	105.3	5.21, d (7.9)	105.6	5.02, d (7.7)	63.2	4.37, m	105.3	5.23, d (7.9)
						4.26, d (8.0)		
2′	75.1	4.11, m	75.8	4.06, m	106.2		75.2	4.13, t (8.3)
3′	78.8	4.02, m	78.7	3.93, m	79.8	5.21, m	78.6	4.24, m
4′	71.9	4.22, m	71.8	4.25, m	77.3	5.01, t (7.9)	71.8	4.23, m
5′	78.6	4.22, m	78.8	4.24, m	84.4	4.57, m	78.8	4.01, m
6′	62.8	4.53, m	63.0	4.53, m	64.4	4.37, m	62.7	4.51, m
		4.31, m		4.39, m				4.32, m

**Table 5:** Antiproliferative activities of some compounds from *T. chinensis* against human cancer cell lines.

Compound	IC_50_ (μM)^a^						
	FaDu	Detroit 562	CNE-1	CNE-2	HepG2	K562	SPC-A-1
3	28.6 ± 1.6*	>50.0	>50.0	>50.0	>50.0	>50.0	>50.0
4	27.6 ± 3.6^***^	>50.0	>50.0	>50.0	29.7 ± 3.1^***^	>50.0	>50.0
5	41.2 ± 1.8^***^	>50.0	>50.0	>50.0	>50.0	>50.0	>50.0
12	12.1 ± 1.2	>50.0	>50.0	>50.0	>50.0	>50.0	>50.0
17	20.3 ± 2.1	20.2 ± 2.0	33.6 ± 0.3^***^	23.6 ± 1.9^***^	28.6 ± 1.0^***^	27.4 ± 0.2^***^	24.8 ± 0.7^***^
18	>50.0	39.4 ± 0.7^***^	>50.0	40.7 ± 3.1^***^	46.5 ± 1.0^***^	>50.0	>50.0
19	61.4 ± 2.5^***^	21.5 ± 1.1^***^	50.9 ± 1.1^***^	29.5 ± 0.9^***^	34.0 ± 0.5^***^	40.2 ± 2.1^***^	39.4 ± 1.0^***^
20	20.1 ± 0.4	20.1 ± 0.6^***^	>50.0	>50.0	28.0 ± 0.7^***^	>50.0	>50.0
21	12.7 ± 1.7*	22.7 ± 0.5^**^	17.4 ± 0.5^***^	14.5 ± 1.1^**^	14.3 ± 1.5^***^	22.2 ± 0.2^***^	22.2 ± 0.2^***^
22	20.5 ± 3.4	29.1 ± 0.6*	33.7 ± 0.6^***^	>50.0	17.7 ± 0.3^***^	33.8 ± 0.1^***^	26.3 ± 1.0^***^
CIS^b^	18.1 ± 0.7	26.2 ± 1.5	10.8 ± 0.4	7.9 ± 1.1	6.5 ± 0.3	13.5 ± 0.8	3.8 ± 0.3

^a^Values are presented as means ± SD (*n* = 3).

^b^Positive control.

^*^p < 0.05, **p < 0.01, ***p < 0.001 versus positive control.

**Table 6:** Inhibitory effects of some compounds from *T. chinensis* on NO production induced by LPS in macrophagesa^c^.

Compound	IC_50_ (μM)^a^	Compound	IC_50_ (μM)^a^
**1**	28.7 ± 2.1	**15**	46.1 ± 13.1
**4**	21.7 ± 1.3	**17**	21.0 ± 0.8
**5**	45.9 ± 3.1	**18**	41.6 ± 13.5
**7**	45.2 ± 5.7	**19**	45.4 ± 11.2
**9**	33.6 ± 1.7	**20**	22.6 ± 5.0
**10**	28.7 ± 4.5	**21**	11.5 ± 1.3
**13**	26.1 ± 0.5	**Indomethacin**^b^	47.4 ± 4.5
**14**	29.1 ± 0.6		

^a^Values are presented as means ± SD (*n* = 3).

^b^Positive control.

^c^Compounds **2**, **3**, **6**, **8**, **11**, **12**, **16**, **19**, **22**, **23** with IC_50_ > 50.0 μM.
