# Supplementary material for: Antiproliferative and anti-inflammatory polyhydroxylated spirostanol saponins from Tupistra chinensis
Source: Sci Rep. 2016 Aug 17;6:31633. doi: 10.1038/srep31633 (PMC4987684; doi:10.1038/srep31633)
Supplement: Supplementary Information [file srep31633-s1.doc]

**Antiproliferative and** **anti-inflammatory polyhydroxylated** **spirostanol saponins from *Tupistra chinensis***

Limin Xiang, Xiaomin Yi, Yihai Wang^*^, Xiangjiu He^*^

School of Pharmacy, Guangdong Pharmaceutical University, Guangzhou 510006, China

* To whom correspondence should be addressed:

Dr. Xiangjiu He, Tel/Fax: +86 20 3935 2132. E-mail: hexiangjiu@163.com (X. He)

* Co-correspondence:

Dr. Yihai Wang, Tel: 86-20-3935-2140, E-mail: wangyih88@163.com (Y. Wang)

[**Antiproliferative and anti-inflammatory polyhydroxylated spirostanol saponins from *Tupistra chinensis*** 1](#__RefHeading___Toc455651523)

[**Table S1.** ^13^C NMR data (126 MHz, pyridine-*d_5_*) for the known compounds 5](#__RefHeading___Toc455651524)

[**Table S2.** Antiproliferative activities of the extracts from *T. chinensis* against human cancer cell lines 6](#__RefHeading___Toc455651525)

[**Table S3.** Inhibitory effects of the extracts from *T. chinensis* on NO production induced by LPS in macrophagesa 7](#__RefHeading___Toc455651526)

[**Figure S1.** ^1^H-NMR spectrum (500 MHz, C_5_D_5_N) of compound **1**. 8](#__RefHeading___Toc455651527)

[**Figure S2.** ^13^C-NMR spectrum (126 MHz, C_5_D_5_N) of compound **1**. 8](#__RefHeading___Toc455651528)

[**Figure S3**. HSQC spectrum (500 MHz, C_5_D_5_N) of compound **1**. 8](#__RefHeading___Toc455651529)

[**Figure S4**. HMBC spectrum (500 MHz, C_5_D_5_N) of compound **1**. 9](#__RefHeading___Toc455651530)

[**Figure S5.** ^1^H-^1^H COSY spectrum (500 MHz, C_5_D_5_N) of compound **1**. 9](#__RefHeading___Toc455651531)

[**Figure S6.** NOESY spectrum (500 MHz, C_5_D_5_N) of compound **1.** 10](#__RefHeading___Toc455651532)

[**Figure S7.** HRESIMS spectrum of compound **1.** 10](#__RefHeading___Toc455651533)

[**Figure S8.** IR (KBr disc) spectrum of compound **1.** 11](#__RefHeading___Toc455651534)

[**Figure S9.** ^1^H-NMR spectrum (500 MHz, C_5_D_5_N) of compound **2** 11](#__RefHeading___Toc455651535)

[**Figure S10.** ^13^C-NMR spectrum (126 MHz, C_5_D_5_N) of compound **2** 12](#__RefHeading___Toc455651536)

[**Figure S11**. HSQC spectrum (500 MHz, C_5_D_5_N) of compound **2** 12](#__RefHeading___Toc455651537)

[**Figure S12**. HMBC spectrum (500 MHz, C_5_D_5_N) of compound **2** 13](#__RefHeading___Toc455651538)

[**Figure S13.** ^1^H-^1^H COSY spectrum (500 MHz, C_5_D_5_N) of compound **2** 13](#__RefHeading___Toc455651539)

[**Figure S14.** NOESY spectrum (500 MHz, C_5_D_5_N) of compound **2** 14](#__RefHeading___Toc455651540)

[**Figure S15.** HRESIMS spectrum of compound **2** 14](#__RefHeading___Toc455651541)

[**Figure S16.** IR (KBr disc) spectrum of compound **2** 15](#__RefHeading___Toc455651542)

[**Figure S17.** ^1^H-NMR spectrum (500 MHz, C_5_D_5_N) of compound **12**. 15](#__RefHeading___Toc455651543)

[**Figure S18.** ^13^C-NMR spectrum (126 MHz, C_5_D_5_N) of compound **12**. 16](#__RefHeading___Toc455651544)

[**Figure S19**. HSQC spectrum (500 MHz, C_5_D_5_N) of compound **12**. 16](#__RefHeading___Toc455651545)

[**Figure S20**. HMBC spectrum (500 MHz, C_5_D_5_N) of compound **12**. 17](#__RefHeading___Toc455651546)

[**Figure S21.** ^1^H-^1^H COSY spectrum (500 MHz, C_5_D_5_N) of compound **12**. 17](#__RefHeading___Toc455651547)

[**Figure S22.** NOESY spectrum (500 MHz, C_5_D_5_N) of compound **12.** 18](#__RefHeading___Toc455651548)

[**Figure S23.** HRESIMS spectrum of compound **12.** 18](#__RefHeading___Toc455651549)

[**Figure S24.** IR (KBr disc) spectrum of compound **12.** 19](#__RefHeading___Toc455651550)

[**Figure S25.** ^1^H-NMR spectrum (500 MHz, C_5_D_5_N) of compound **13**. 19](#__RefHeading___Toc455651551)

[**Figure S26.** ^13^C-NMR spectrum (126 MHz, C_5_D_5_N) of compound **13**. 20](#__RefHeading___Toc455651552)

[**Figure S27.** DEPT135 spectrum (126 MHz, C_5_D_5_N) of compound **13**. 20](#__RefHeading___Toc455651553)

[**Figure S28**. HSQC spectrum (500 MHz, C_5_D_5_N) of compound **13**. 21](#__RefHeading___Toc455651554)

[**Figure S29**. HMBC spectrum (500 MHz, C_5_D_5_N) of compound **13**. 21](#__RefHeading___Toc455651555)

[**Figure S30.** NOESY spectrum (500 MHz, C_5_D_5_N) of compound **13.** 22](#__RefHeading___Toc455651556)

[**Figure S31.** HRESIMS spectrum of compound **13.** 22](#__RefHeading___Toc455651557)

[**Figure S32.** IR (KBr disc) spectrum of compound **13.** 23](#__RefHeading___Toc455651558)

[**Figure S33.** ^1^H-NMR spectrum (500 MHz, C_5_D_5_N) of compound **16**. 23](#__RefHeading___Toc455651559)

[**Figure S34.** ^13^C-NMR spectrum (126 MHz, C_5_D_5_N) of compound **16**. 24](#__RefHeading___Toc455651560)

[**Figure S35**. HSQC spectrum (500 MHz, C_5_D_5_N) of compound **16**. 24](#__RefHeading___Toc455651561)

[**Figure S36**. HMBC spectrum (500 MHz, C_5_D_5_N) of compound **16**. 25](#__RefHeading___Toc455651562)

[**Figure S37.** ^1^H-^1^H COSY spectrum (500 MHz, C_5_D_5_N) of compound **16**. 25](#__RefHeading___Toc455651563)

[**Figure S38.** NOESY spectrum (500 MHz, C_5_D_5_N) of compound **16.** 26](#__RefHeading___Toc455651564)

[**Figure S39.** HRESIMS spectrum of compound **16.** 26](#__RefHeading___Toc455651565)

[**Figure S40.** IR (KBr disc) spectrum of compound **16.** 27](#__RefHeading___Toc455651566)

[**Figure S41.** ^1^H-NMR spectrum (500 MHz, C_5_D_5_N) of compound **17** 27](#__RefHeading___Toc455651567)

[**Figure S42.** ^13^C-NMR spectrum (126 MHz, C_5_D_5_N) of compound **17**. 28](#__RefHeading___Toc455651568)

[**Figure S43.** HSQC spectrum (500 MHz, C_5_D_5_N) of compound **17**. 28](#__RefHeading___Toc455651569)

[**Figure S44.** HMBC spectrum (500 MHz, C_5_D_5_N) of compound **17**. 29](#__RefHeading___Toc455651570)

[**Figure S45.** ^1^H-^1^H COSY spectrum (500 MHz, C_5_D_5_N) of compound **17**. 29](#__RefHeading___Toc455651571)

[**Figure S46.** NOESY spectrum (500 MHz, C_5_D_5_N) of compound **17**. 30](#__RefHeading___Toc455651572)

[**Figure S47.** HRHRESIMS spectrum of compound **17**. 31](#__RefHeading___Toc455651573)

[**Figure S48.** IR (KBr disc) spectrum of compound **17.** 31](#__RefHeading___Toc455651574)

[**Figure S49.** ^1^H-NMR spectrum (500 MHz, C_5_D_5_N) of compound **18**. 31](#__RefHeading___Toc455651575)

[**Figure S50.** ^13^C-NMR spectrum (126 MHz, C_5_D_5_N) of compound **18**. 32](#__RefHeading___Toc455651576)

[**Figure S51**. HSQC spectrum (500 MHz, C_5_D_5_N) of compound **18**. 32](#__RefHeading___Toc455651577)

[**Figure S52.** HMBC spectrum (500 MHz, C_5_D_5_N) of compound **18**. 33](#__RefHeading___Toc455651578)

[**Figure S53.** ^1^H-^1^H COSY spectrum (500 MHz, C_5_D_5_N) of compound **18**. 33](#__RefHeading___Toc455651579)

[**Figure S54.** NOESY spectrum (500 MHz, C_5_D_5_N) of compound **18**. 34](#__RefHeading___Toc455651580)

[**Figure S55.** HRESIMS spectrum of compound **18**. 34](#__RefHeading___Toc455651581)

[**Figure S56.** IR (KBr disc) spectrum of compound **18.** 35](#__RefHeading___Toc455651582)

[**Figure S57.** ^1^H-NMR spectrum (500 MHz, C_5_D_5_N) of compound **19**. 35](#__RefHeading___Toc455651583)

[**Figure S58.** ^13^C-NMR spectrum (126 MHz, C_5_D_5_N) of compound **19**. 36](#__RefHeading___Toc455651584)

[**Figure S59**. HSQC spectrum (500 MHz, C_5_D_5_N) of compound **19**. 36](#__RefHeading___Toc455651585)

[**Figure S60**. HMBC spectrum (500 MHz, C_5_D_5_N) of compound **19**. 37](#__RefHeading___Toc455651586)

[**Figure S61.** ^1^H-^1^H COSY spectrum (500 MHz, C_5_D_5_N) of compound **19**. 37](#__RefHeading___Toc455651587)

[**Figure S62.** NOESY spectrum (500 MHz, C_5_D_5_N) of compound **19**. 38](#__RefHeading___Toc455651588)

[**Figure S63.** HRESIMS spectrum of compound **19**. 38](#__RefHeading___Toc455651589)

[**Figure S64.** IR (KBr disc) spectrum of compound **19.** 39](#__RefHeading___Toc455651590)

[**Figure S65.** ^1^H-NMR spectrum (500 MHz, C_5_D_5_N) of compound **20**. 39](#__RefHeading___Toc455651591)

[**Figure S66.** ^13^C-NMR spectrum (126 MHz, C_5_D_5_N) of compound **20**. 40](#__RefHeading___Toc455651592)

[**Figure S67**. HSQC spectrum (500 MHz, C_5_D_5_N) of compound **20**. 40](#__RefHeading___Toc455651593)

[**Figure S68**. HMBC spectrum (500 MHz, C_5_D_5_N) of compound **20**. 41](#__RefHeading___Toc455651594)

[**Figure S69.** ^1^H-^1^H COSY spectrum (500 MHz, C_5_D_5_N) of compound **20**. 41](#__RefHeading___Toc455651595)

[**Figure S70.** NOESY spectrum (500 MHz, C_5_D_5_N) of compound **20.** 42](#__RefHeading___Toc455651596)

[**Figure S71.** HRESIMS spectrum of compound **20.** 42](#__RefHeading___Toc455651597)

[**Figure S72.** IR (KBr disc) spectrum of compound **20.** 43](#__RefHeading___Toc455651598)

**Table S1. ^13^C NMR data (126 MHz, pyridine-*d_5_*) for the known compounds**

| Position | **3** | **4** | **5** | **6** | **7** | **8** | **9** | **10** | **11** | **14** | **15** | **21** | **22** | **23** |
| --- | --- | --- | --- | --- | --- | --- | --- | --- | --- | --- | --- | --- | --- | --- |
| 1 | 73.5 | 78.5 | 78.3 | 78.2 | 78.2 | 76.8 | 79.9 | 80.0 | 76.0 | 75.2 | 73.8 | 31.3 | 31.3 | 73.7 |
| 2 | 33.9 | 68.6 | 67.8 | 68.4 | 68.4 | 68.2 | 67.5 | 67.5 | 71.4 | 36.9 | 34.1 | 27.3 | 27.3 | 33.2 |
| 3 | 71.9 | 76.0 | 76.0 | 76.6 | 76.6 | 75.8 | 75.9 | 76.0 | 73.2 | 69.2 | 67.7 | 75.2 | 74.9 | 68.5 |
| 4 | 68.1 | 67.8 | 68.6 | 67.9 | 68.0 | 71.5 | 69.9 | 70.1 | 75.2 | 74.4 | 36.8 | 30.9 | 31.0 | 34.7 |
| 5 | 87.9 | 78.3 | 78.6 | 87.8 | 87.8 | 86.6 | 79.0 | 78.6 | 86.5 | 79.7 | 83.0 | 37.3 | 37.3 | 31.5 |
| 6 | 24.8 | 30.7 | 30.7 | 25.3 | 25.3 | 211.5 | 70.0 | 73.9 | 211.2 | 30.5 | 31.8 | 27.3 | 27.3 | 27.0 |
| 7 | 28.6 | 28.8 | 28.8 | 28.8 | 28.8 | 75.5 | 35.7 | 72.1 | 75.4 | 28.5 | 29.5 | 27.1 | 27.1 | 26.9 |
| 8 | 34.9 | 35.2 | 35.2 | 35.0 | 35.0 | 41.2 | 30.4 | 34.9 | 38.2 | 35.4 | 34.9 | 35.9 | 35.9 | 36.0 |
| 9 | 46.7 | 45.7 | 45.7 | 47.0 | 47.0 | 38.3 | 45.7 | 37.9 | 41.2 | 45.8 | 46.1 | 40.6 | 40.6 | 42.5 |
| 10 | 47.3 | 45.4 | 45.4 | 46.6 | 46.6 | 50.5 | 45.7 | 46.2 | 50.7 | 44.8 | 45.5 | 35.6 | 35.6 | 40.7 |
| 11 | 21.6 | 22.0 | 22.0 | 22.0 | 22.0 | 22.3 | 21.8 | 21.7 | 22.2 | 21.5 | 21.7 | 21.5 | 21.5 | 21.4 |
| 12 | 40.1 | 40.4 | 40.3 | 40.8 | 40.2 | 39.7 | 40.3 | 40.1 | 39.6 | 40.2 | 40.3 | 40.7 | 40.7 | 40.9 |
| 13 | 40.6 | 41.0 | 41.0 | 40.3 | 40.9 | 41.0 | 41.0 | 40.7 | 40.9 | 41.1 | 40.8 | 41.2 | 41.3 | 40.7 |
| 14 | 56.1 | 56.6 | 56.6 | 56.3 | 56.3 | 49.6 | 56.4 | 50.4 | 49.4 | 56.5 | 56.4 | 56.8 | 56.8 | 56.8 |
| 15 | 32.2 | 32.5 | 32.5 | 32.5 | 32.5 | 31.8 | 32.6 | 32.3 | 31.8 | 32.5 | 32.5 | 32.5 | 32.5 | 32.4 |
| 16 | 81.3 | 81.5 | 81.8 | 81.5 | 81.8 | 81.6 | 81.7 | 81.9 | 81.6 | 81.8 | 81.8 | 81.7 | 81.6 | 81.9 |
| 17 | 63.0 | 63.2 | 63.4 | 63.1 | 63.3 | 63.2 | 63.4 | 63.4 | 63.2 | 63.3 | 63.3 | 63.3 | 63.5 | 62.9 |
| 18 | 16.6 | 17.0 | 16.9 | 16.9 | 16.8 | 16.6 | 16.9 | 16.7 | 16.5 | 16.9 | 16.8 | 17.0 | 17.0 | 17.1 |
| 19 | 13.8 | 14.2 | 14.2 | 14.1 | 14.1 | 13.3 | 16.5 | 15.3 | 13.2 | 14.4 | 14.0 | 24.2 | 24.2 | 19.7 |
| 20 | 42.6 | 42.8 | 42.2 | 42.8 | 42.2 | 42.3 | 42.2 | 42.4 | 42.3 | 42.2 | 42.3 | 42.8 | 42.4 | 42.5 |
| 21 | 14.9 | 15.2 | 15.3 | 15.2 | 15.3 | 15.3 | 15.3 | 15.9 | 15.3 | 15.4 | 15.3 | 15.3 | 15.4 | 15.2 |
| 22 | 109.8 | 110.1 | 109.8 | 110.1 | 109.8 | 109.8 | 109.8 | 109.8 | 109.8 | 109.8 | 109.8 | 110.1 | 109.6 | 112.1 |
| 23 | 26.3 | 26.7 | 33.6 | 26.7 | 33.6 | 33.5 | 33.5 | 33.5 | 29.3 | 33.6 | 33.6 | 26.8 | 32.2 | 40.9 |
| 24 | 26.5 | 26.5 | 29.3 | 26.5 | 29.3 | 29.3 | 29.3 | 29.3 | 33.5 | 29.3 | 29.3 | 26.6 | 29.6 | 81.9 |
| 25 | 27.6 | 27.9 | 144.8 | 27.9 | 144.7 | 144.7 | 144.8 | 144.8 | 144.7 | 144.8 | 144.8 | 27.9 | 30.9 | 38.5 |
| 26 | 65.2 | 65.5 | 65.4 | 65.5 | 65.4 | 65.3 | 65.4 | 65.3 | 65.3 | 65.4 | 65.4 | 65.5 | 67.2 | 65.4 |
| 27 | 16.4 | 16.6 | 109.1 | 16.6 | 109.1 | 109.1 | 109.1 | 109.0 | 109.1 | 109.1 | 109.1 | 16.7 | 17.7 | 13.9 |
|  | 5-O-Glc |  |  | 5-O-Glc | 5-O-Glc |  |  |  | 4-O-Xyl |  | 5-O-Glc | 3-O-Glc | 3-O-Glc | 24-O-Glc |
| 1 | 97.5 |  |  | 97.9 | 97.9 |  |  |  | 104.2 |  | 97.6 | 103.2 | 103.2 | 106.4 |
| 2 | 75.9 |  |  | 76.2 | 76.2 |  |  |  | 75.3 |  | 75.8 | 74.9 | 75.1 | 75.4 |
| 3 | 78.7 |  |  | 79.0 | 79.0 |  |  |  | 78.6 |  | 79.0 | 76.8 | 76.8 | 78.8 |
| 4 | 71.9 |  |  | 72.2 | 72.2 |  |  |  | 71.5 |  | 72.1 | 81.7 | 81.7 | 71.5 |
| 5 | 78.8 |  |  | 79.2 | 79.2 |  |  |  | 67.7 |  | 79.2 | 77.3 | 77.3 | 77.0 |
| 6 | 62.8 |  |  | 63.1 | 63.2 |  |  |  |  |  | 63.2 | 62.6 | 62.6 | 70.3 |
|  |  |  |  |  |  |  |  |  |  |  |  | Glc(1→4) | Glc(1→4) | Glc(1→6) |
| 1' |  |  |  |  |  |  |  |  |  |  |  | 105.4 | 105.3 | 105.8 |
| 2' |  |  |  |  |  |  |  |  |  |  |  | 75.2 | 75.2 | 75.8 |
| 3' |  |  |  |  |  |  |  |  |  |  |  | 78.8 | 78.8 | 78.6 |
| 4' |  |  |  |  |  |  |  |  |  |  |  | 71.8 | 71.8 | 71.8 |
| 5' |  |  |  |  |  |  |  |  |  |  |  | 78.6 | 78.6 | 78.8 |
| 6' |  |  |  |  |  |  |  |  |  |  |  | 62.8 | 62.7 | 63.0 |

**Table S2.** Antiproliferative activities of the extracts from *T. chinensis* against human cancer cell lines

|  | **IC_50_ (μg/mL)*^a^*** | | | | | | |
| --- | --- | --- | --- | --- | --- | --- | --- |
| **Sample** | **FaDu** | **Detroit 562** | **CNE-1** | **CNE-2** | **HepG2** | **K562** | **SPC-A-1** |
| **TB** | 10.6±1.0 | >50.0 | >50.0 | >50.0 | >50.0 | 23.0±0.9 | >50.0 |
| **TC** | 6.7±0.5 | 45.9±1.7 | >50.0 | >50.0 | >50.0 | 6.5±2.3 | >50.0 |
| **TD** | 29.3±1.5 | 27.5±1.3 | >50.0 | >50.0 | 21.4±2.2 | 20.9±0.6 | 22.1±0.9 |

*^a^* Values are presented as means ± SD (*n* = 3).

TB, 20% EtOH fraction of *T. chinensis* extract yield from D101 macroporous resin column; TC, 60% EtOH fraction of *T. chinensis* extract yield from D101 macroporous resin column; TD, 80% EtOH fraction of *T. chinensis* extract yield from D101 macroporous resin column.

**Table S3.** Inhibitory effects of the extracts from *T. chinensis* on NO production induced by LPS in macrophagesa

| **Sample** | **TB** | **TC** | **TD** |
| --- | --- | --- | --- |
| **IC_50_ (μg/mL)** | 32.6±3.7 | 37.5±5.4 | 36.6±1.8 |

TB, 20% EtOH fraction of *T. chinensis* extract yield from D101 macroporous resin column; TC, 60% EtOH fraction of *T. chinensis* extract yield from D101 macroporous resin column; TD, 80% EtOH fraction of *T. chinensis* extract yield from D101 macroporous resin column.

**Figure S1.** ^1^H-NMR spectrum (500 MHz, C_5_D_5_N) of compound **1**.


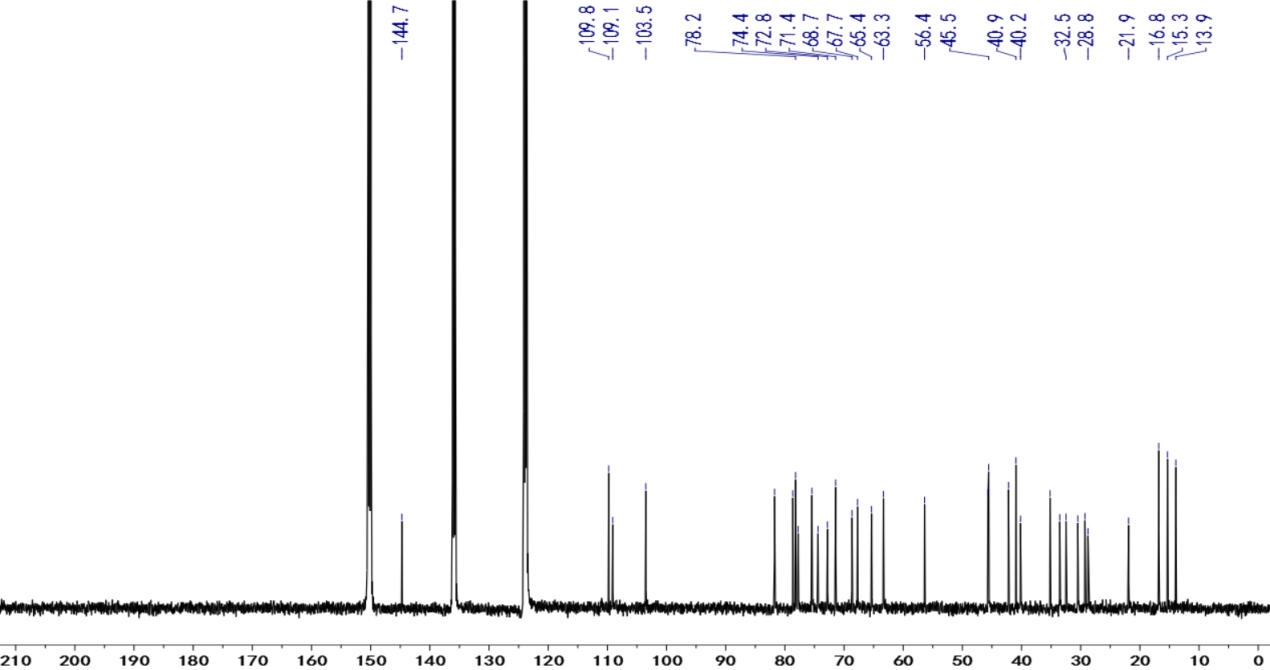


**Figure S2.** ^13^C-NMR spectrum (126 MHz, C_5_D_5_N) of compound **1**.


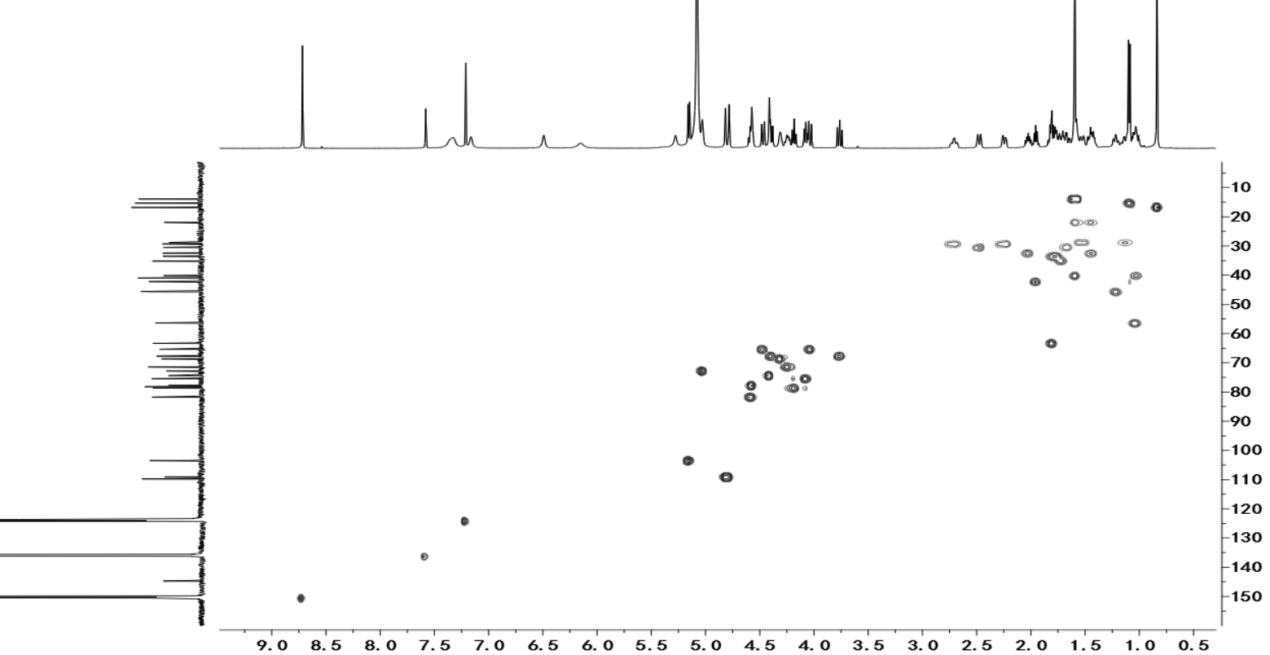


**Figure S3**. HSQC spectrum (500 MHz, C_5_D_5_N) of compound **1**.


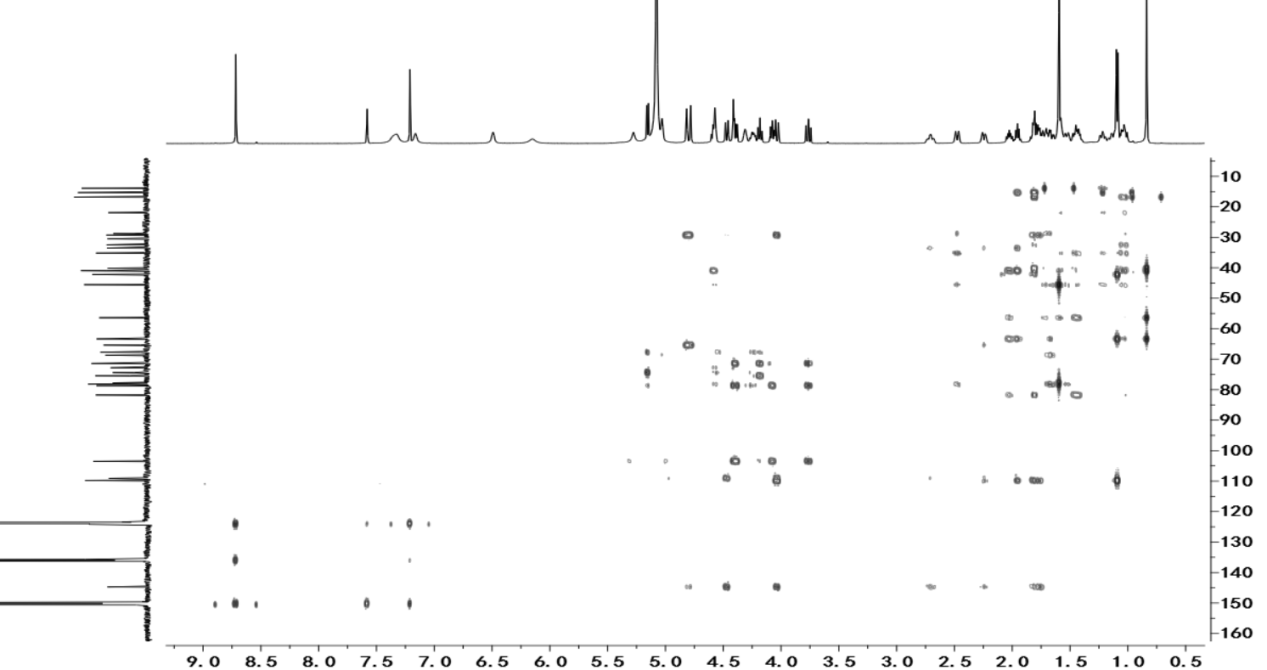


**Figure S4**. HMBC spectrum (500 MHz, C_5_D_5_N) of compound **1**.


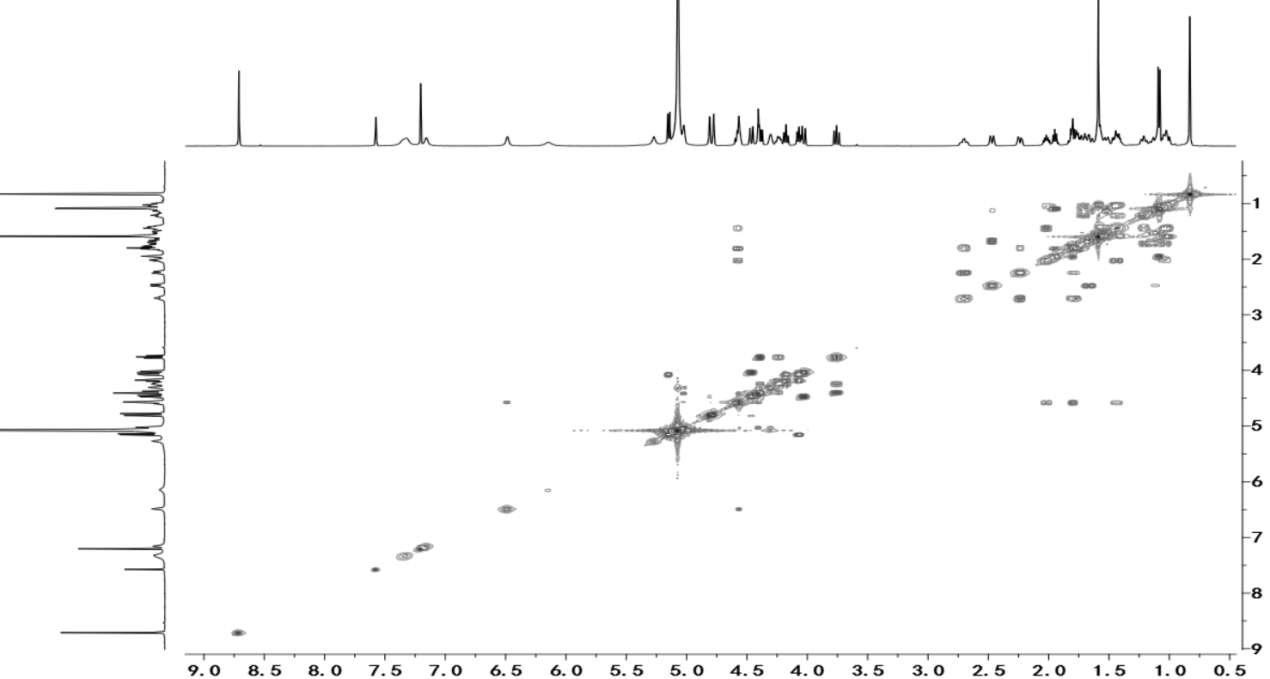


**Figure S5.** ^1^H-^1^H COSY spectrum (500 MHz, C_5_D_5_N) of compound **1**.


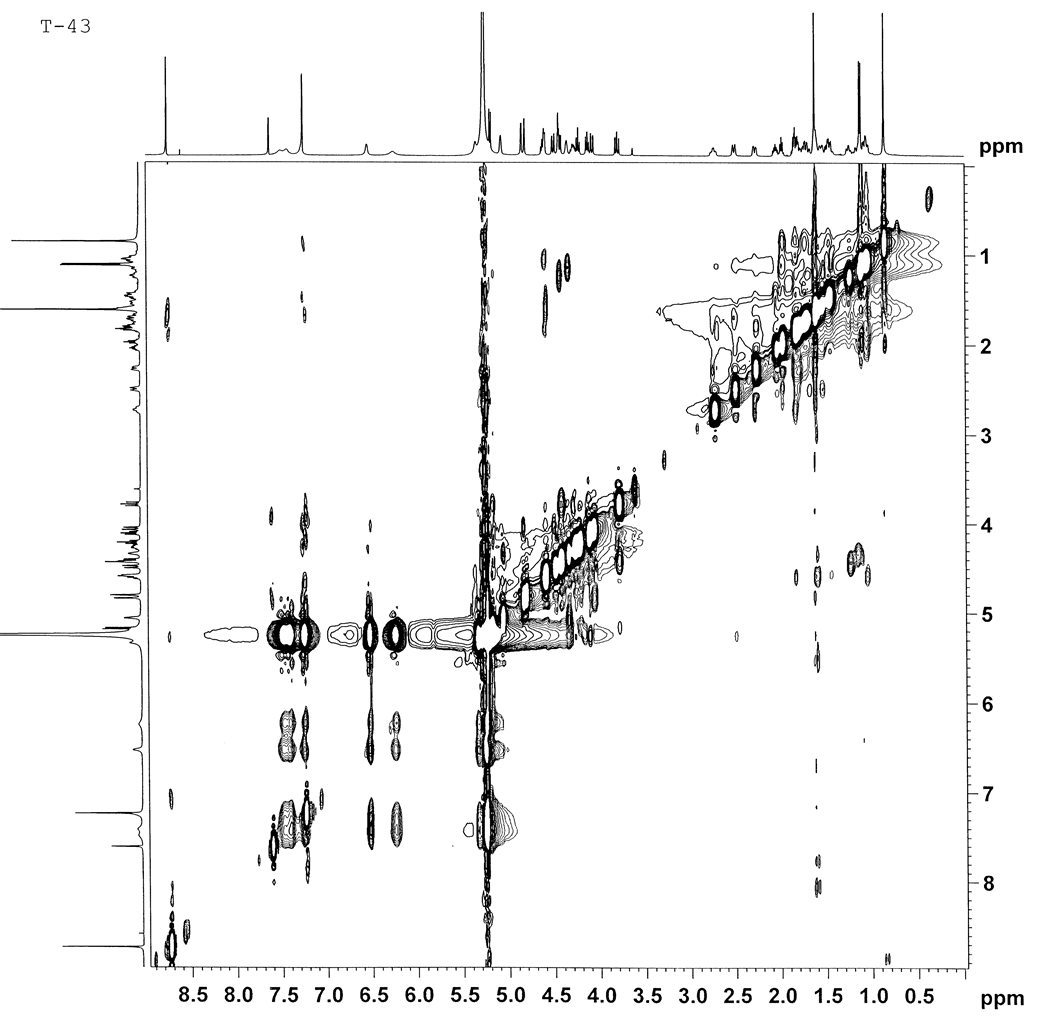


**Figure S6.** NOESY spectrum (500 MHz, C_5_D_5_N) of compound **1.**


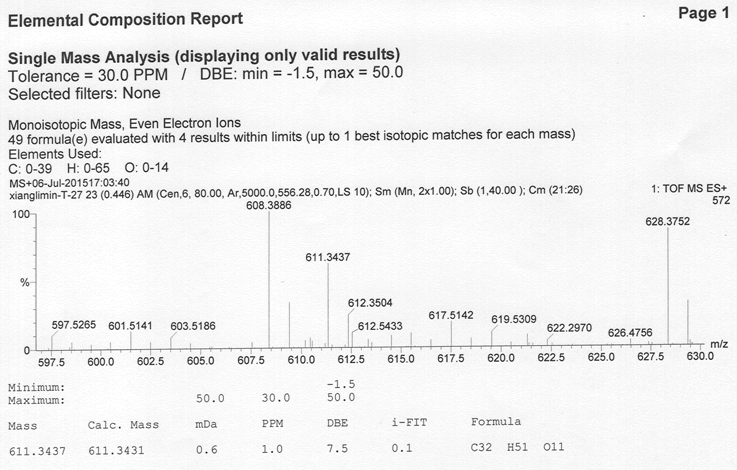


**Figure S7.** HRESIMS spectrum of compound **1.**

**Figure S8.** IR (KBr disc) spectrum of compound **1.**


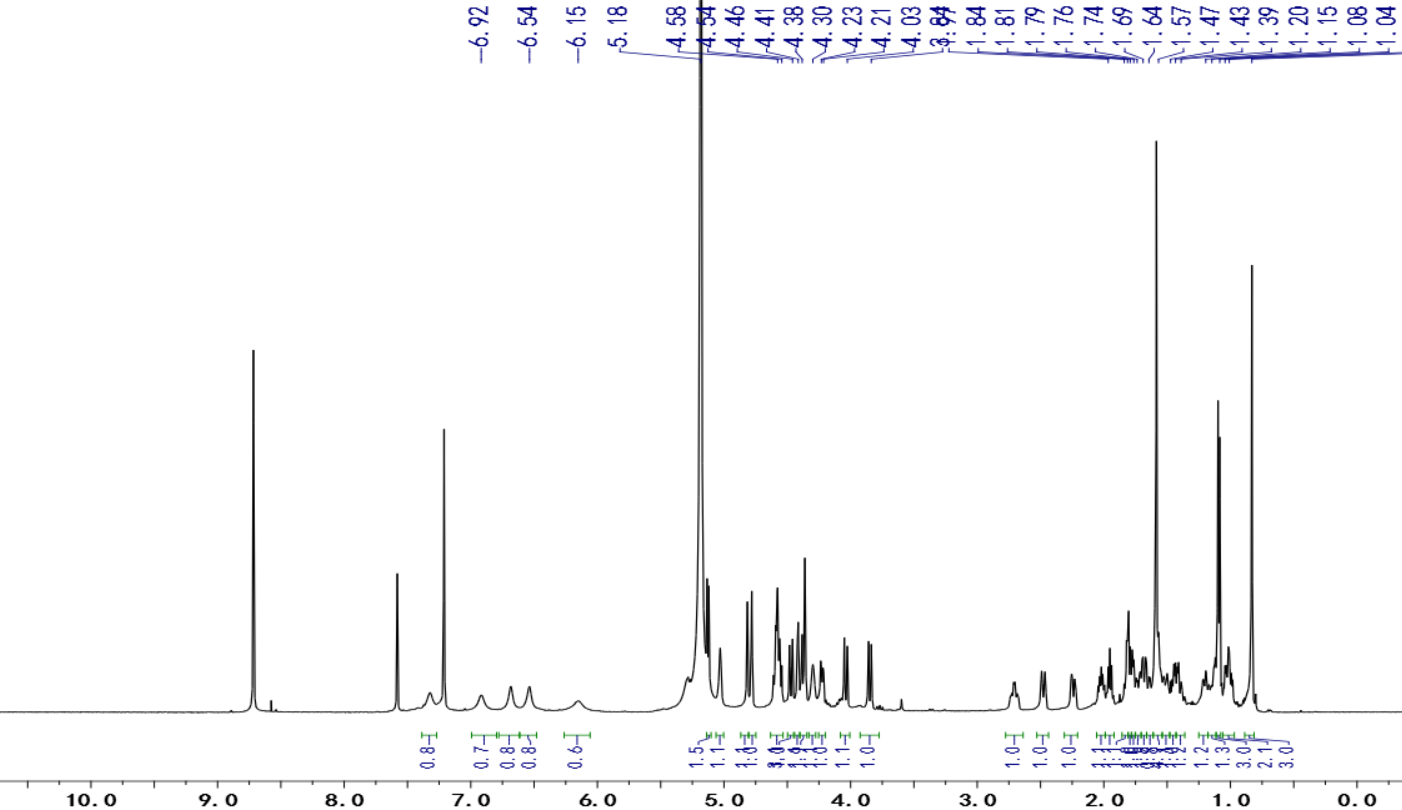


**Figure S9.** ^1^H-NMR spectrum (500 MHz, C_5_D_5_N) of compound **2**


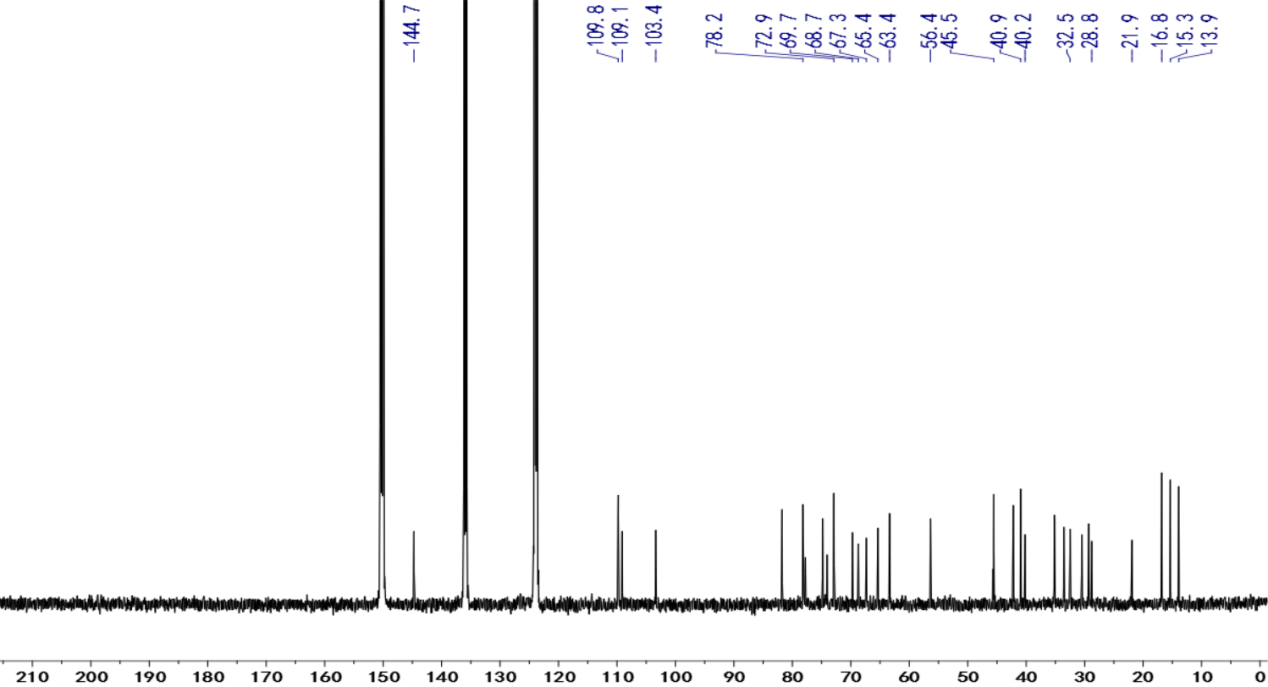


**Figure S10.** ^13^C-NMR spectrum (126 MHz, C_5_D_5_N) of compound **2**


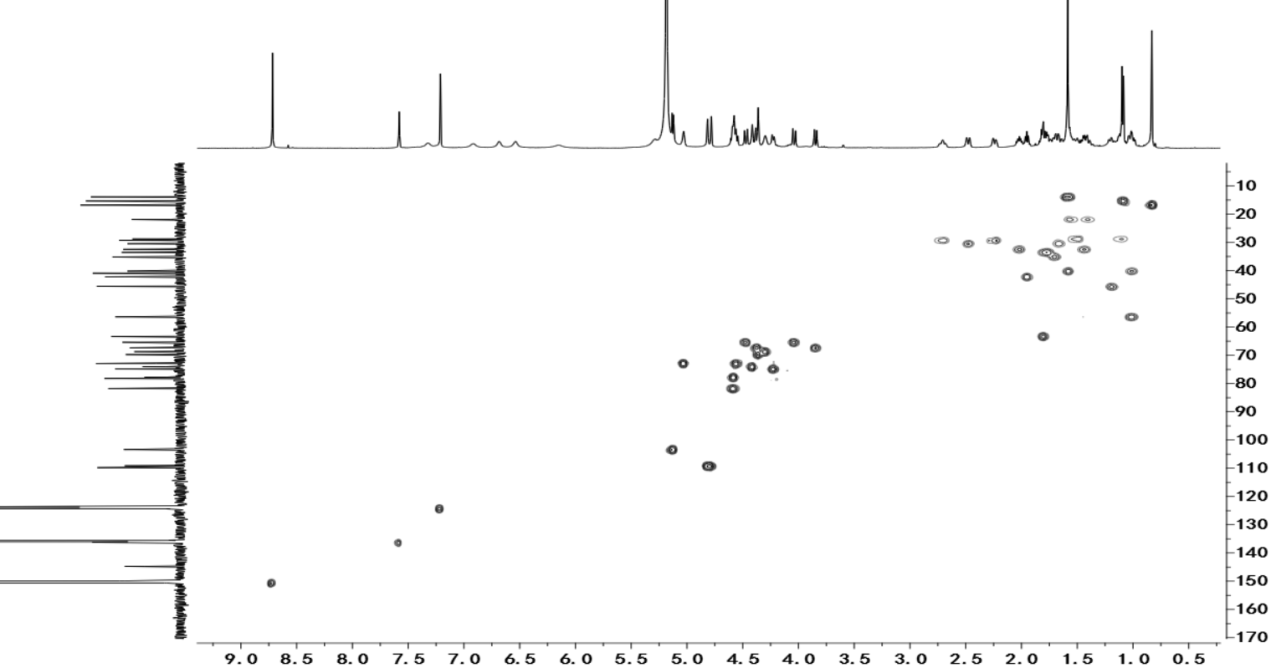


**Figure S11**. HSQC spectrum (500 MHz, C_5_D_5_N) of compound **2**


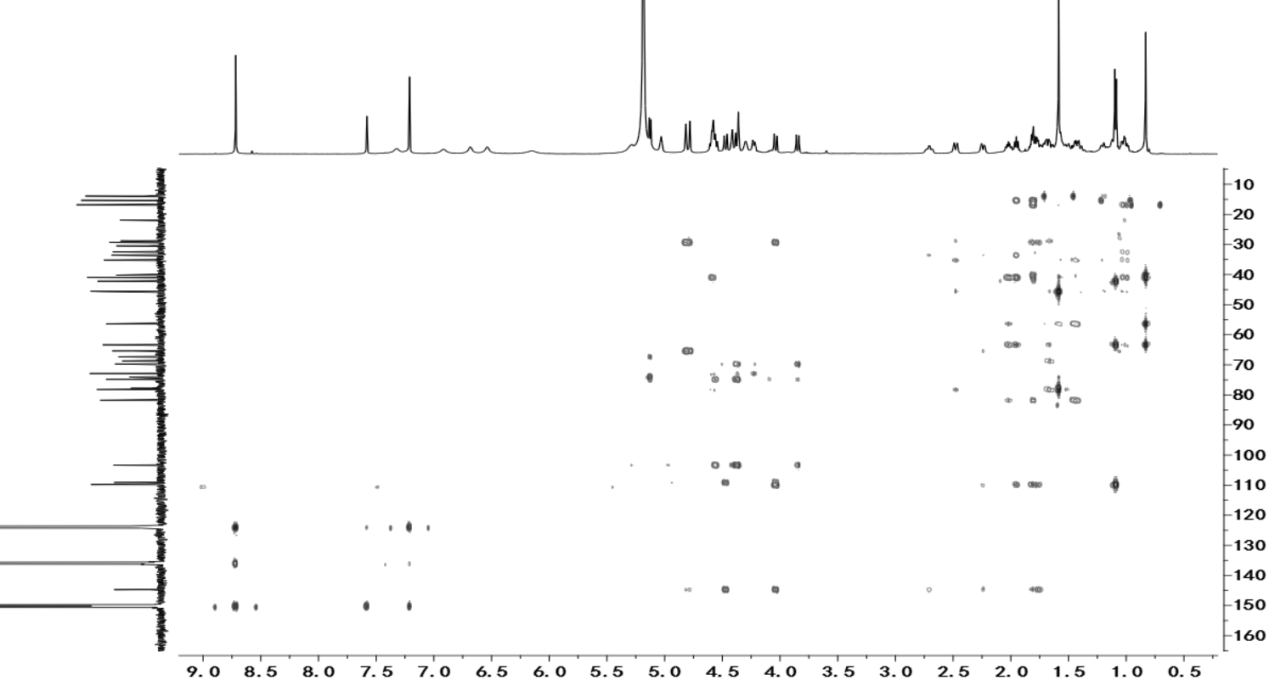


**Figure S12**. HMBC spectrum (500 MHz, C_5_D_5_N) of compound **2**


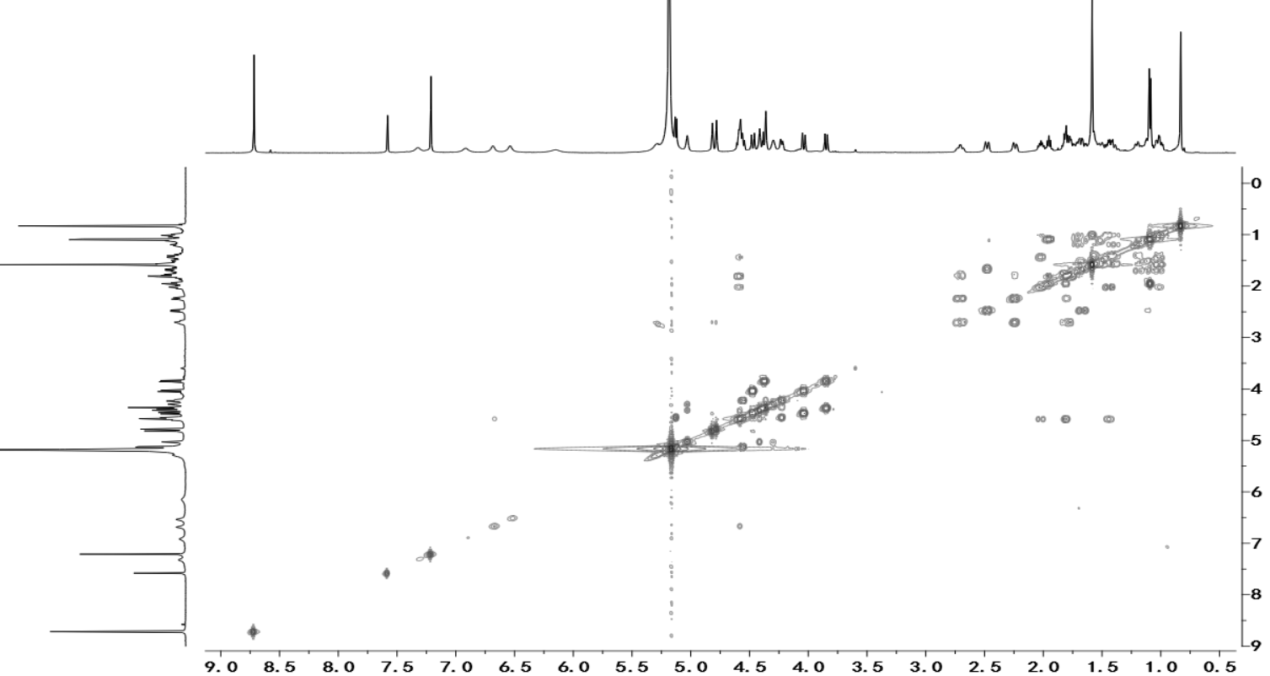


**Figure S13.** ^1^H-^1^H COSY spectrum (500 MHz, C_5_D_5_N) of compound **2**


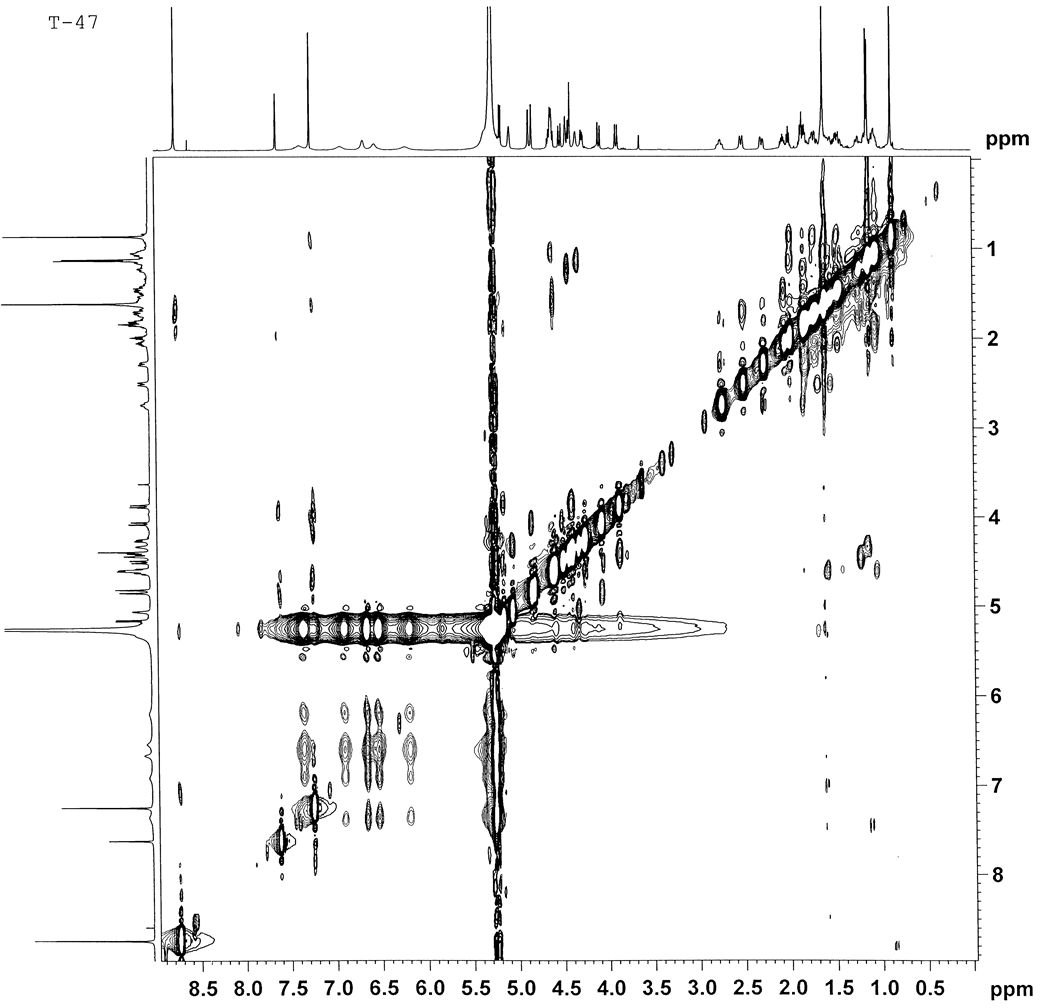


**Figure S14.** NOESY spectrum (500 MHz, C_5_D_5_N) of compound **2**


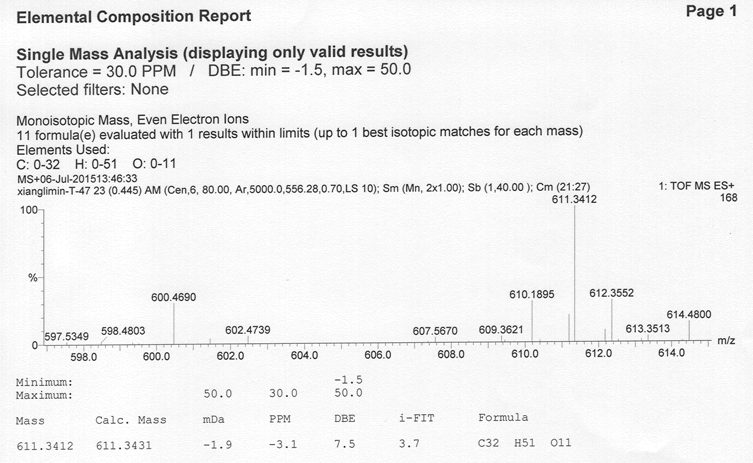


**Figure S15.** HRESIMS spectrum of compound **2**

**Figure S16.** IR (KBr disc) spectrum of compound **2**

**
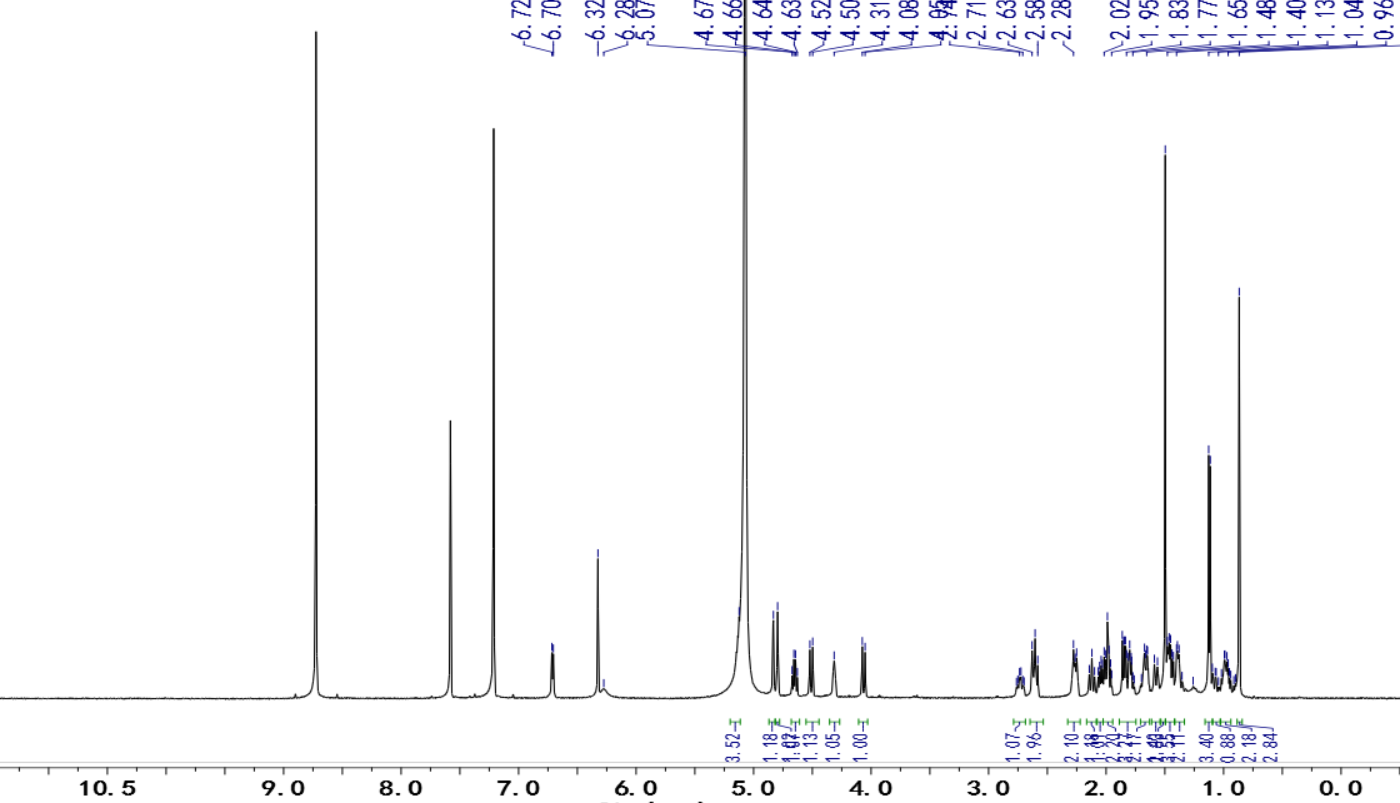
**

**Figure S17.** ^1^H-NMR spectrum (500 MHz, C_5_D_5_N) of compound **12**.


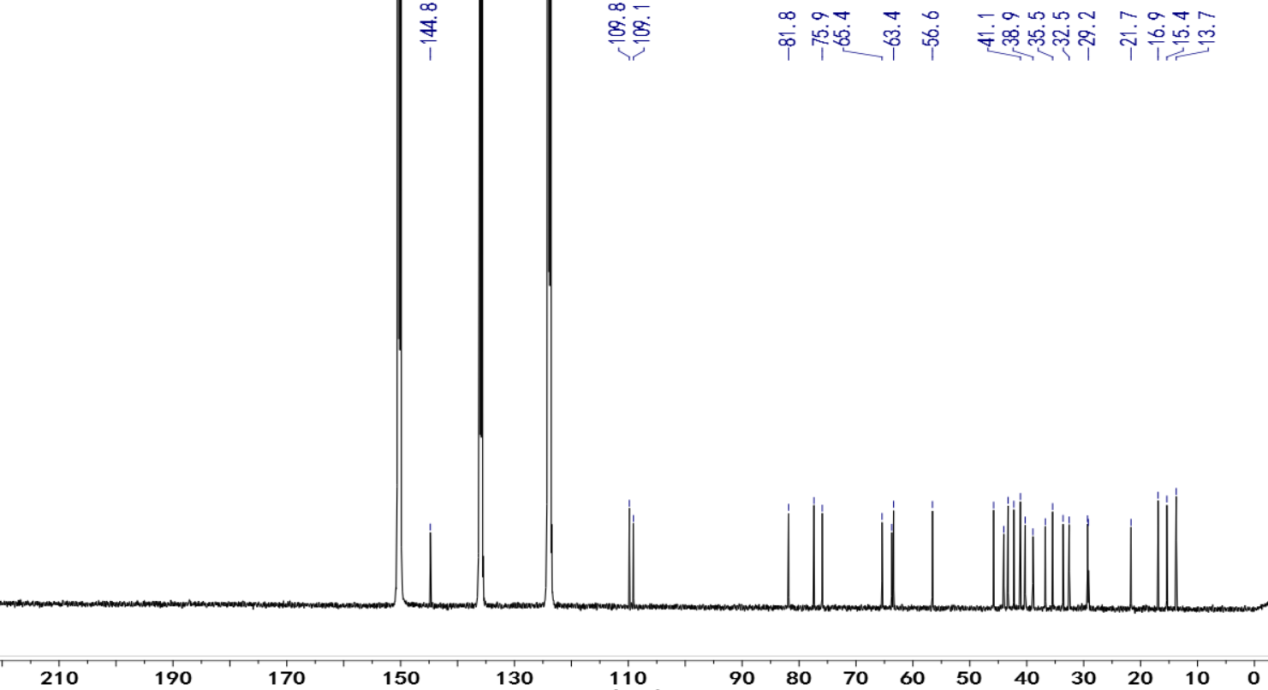


**Figure S18.** ^13^C-NMR spectrum (126 MHz, C_5_D_5_N) of compound **12**.


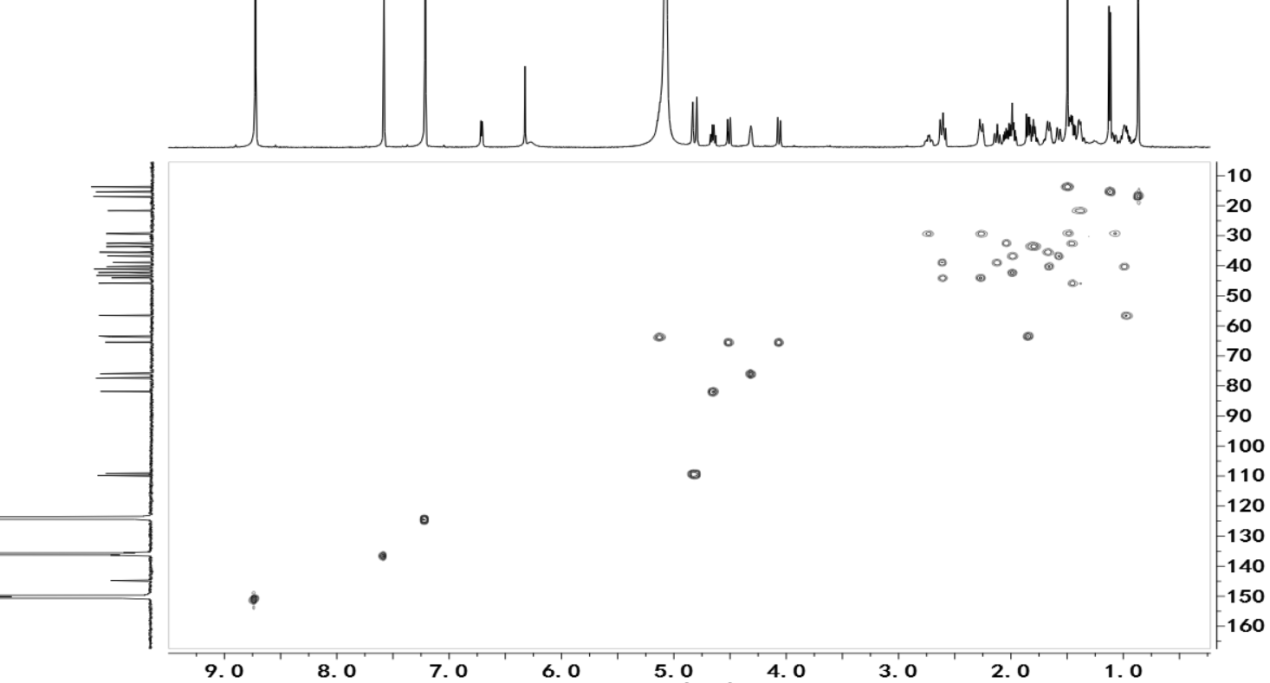


**Figure S19**. HSQC spectrum (500 MHz, C_5_D_5_N) of compound **12**.


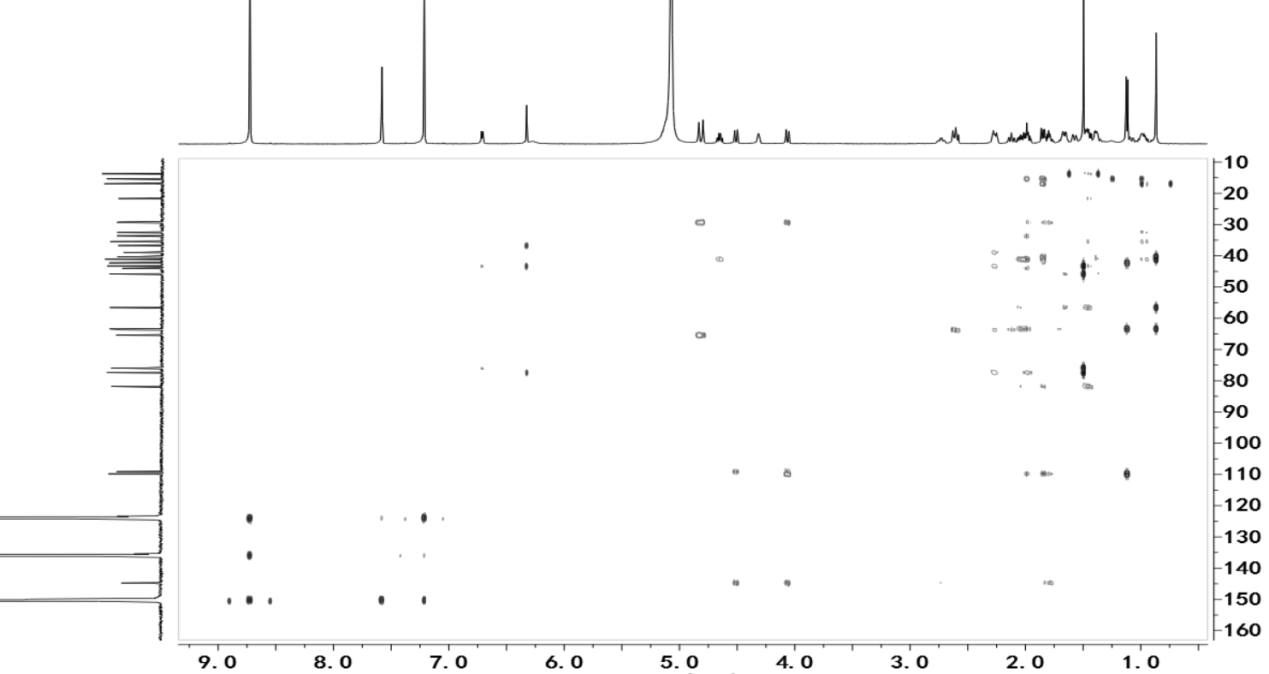


**Figure S20**. HMBC spectrum (500 MHz, C_5_D_5_N) of compound **12**.


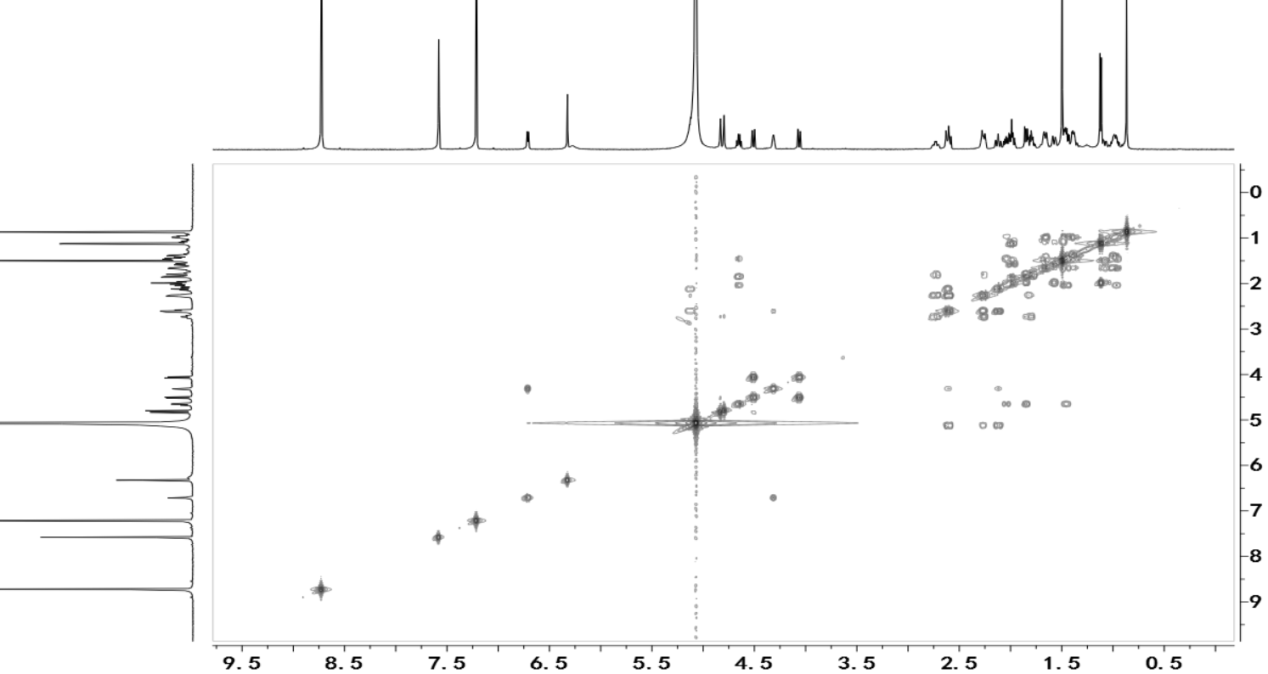


**Figure S21.** ^1^H-^1^H COSY spectrum (500 MHz, C_5_D_5_N) of compound **12**.


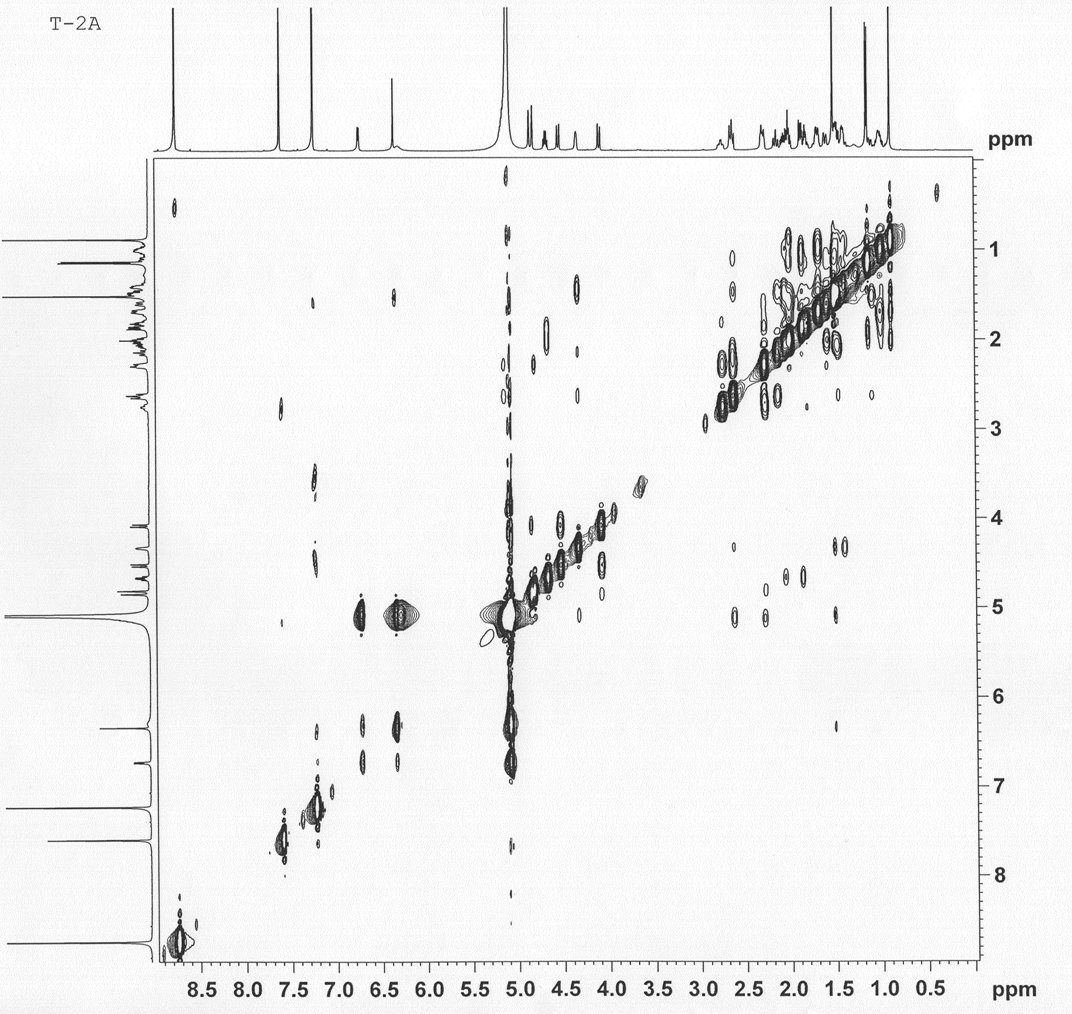


**Figure S22.** NOESY spectrum (500 MHz, C_5_D_5_N) of compound **12.**


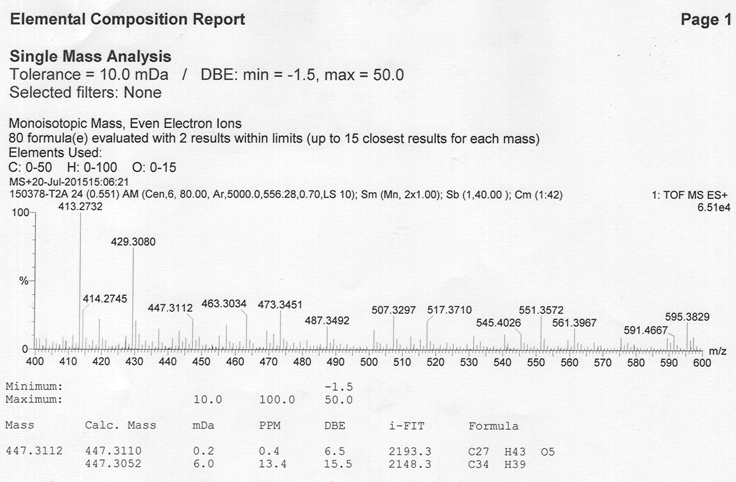


**Figure S23.** HRESIMS spectrum of compound **12.**

**Figure S24.** IR (KBr disc) spectrum of compound **12.**


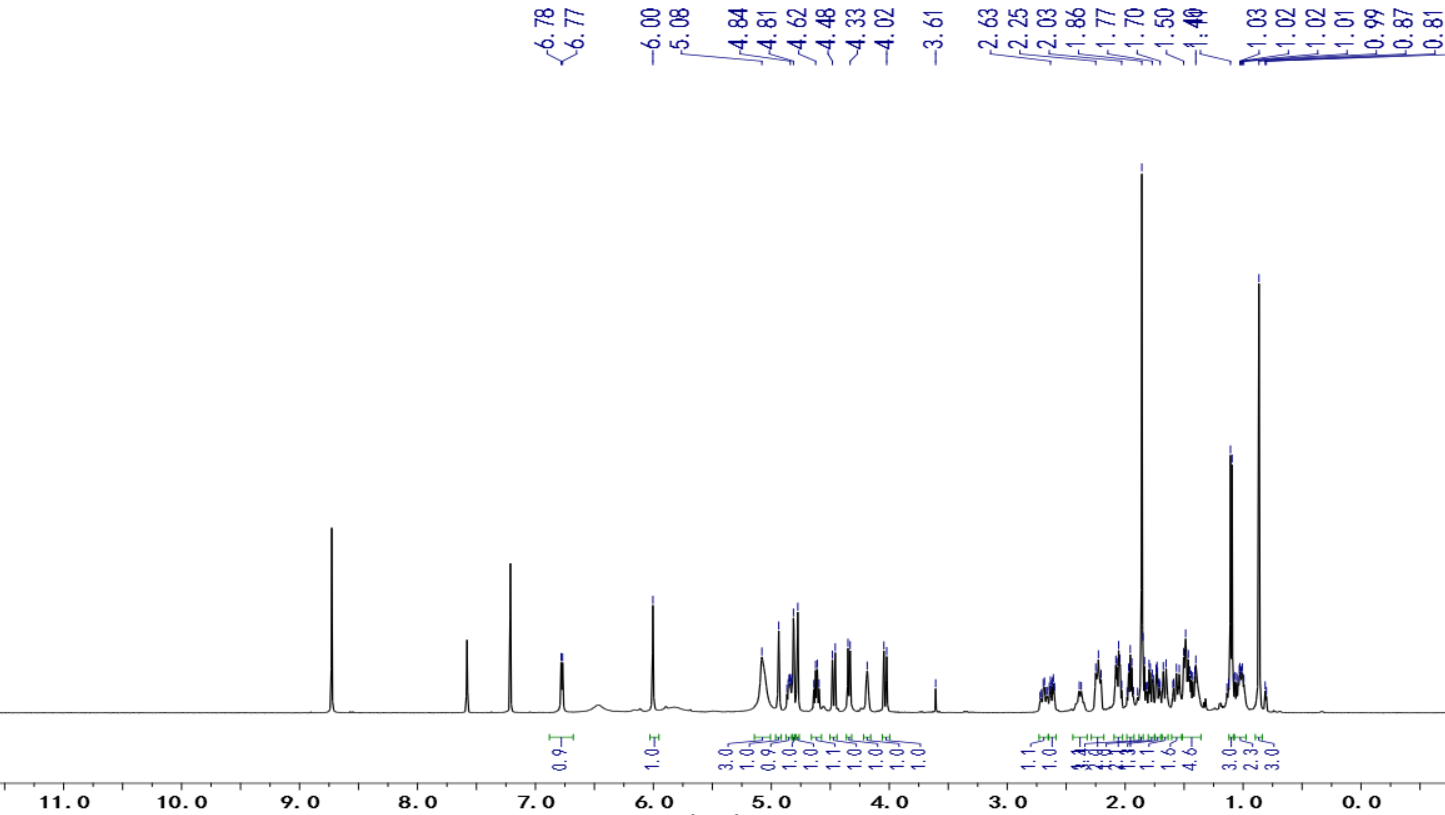


**Figure S25.** ^1^H-NMR spectrum (500 MHz, C_5_D_5_N) of compound **13**.


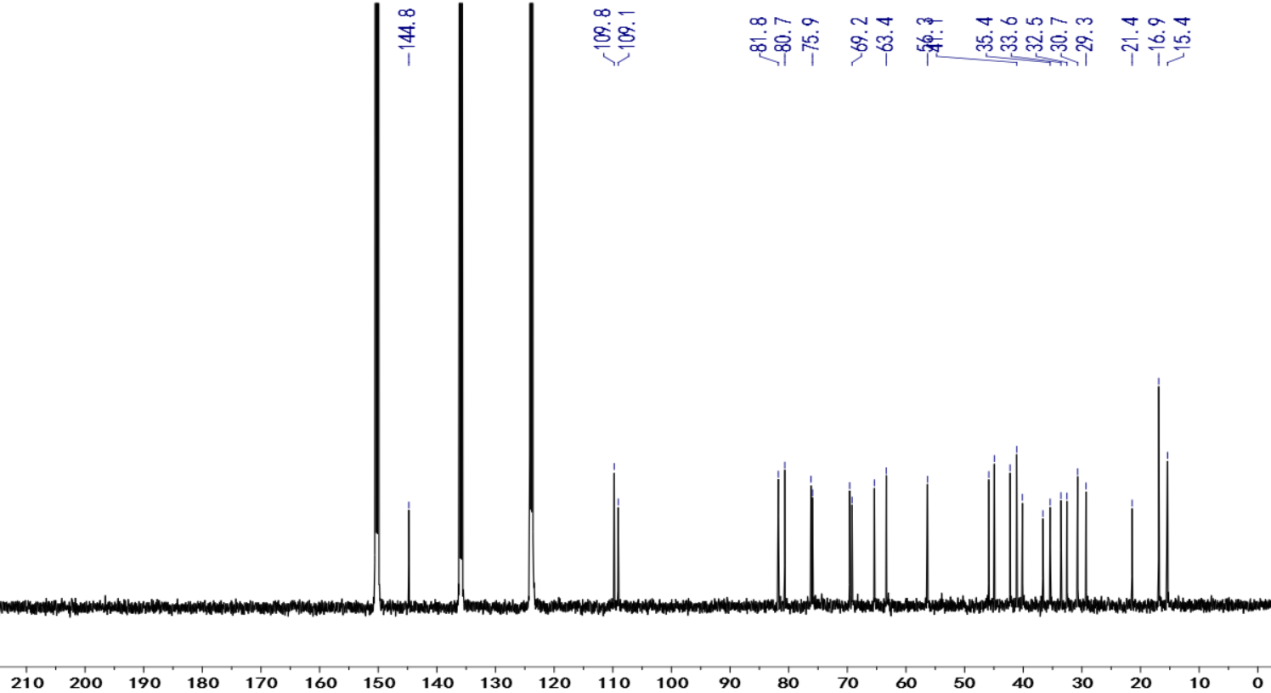


**Figure S26.** ^13^C-NMR spectrum (126 MHz, C_5_D_5_N) of compound **13**.


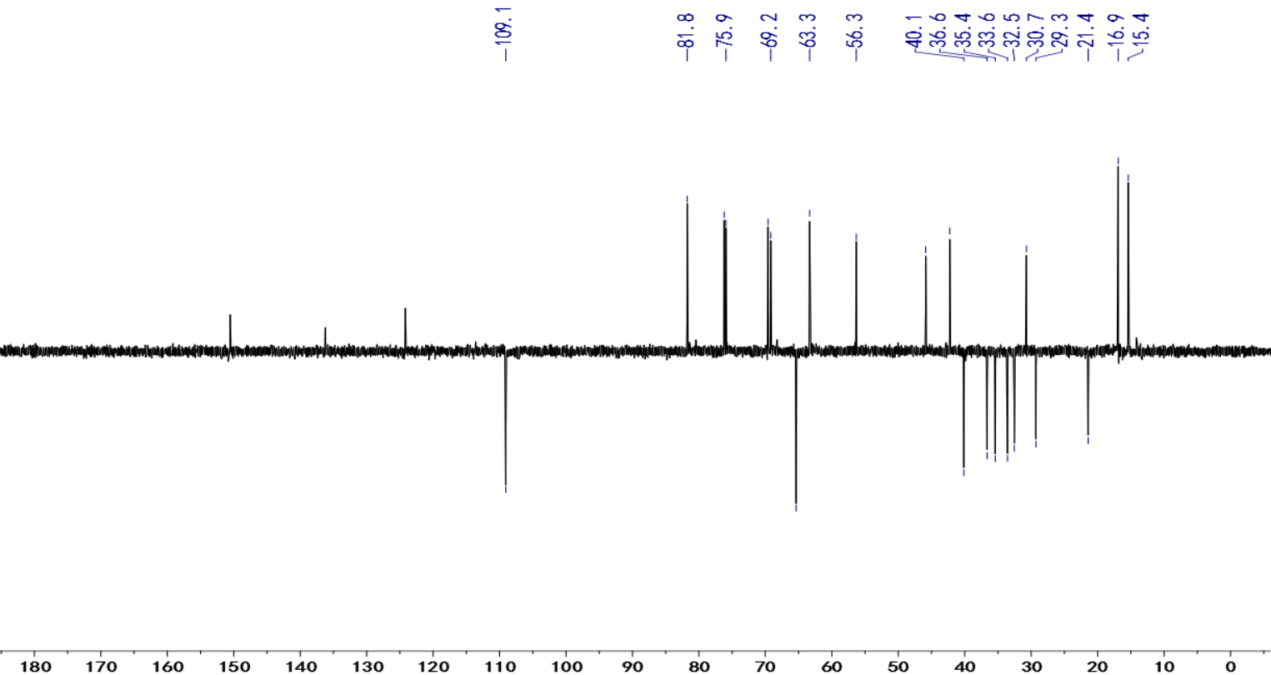


**Figure S27.** DEPT135 spectrum (126 MHz, C_5_D_5_N) of compound **13**.


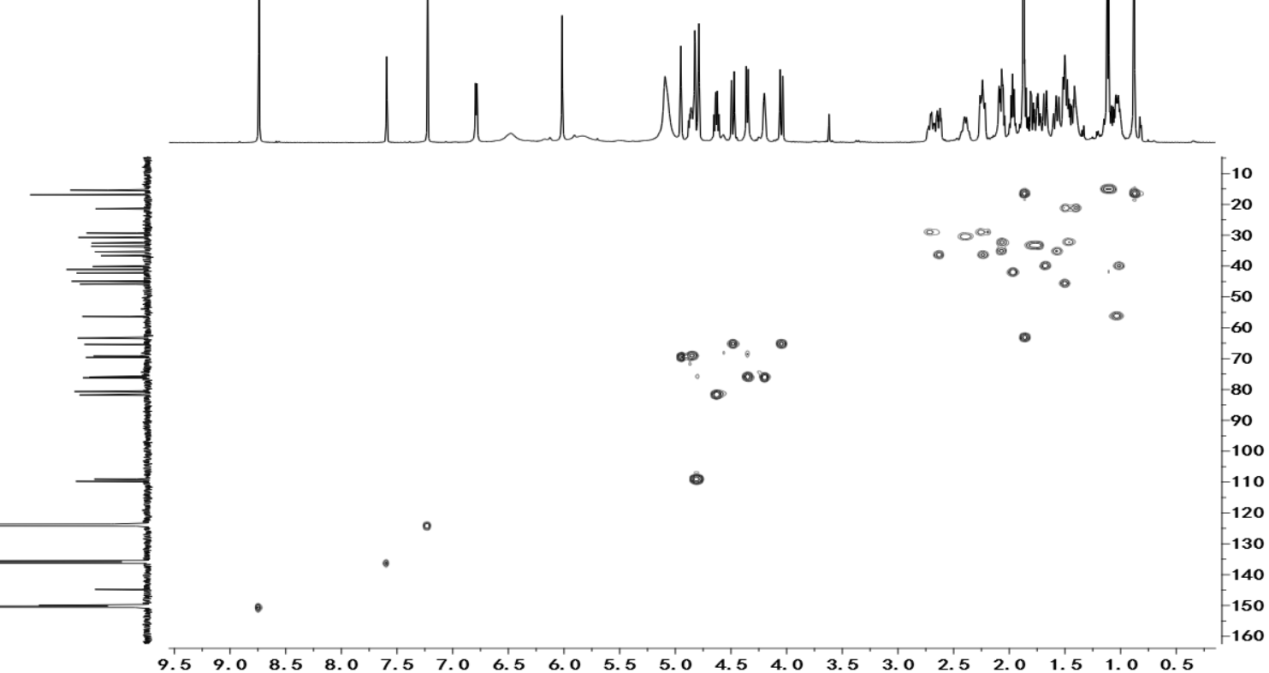


**Figure S28**. HSQC spectrum (500 MHz, C_5_D_5_N) of compound **13**.


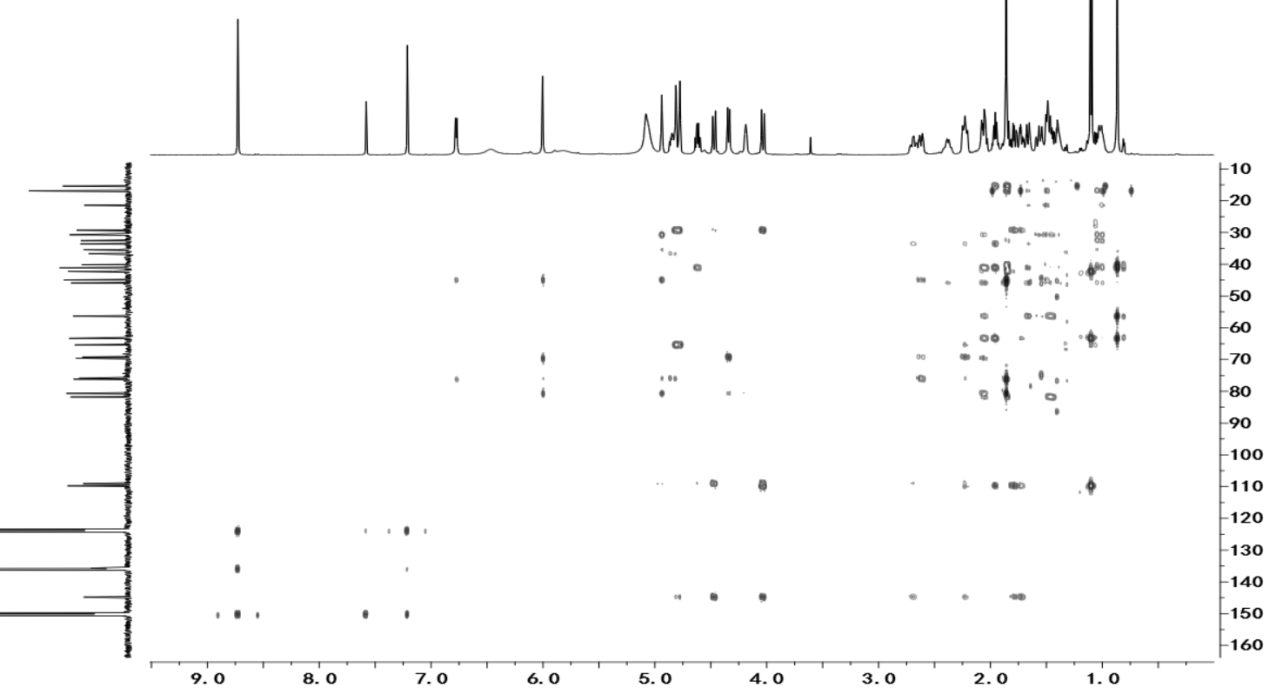


**Figure S29**. HMBC spectrum (500 MHz, C_5_D_5_N) of compound **13**.


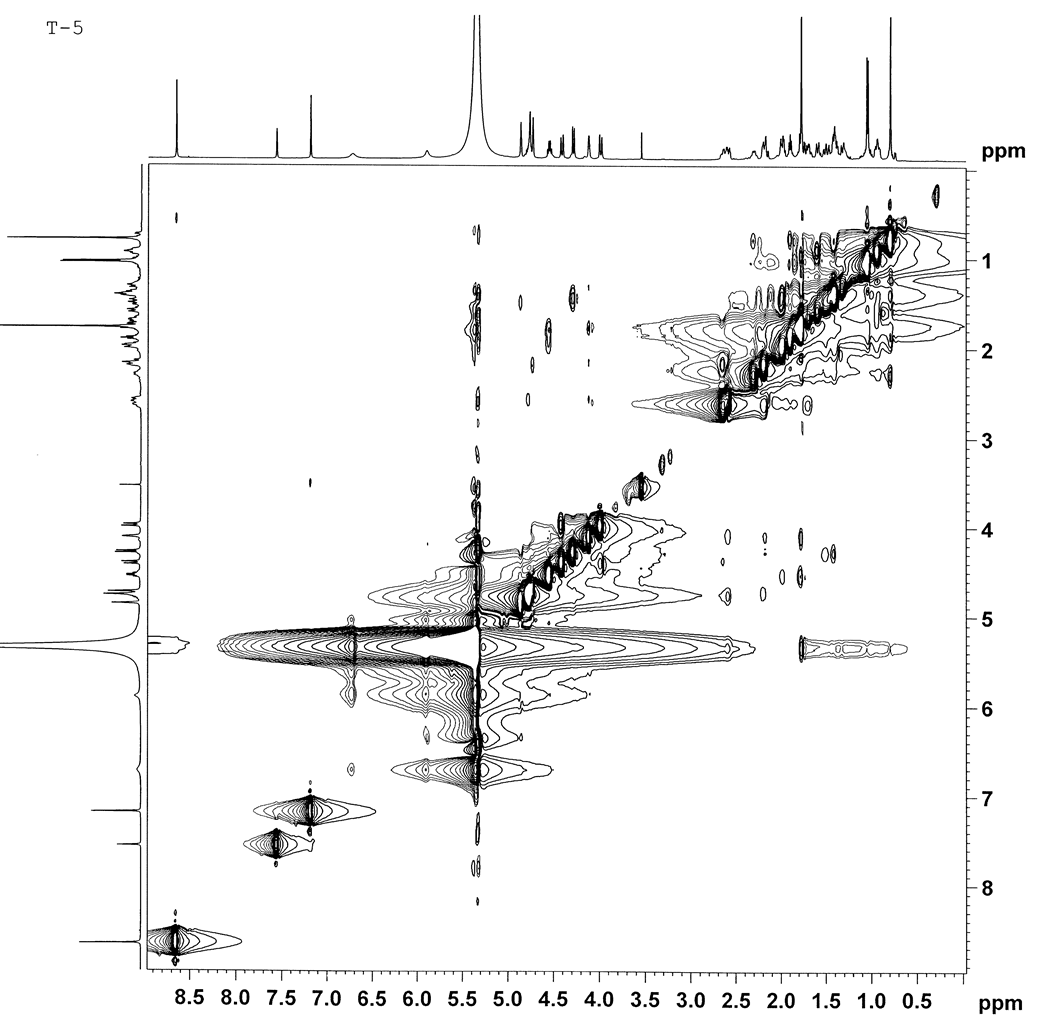


**Figure S30.** NOESY spectrum (500 MHz, C_5_D_5_N) of compound **13.**


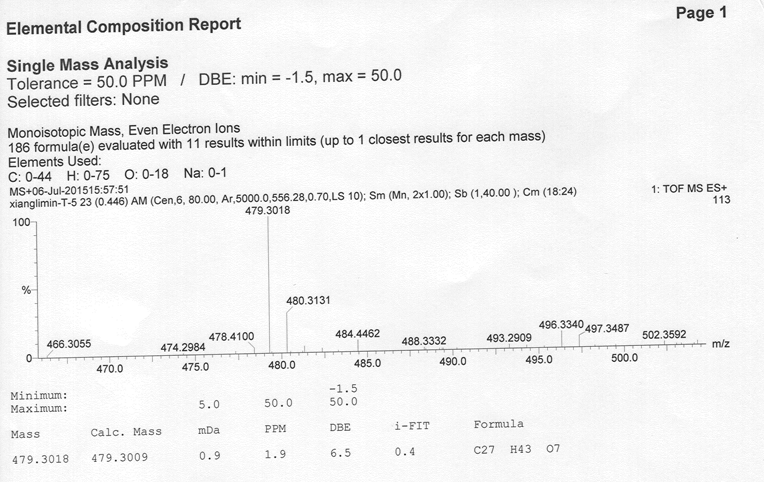


**Figure S31.** HRESIMS spectrum of compound **13.**

**Figure S32.** IR (KBr disc) spectrum of compound **13.**


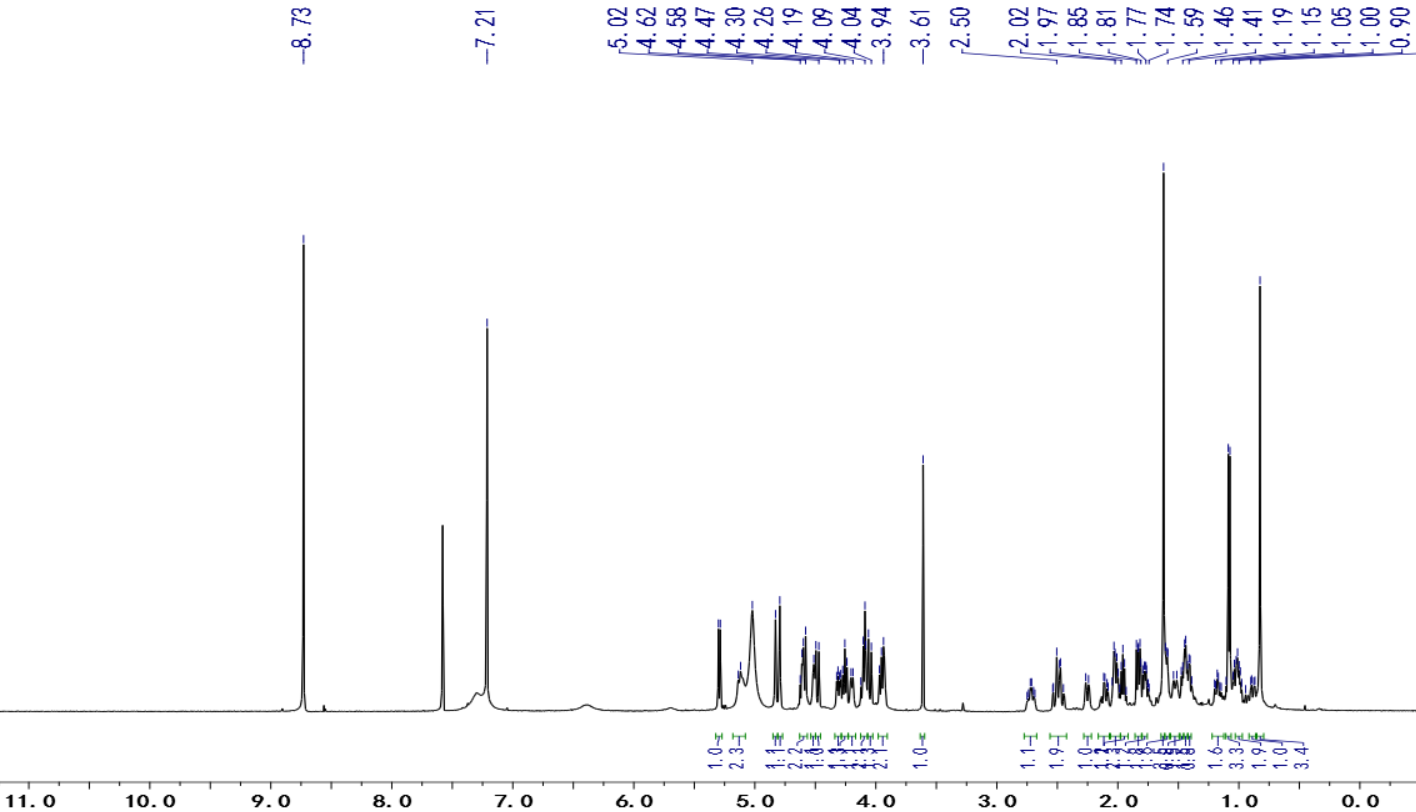


**Figure S33.** ^1^H-NMR spectrum (500 MHz, C_5_D_5_N) of compound **16**.


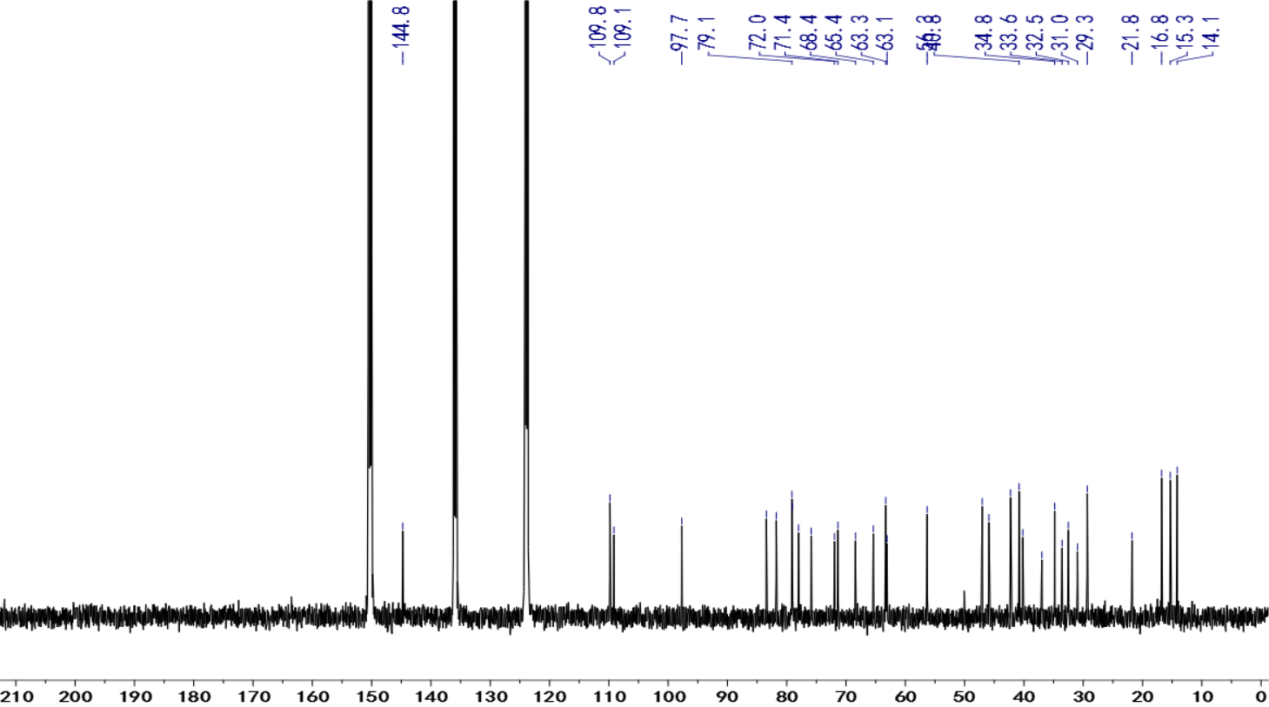


**Figure S34.** ^13^C-NMR spectrum (126 MHz, C_5_D_5_N) of compound **16**.


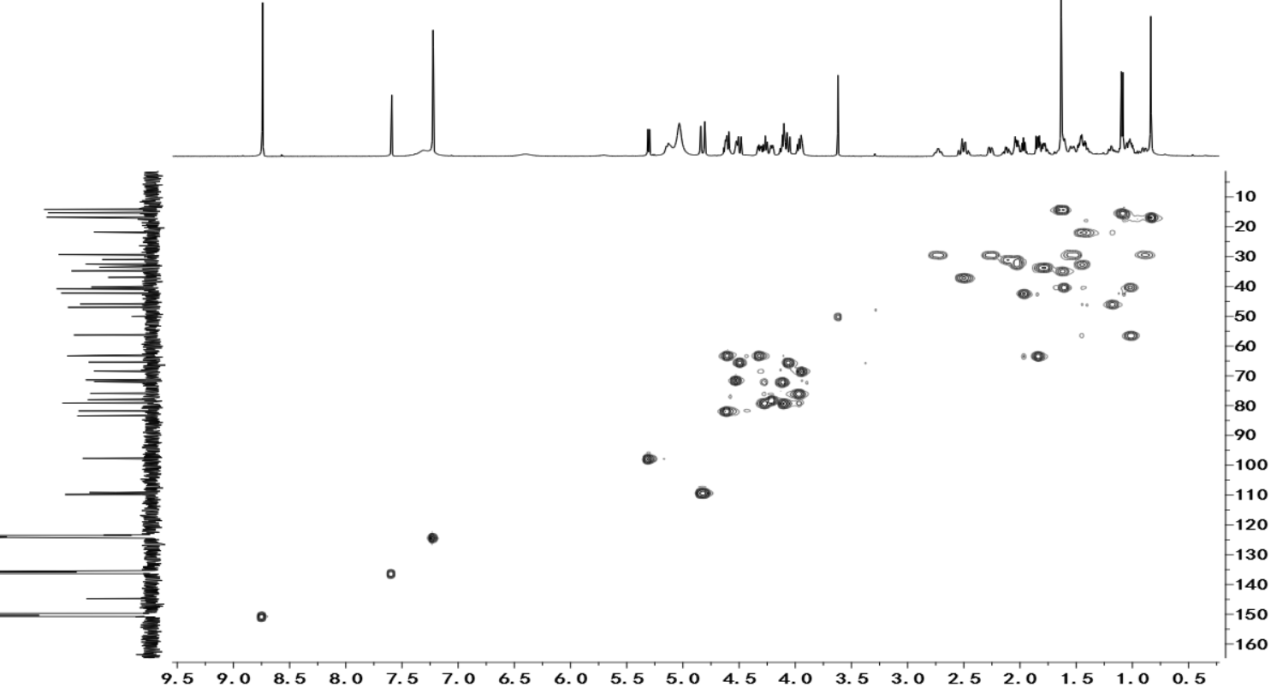


**Figure S35**. HSQC spectrum (500 MHz, C_5_D_5_N) of compound **16**.


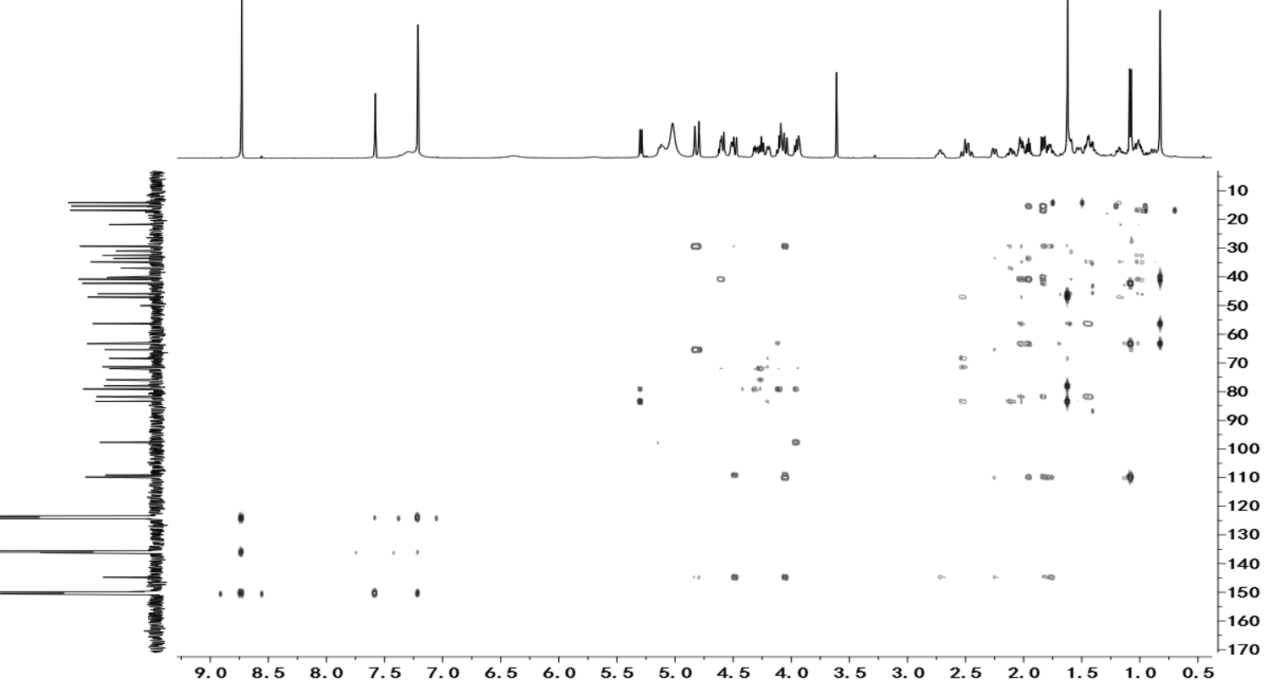


**Figure S36**. HMBC spectrum (500 MHz, C_5_D_5_N) of compound **16**.


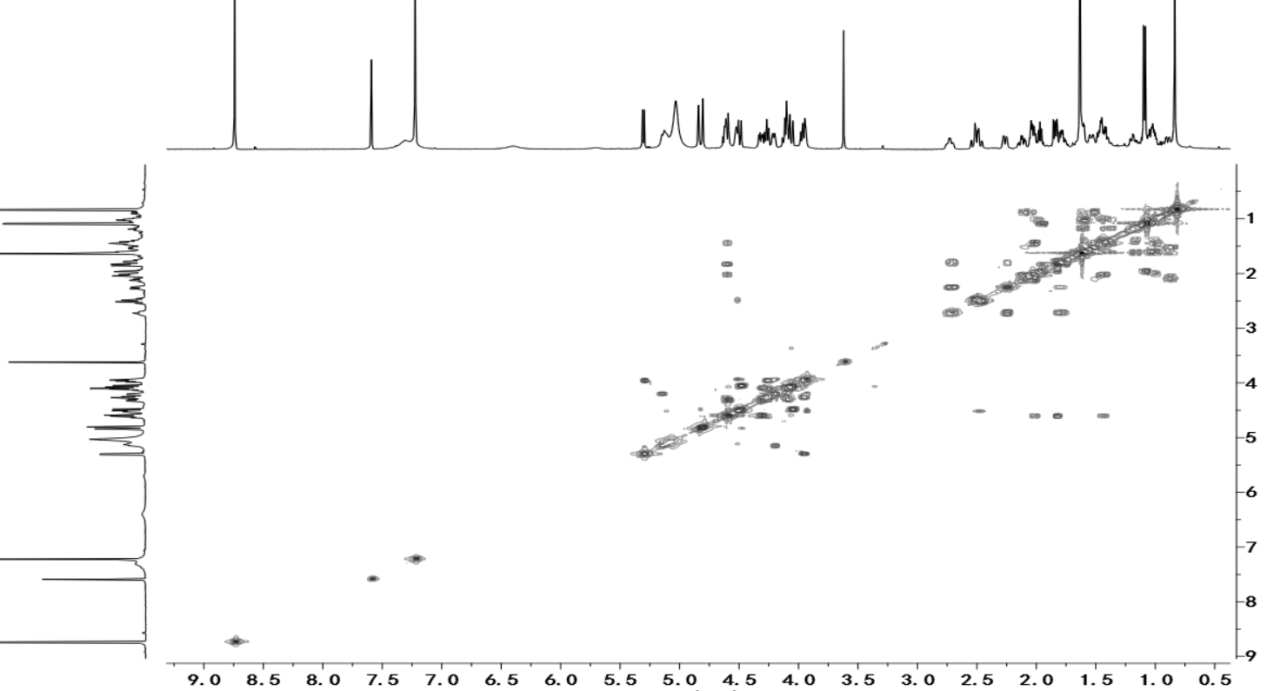


**Figure S37.** ^1^H-^1^H COSY spectrum (500 MHz, C_5_D_5_N) of compound **16**.


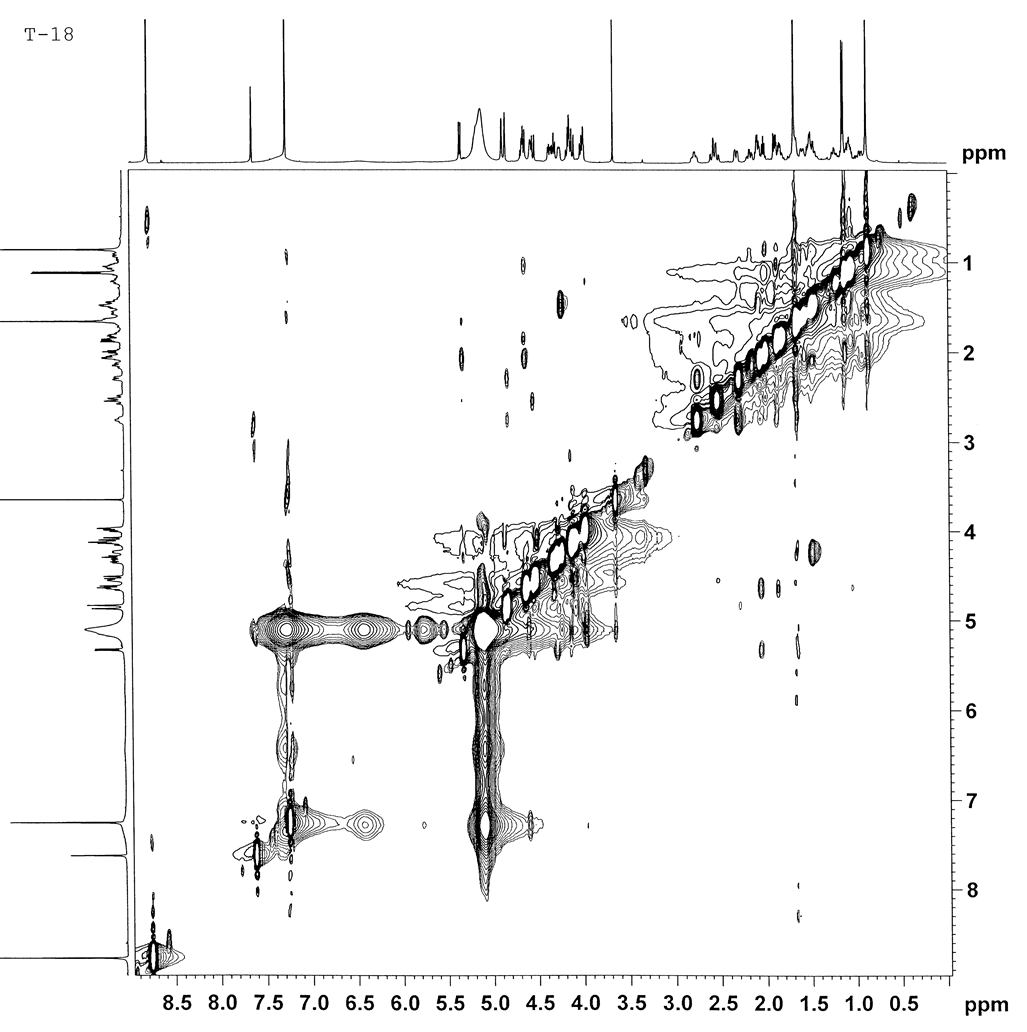


**Figure S38.** NOESY spectrum (500 MHz, C_5_D_5_N) of compound **16.**


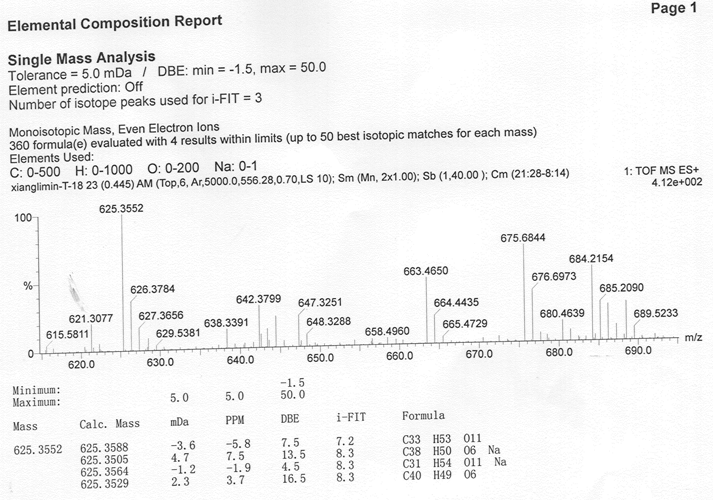


**Figure S39.** HRESIMS spectrum of compound **16.**

**Figure S40.** IR (KBr disc) spectrum of compound **16.**

**
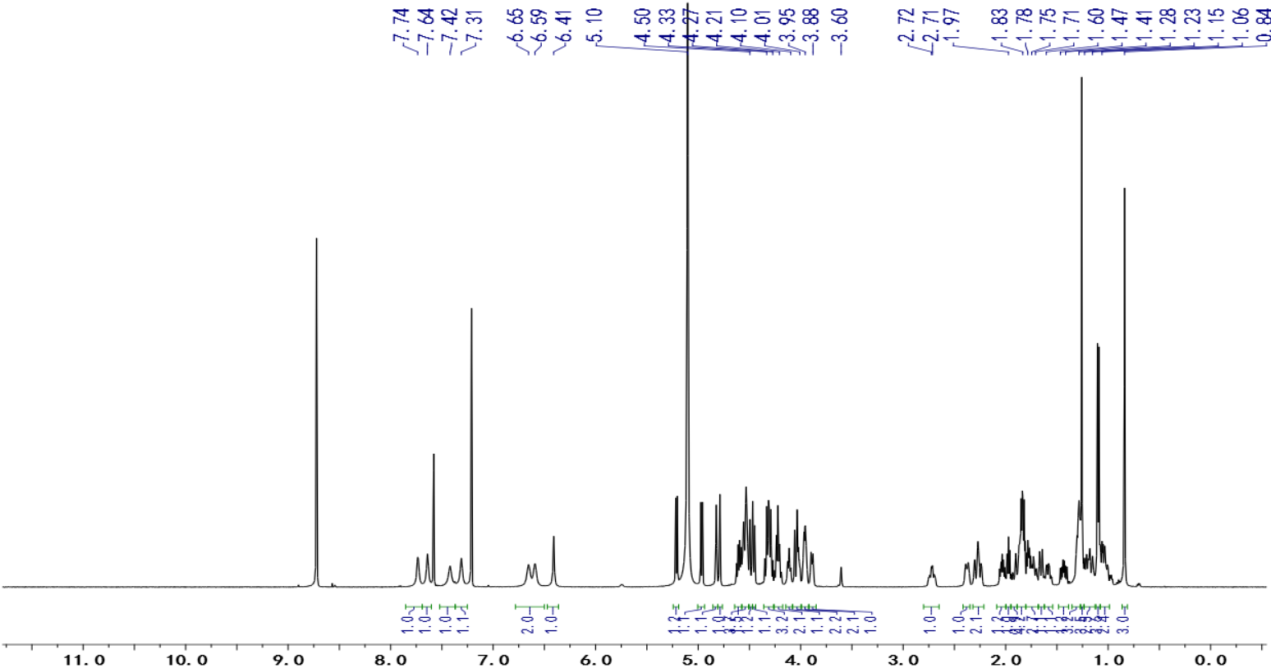
**

**Figure S41.** ^1^H-NMR spectrum (500 MHz, C_5_D_5_N) of compound **17**

**
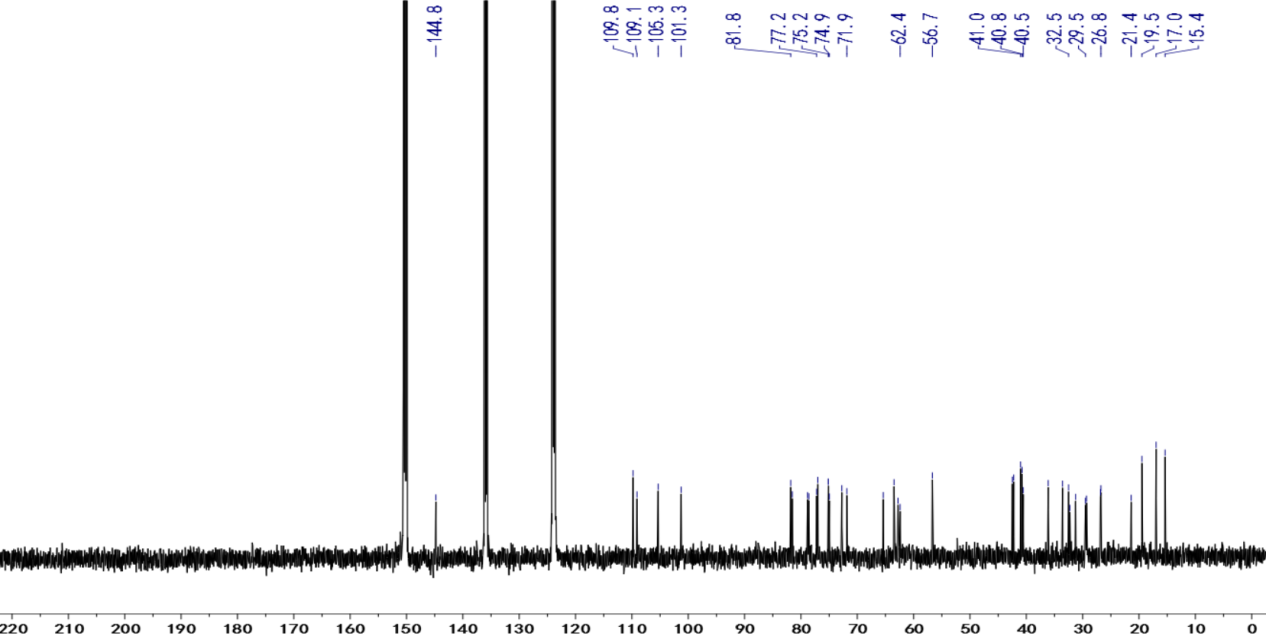
**

**Figure S42.** ^13^C-NMR spectrum (126 MHz, C_5_D_5_N) of compound **17**.


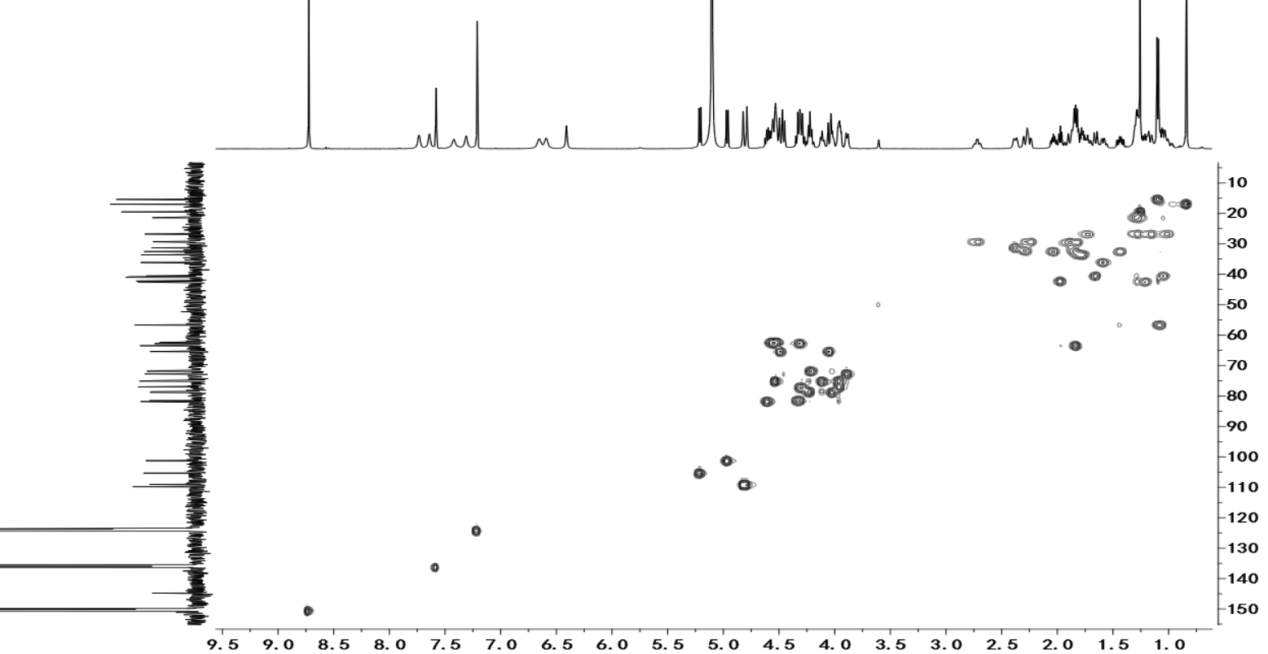


**Figure S43.** HSQC spectrum (500 MHz, C_5_D_5_N) of compound **17**.


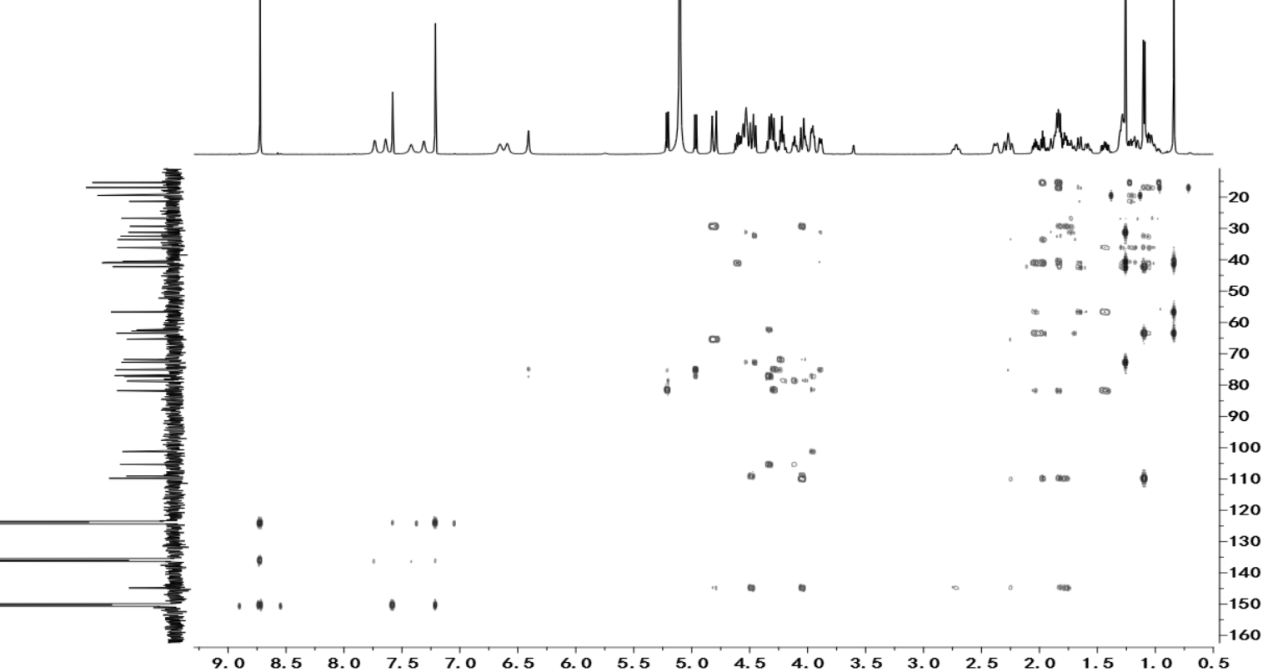


**Figure S44.** HMBC spectrum (500 MHz, C_5_D_5_N) of compound **17**.


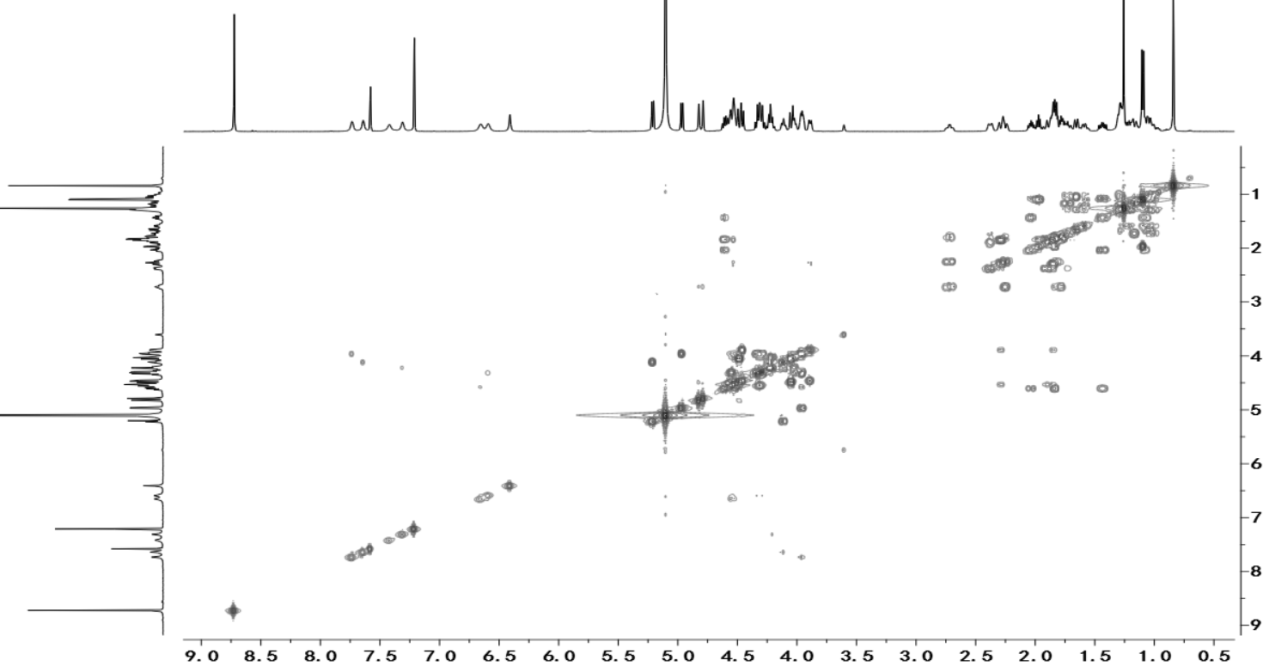


**Figure S45.** ^1^H-^1^H COSY spectrum (500 MHz, C_5_D_5_N) of compound **17**.


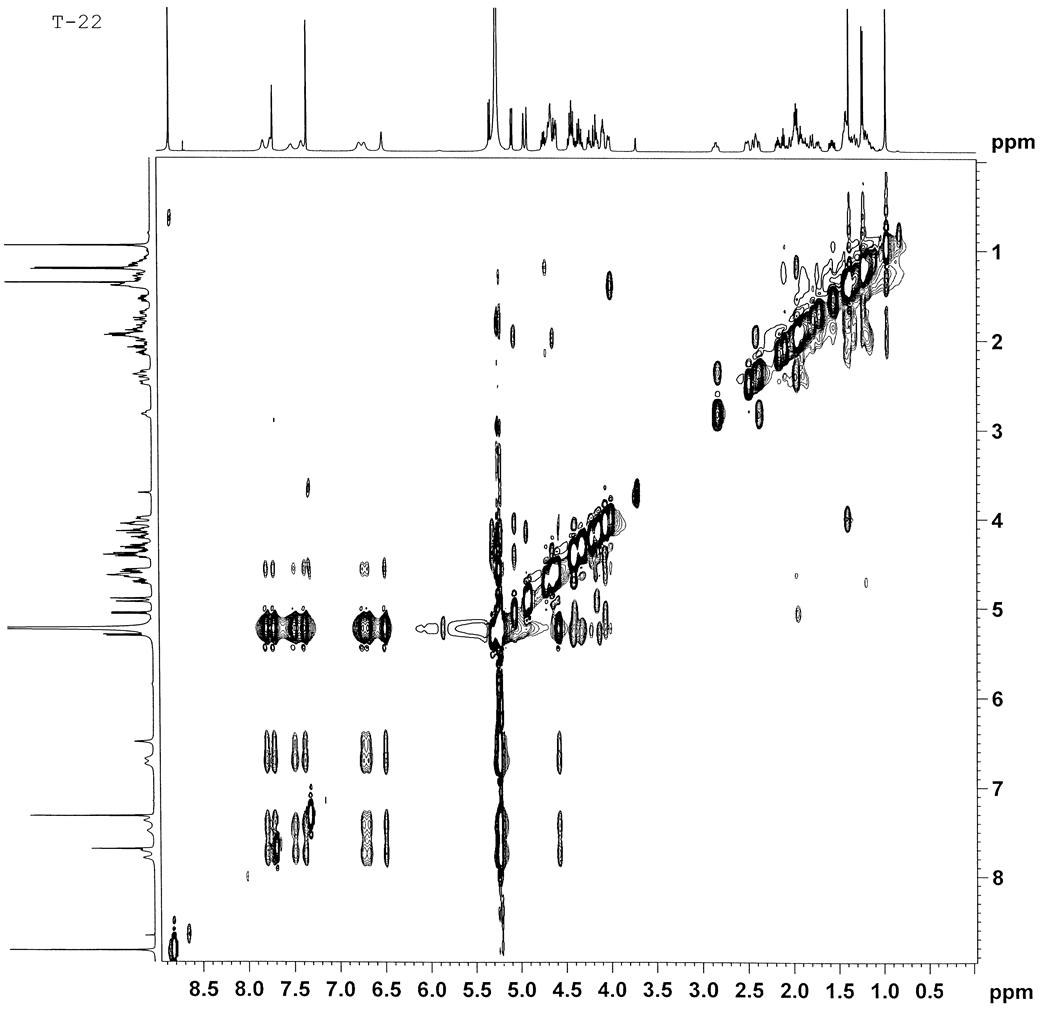


**Figure S46.** NOESY spectrum (500 MHz, C_5_D_5_N) of compound **17**.


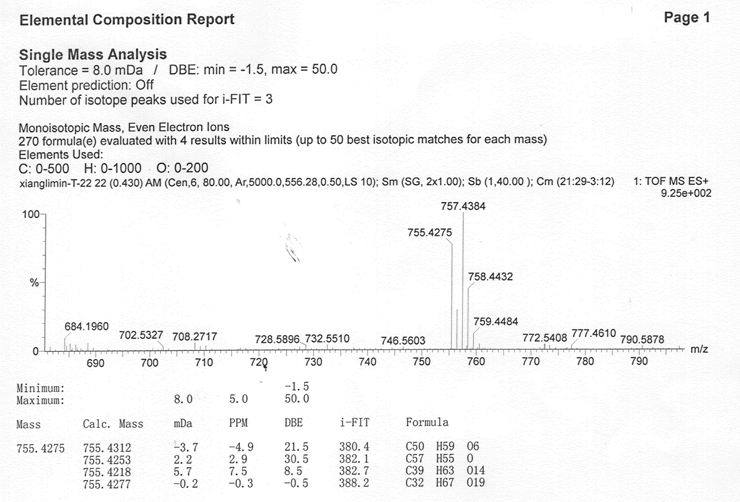


**Figure S47.** HRHRESIMS spectrum of compound **17**.

**Figure S48.** IR (KBr disc) spectrum of compound **17.**

**
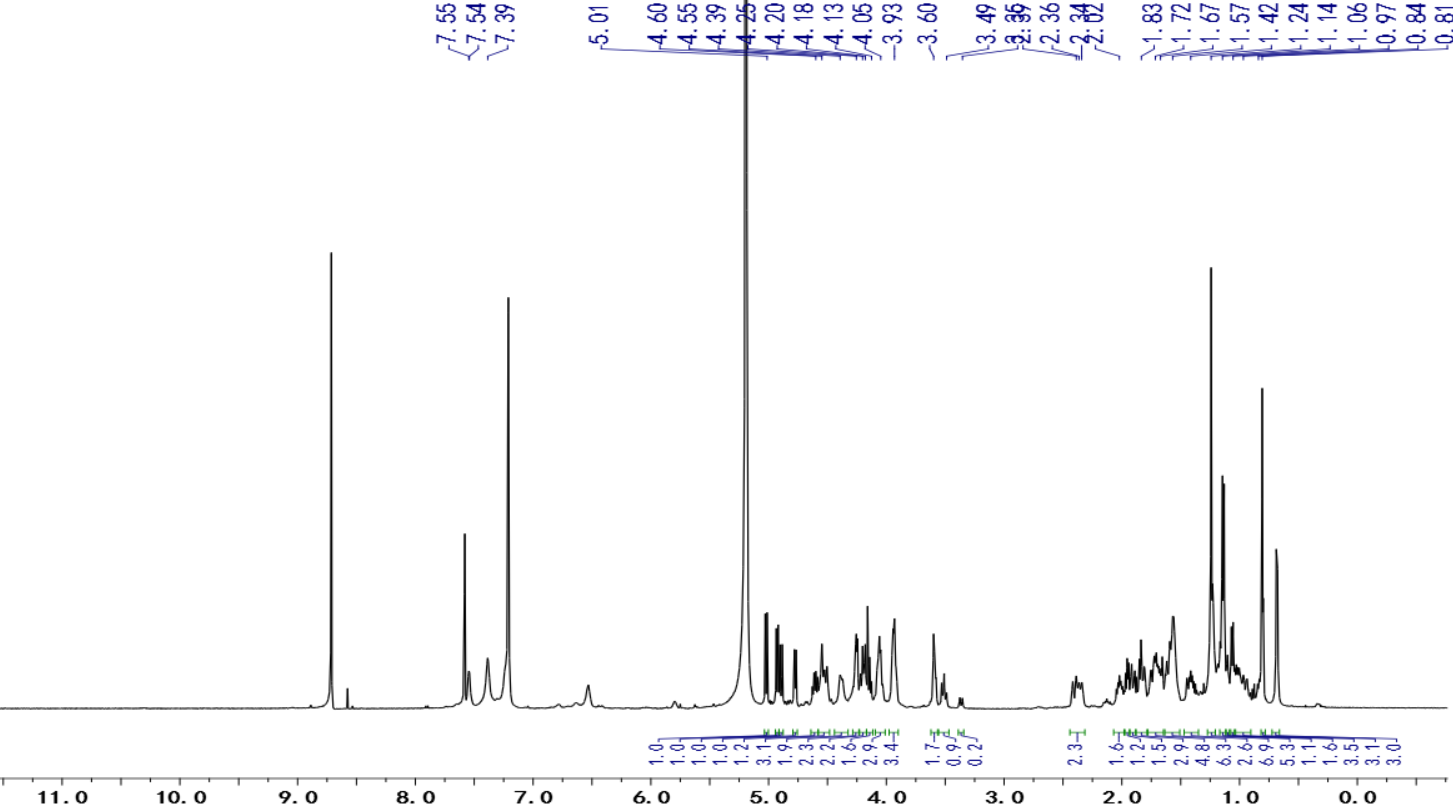
**

**Figure S49.** ^1^H-NMR spectrum (500 MHz, C_5_D_5_N) of compound **18**.

**
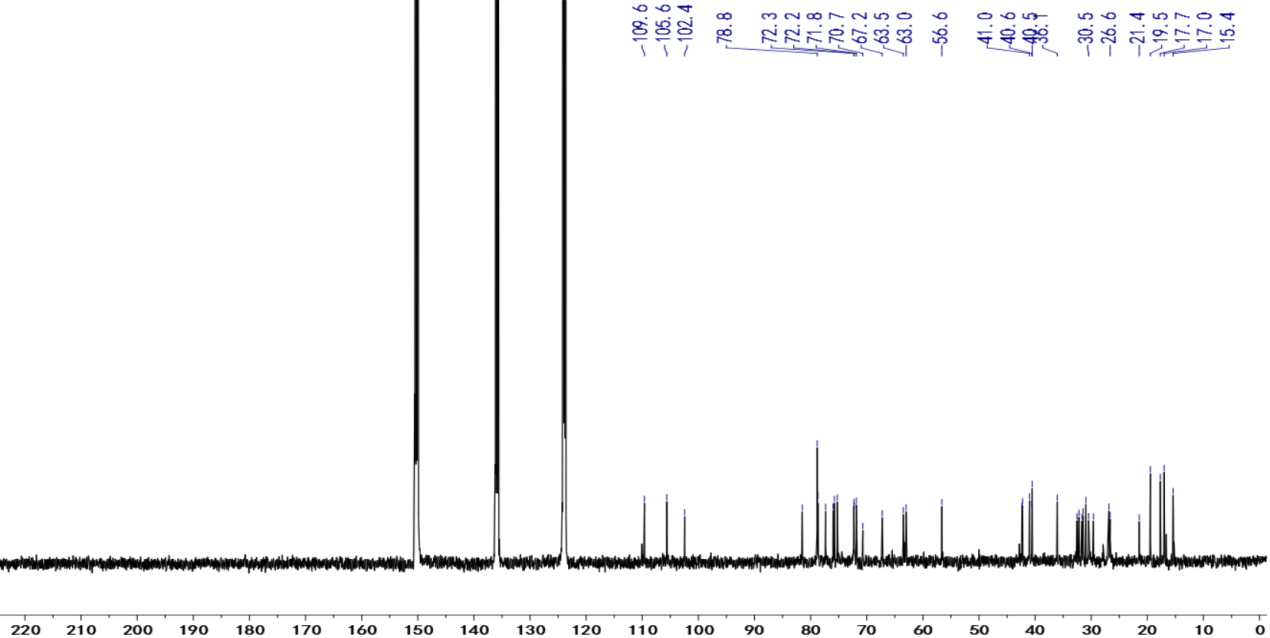
**

**Figure S50.** ^13^C-NMR spectrum (126 MHz, C_5_D_5_N) of compound **18**.


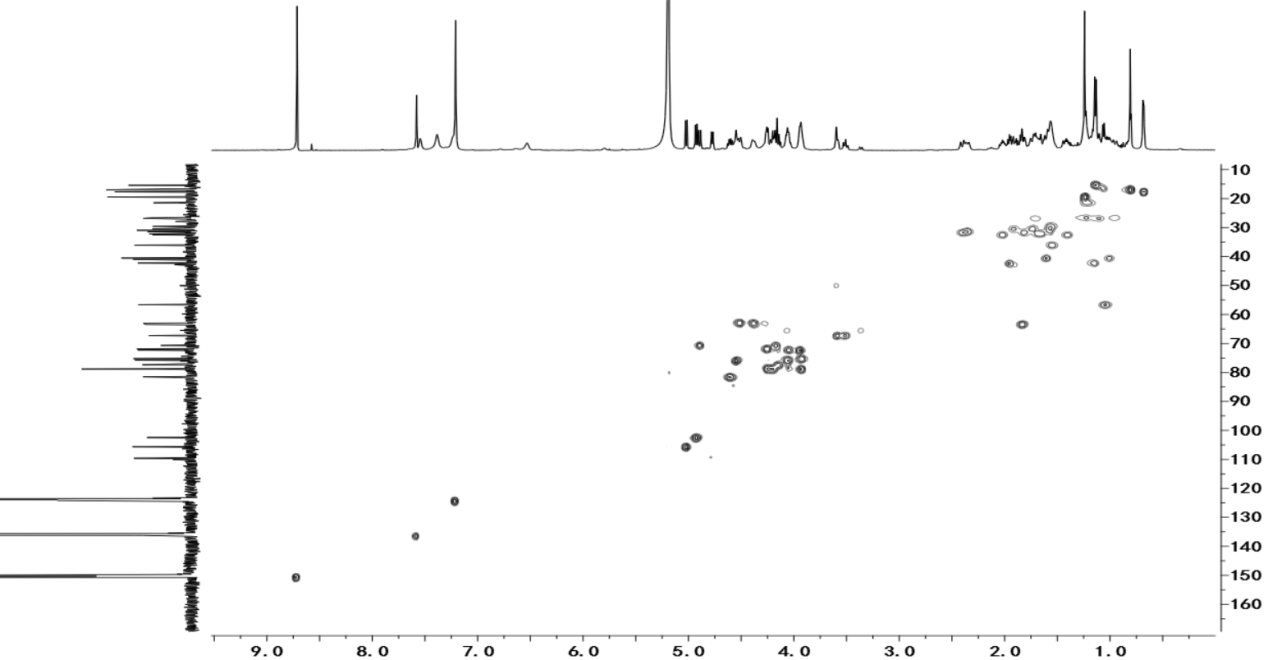


**Figure S51**. HSQC spectrum (500 MHz, C_5_D_5_N) of compound **18**.


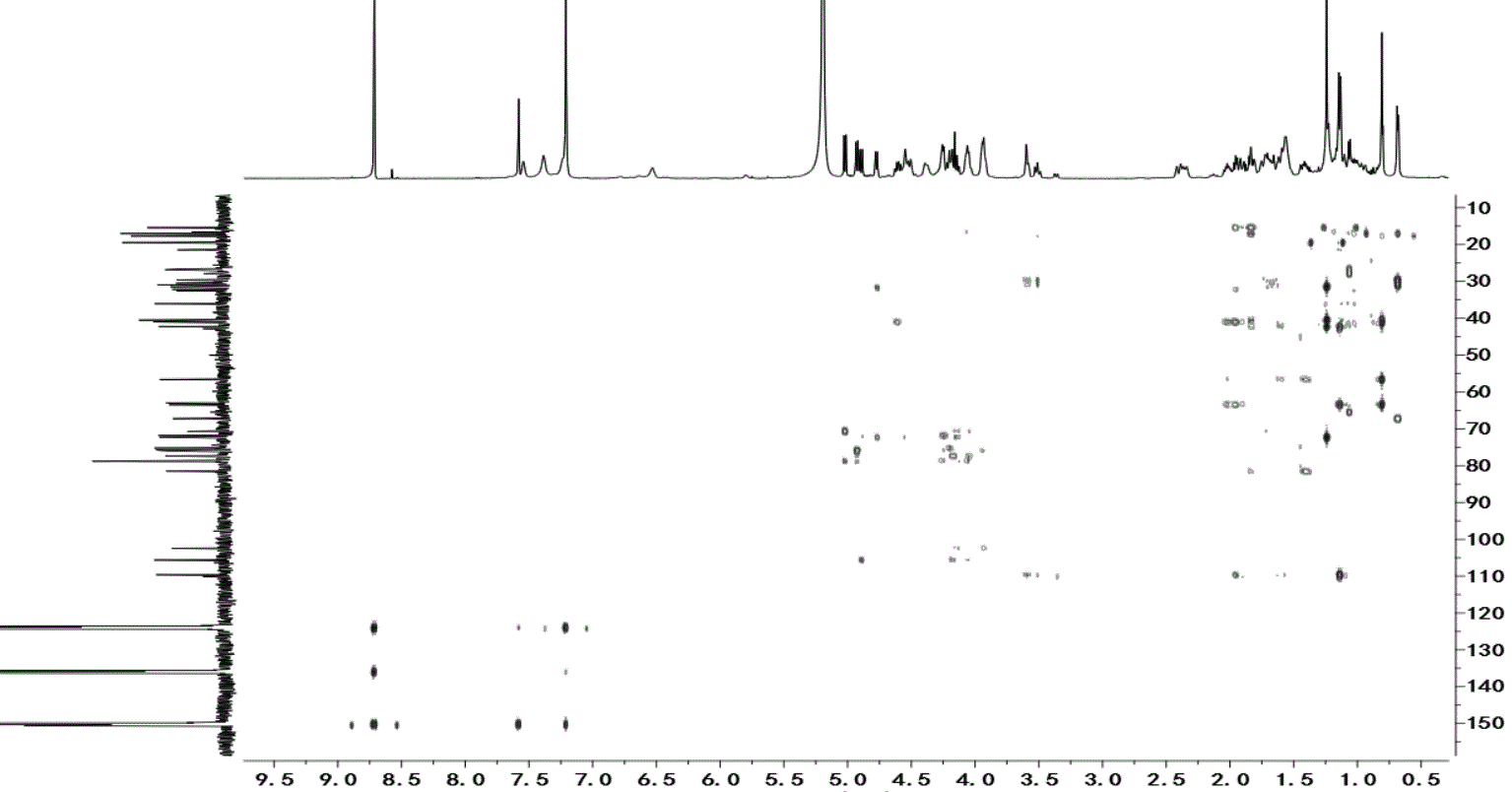


**Figure S52.** HMBC spectrum (500 MHz, C_5_D_5_N) of compound **18**.


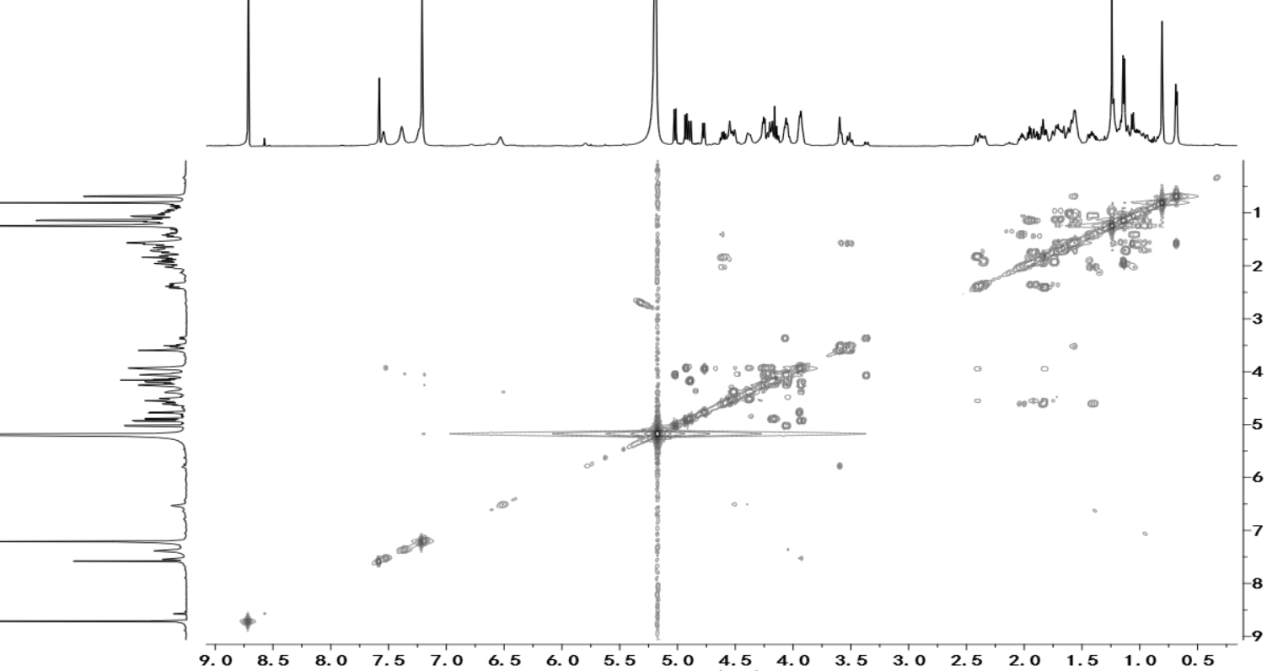


**Figure S53.** ^1^H-^1^H COSY spectrum (500 MHz, C_5_D_5_N) of compound **18**.


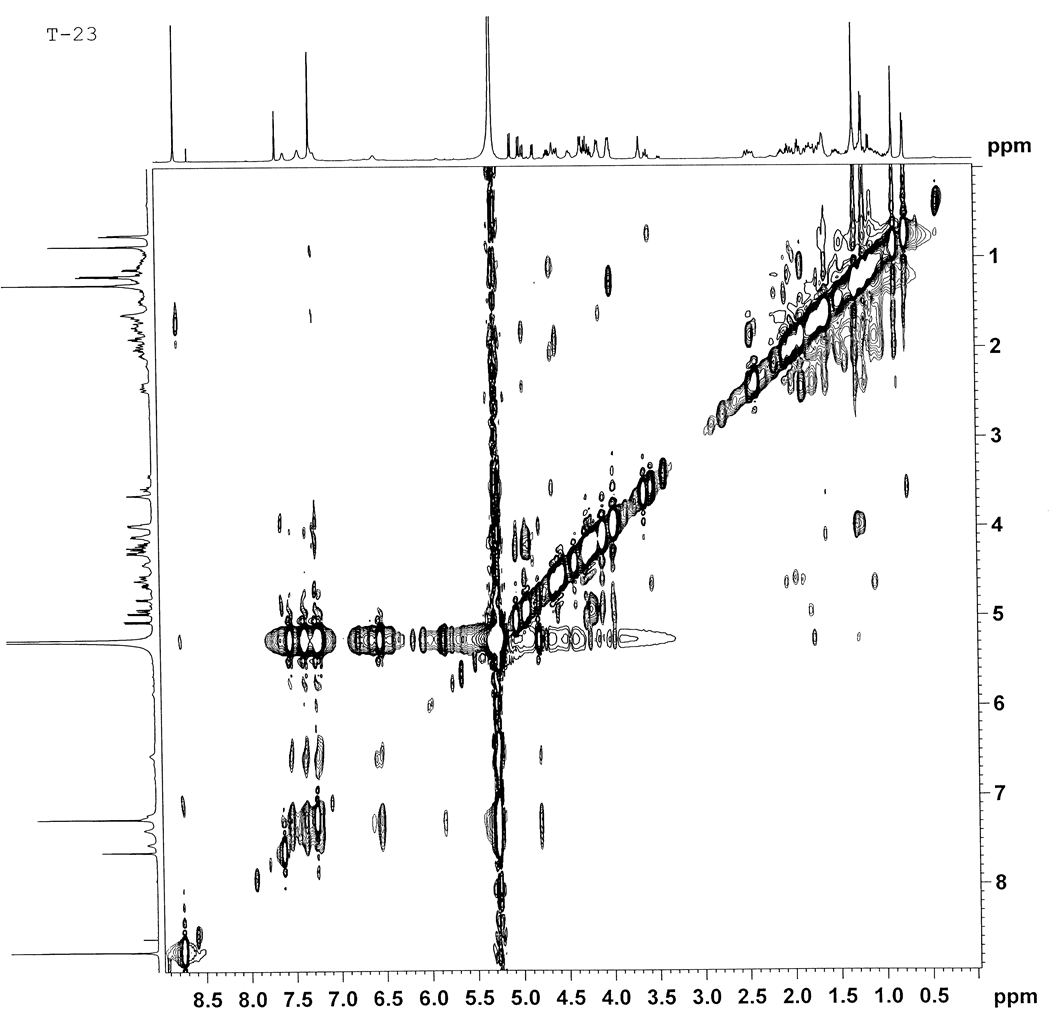


**Figure S54.** NOESY spectrum (500 MHz, C_5_D_5_N) of compound **18**.


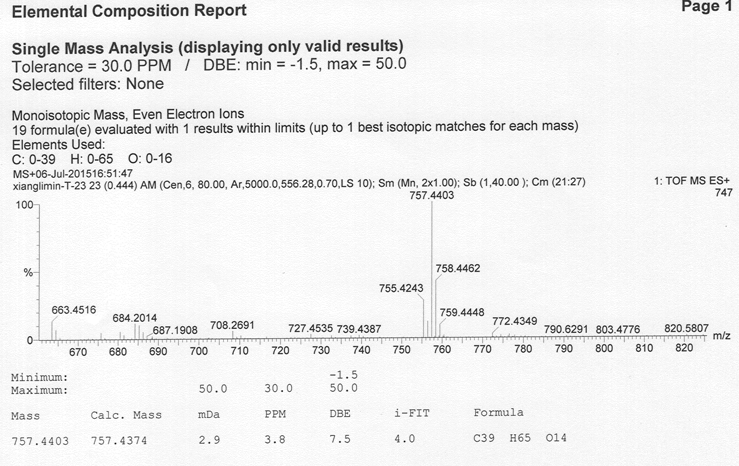


**Figure S55.** HRESIMS spectrum of compound **18**.

**Figure S56.** IR (KBr disc) spectrum of compound **18.**


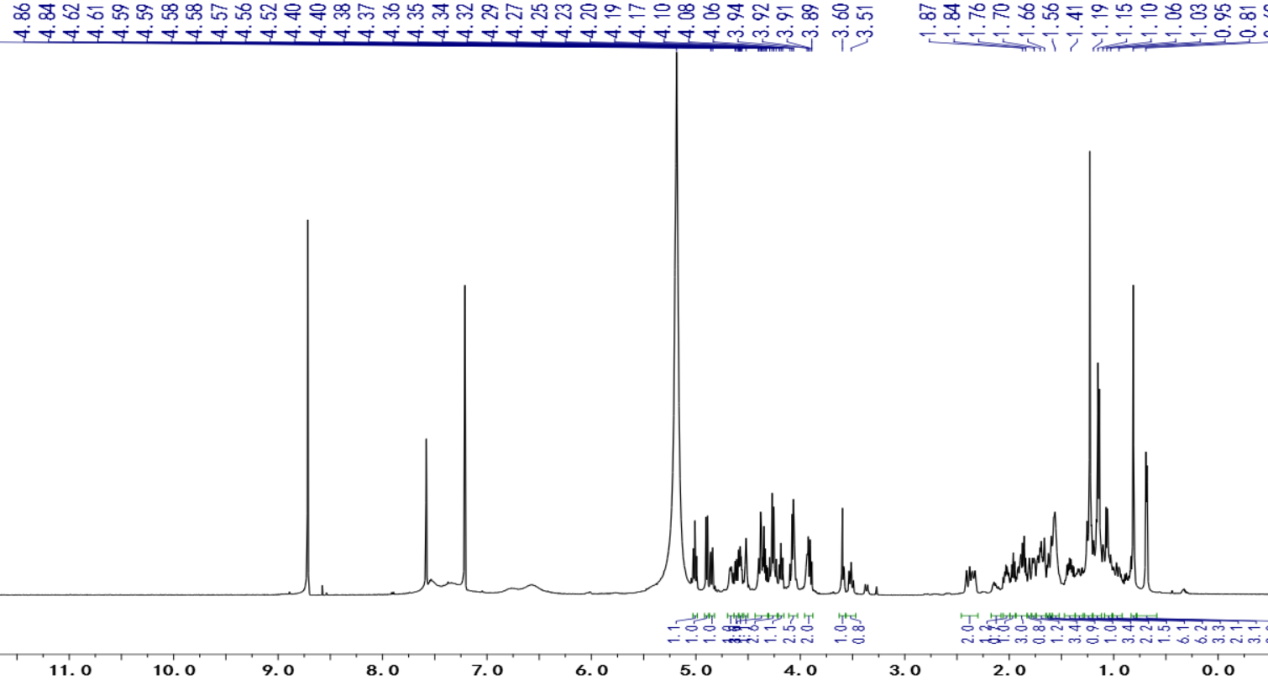


**Figure S57.** ^1^H-NMR spectrum (500 MHz, C_5_D_5_N) of compound **19**.


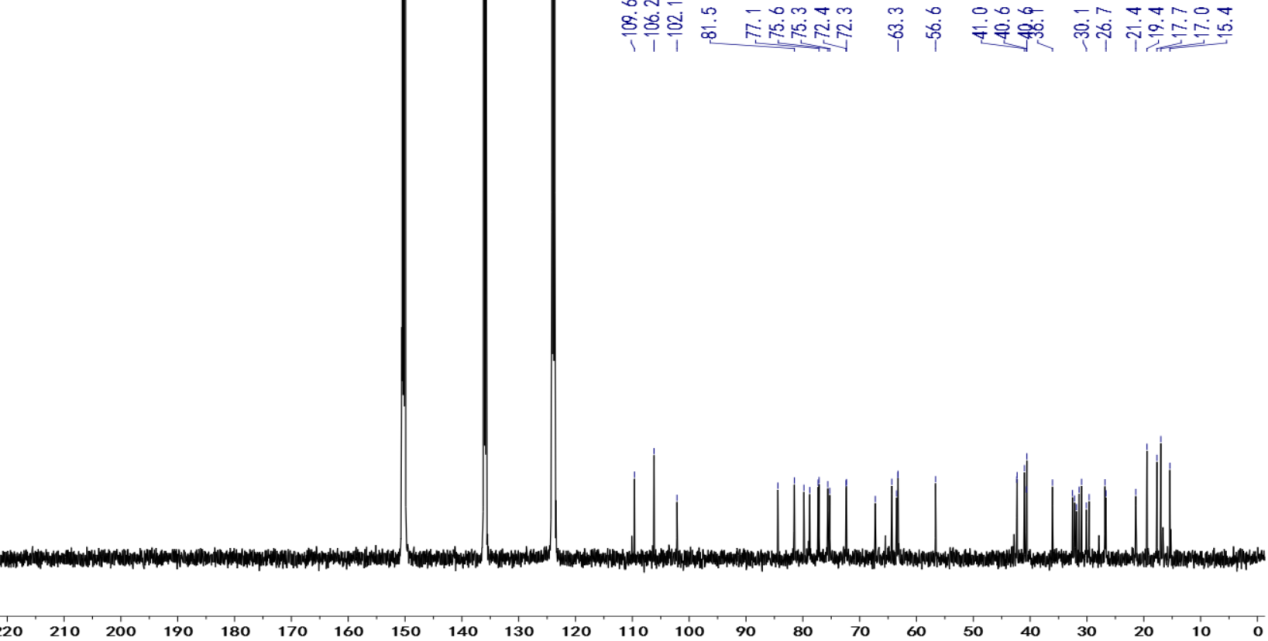


**Figure S58.** ^13^C-NMR spectrum (126 MHz, C_5_D_5_N) of compound **19**.


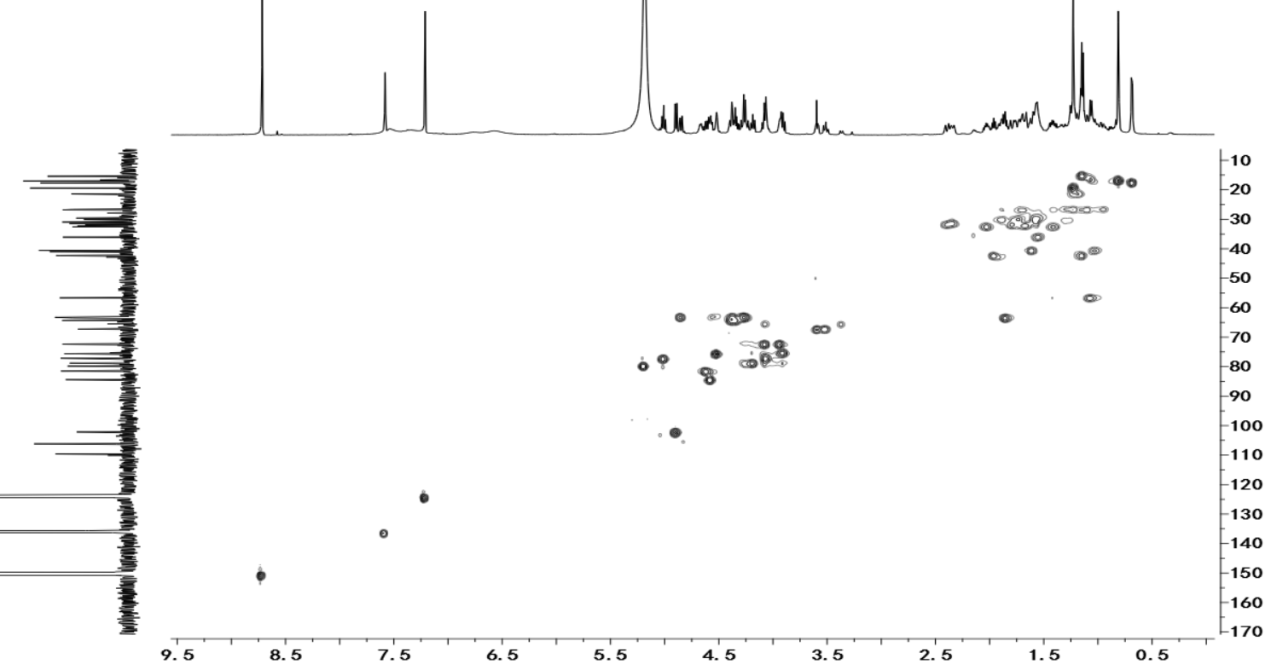


**Figure S59**. HSQC spectrum (500 MHz, C_5_D_5_N) of compound **19**.


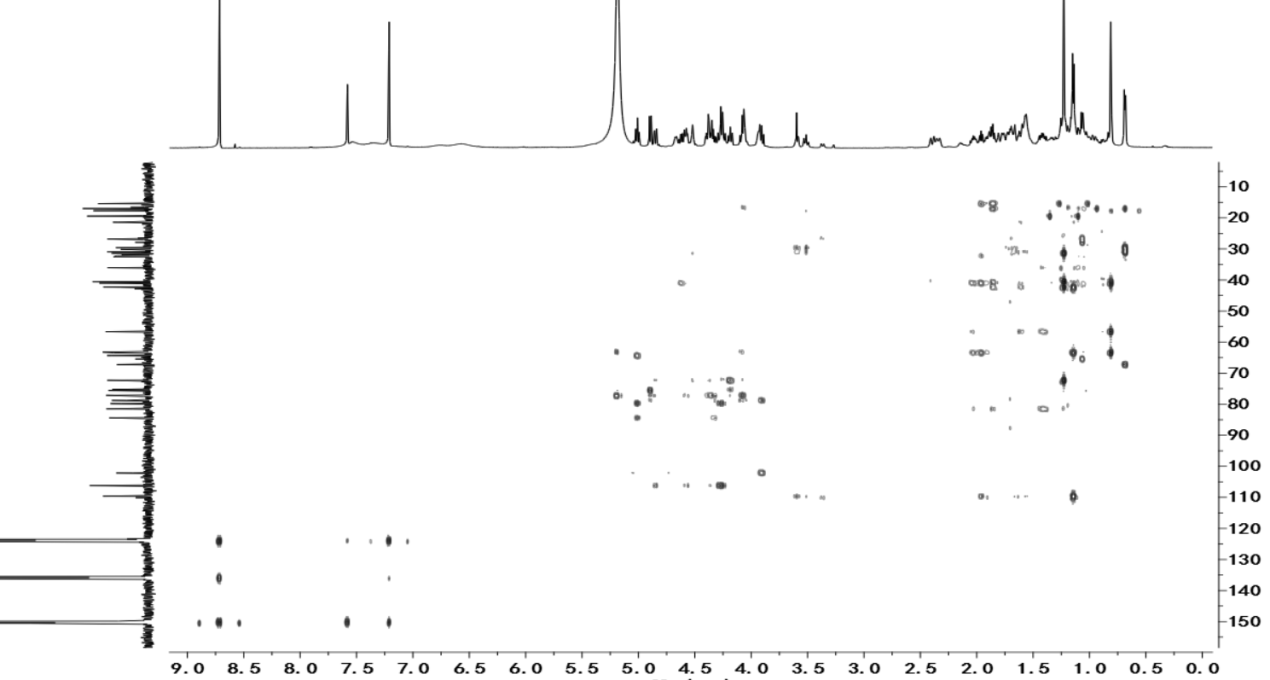


**Figure S60**. HMBC spectrum (500 MHz, C_5_D_5_N) of compound **19**.


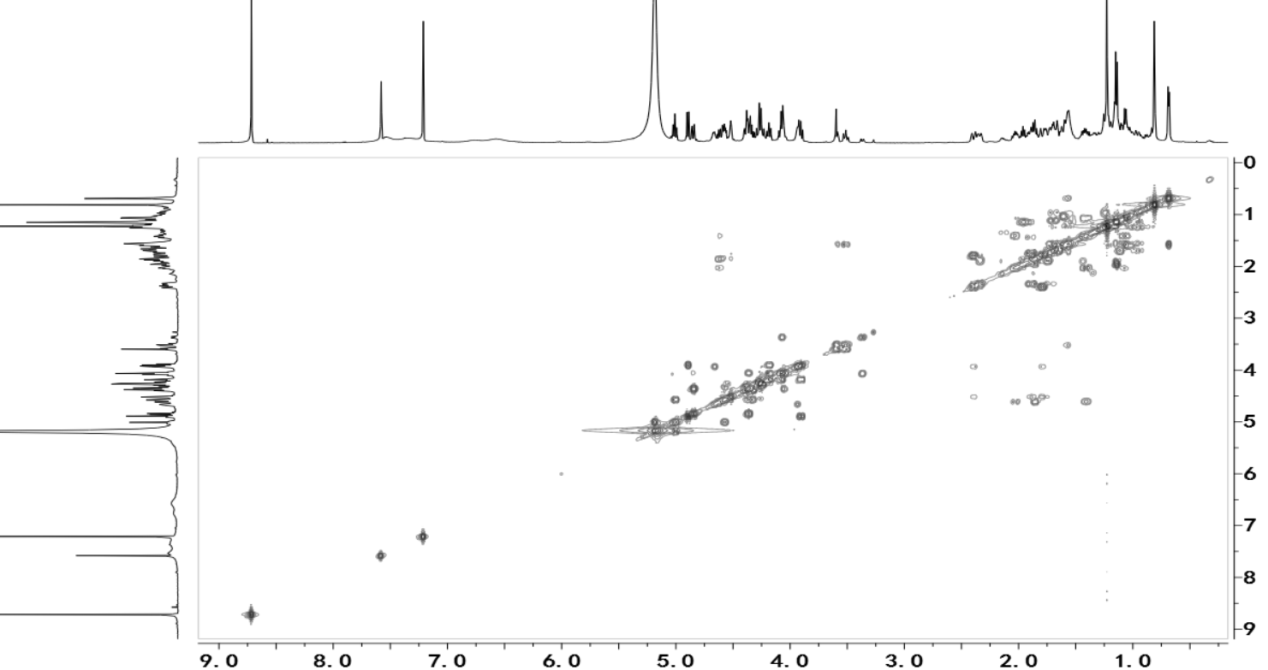


**Figure S61.** ^1^H-^1^H COSY spectrum (500 MHz, C_5_D_5_N) of compound **19**.


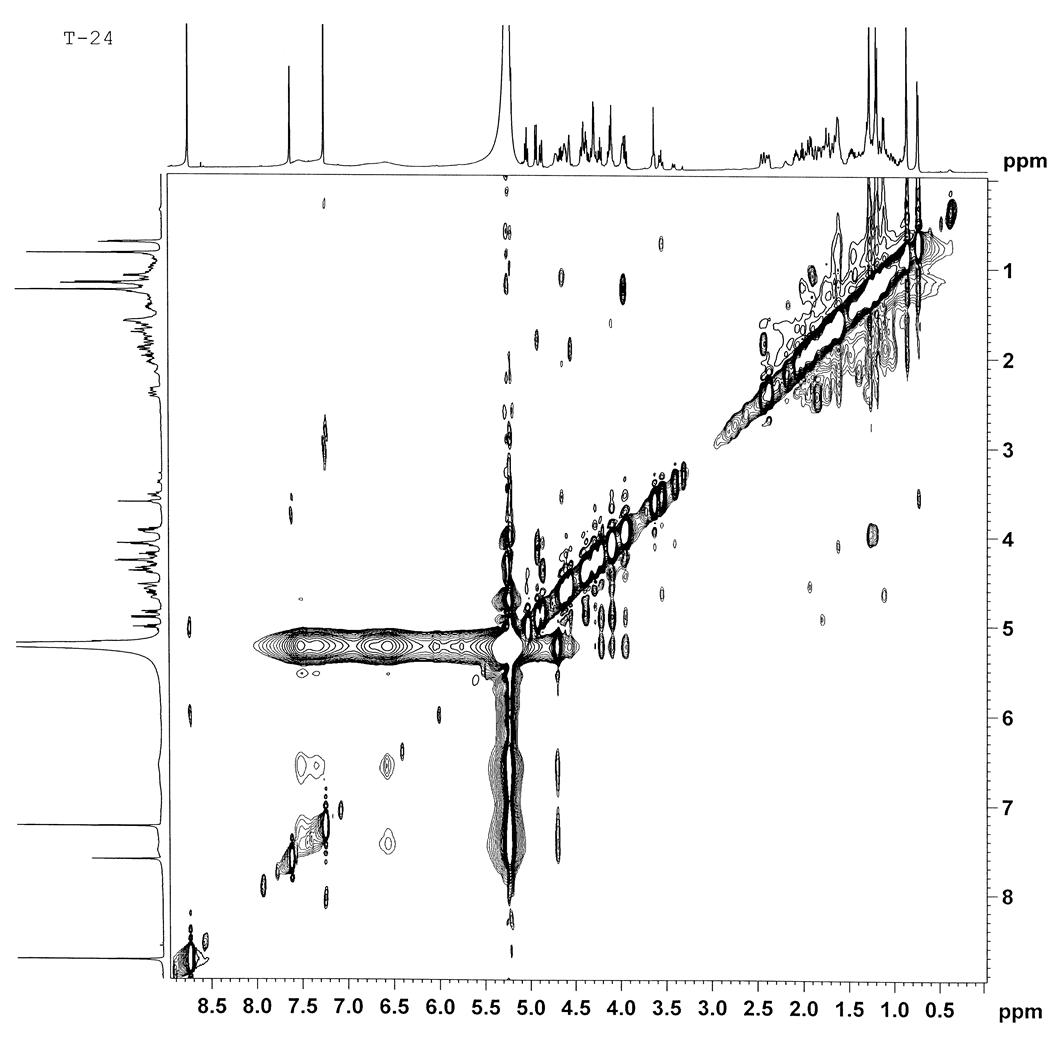


**Figure S62.** NOESY spectrum (500 MHz, C_5_D_5_N) of compound **19**.


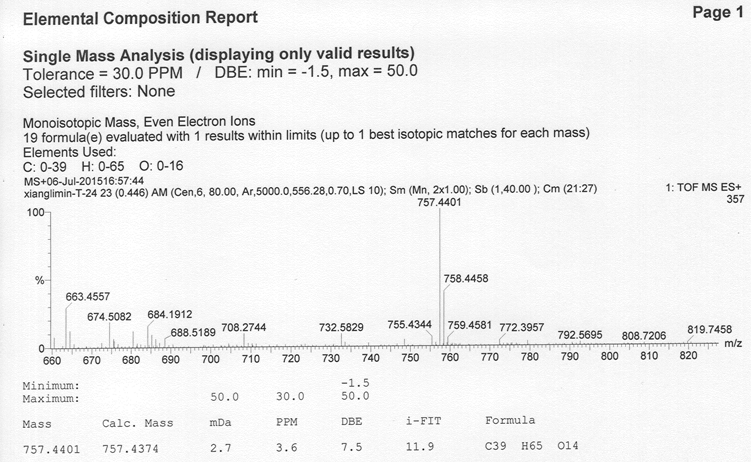


**Figure S63.** HRESIMS spectrum of compound **19**.

**Figure S64.** IR (KBr disc) spectrum of compound **19.**

**
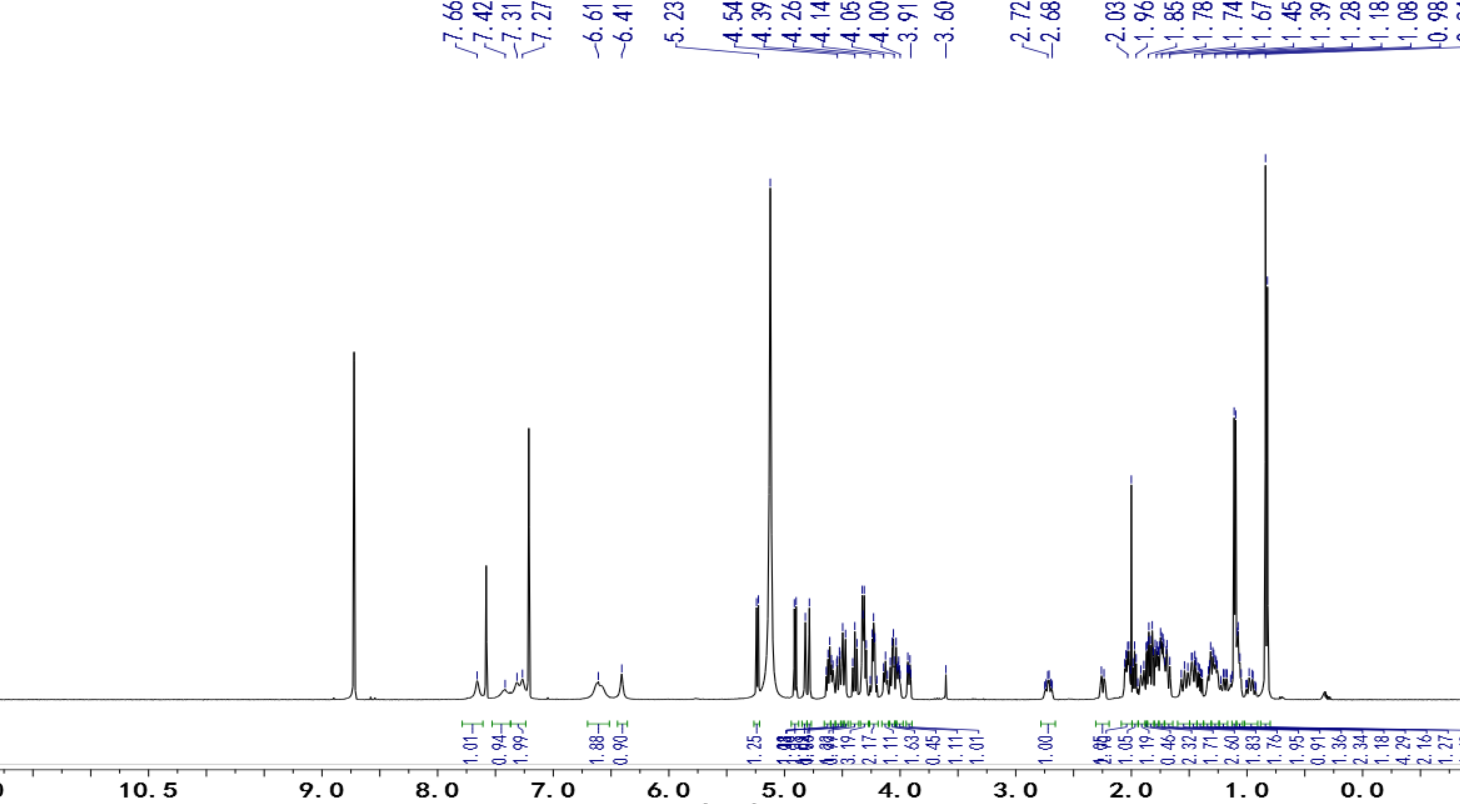
**

**Figure S65.** ^1^H-NMR spectrum (500 MHz, C_5_D_5_N) of compound **20**.


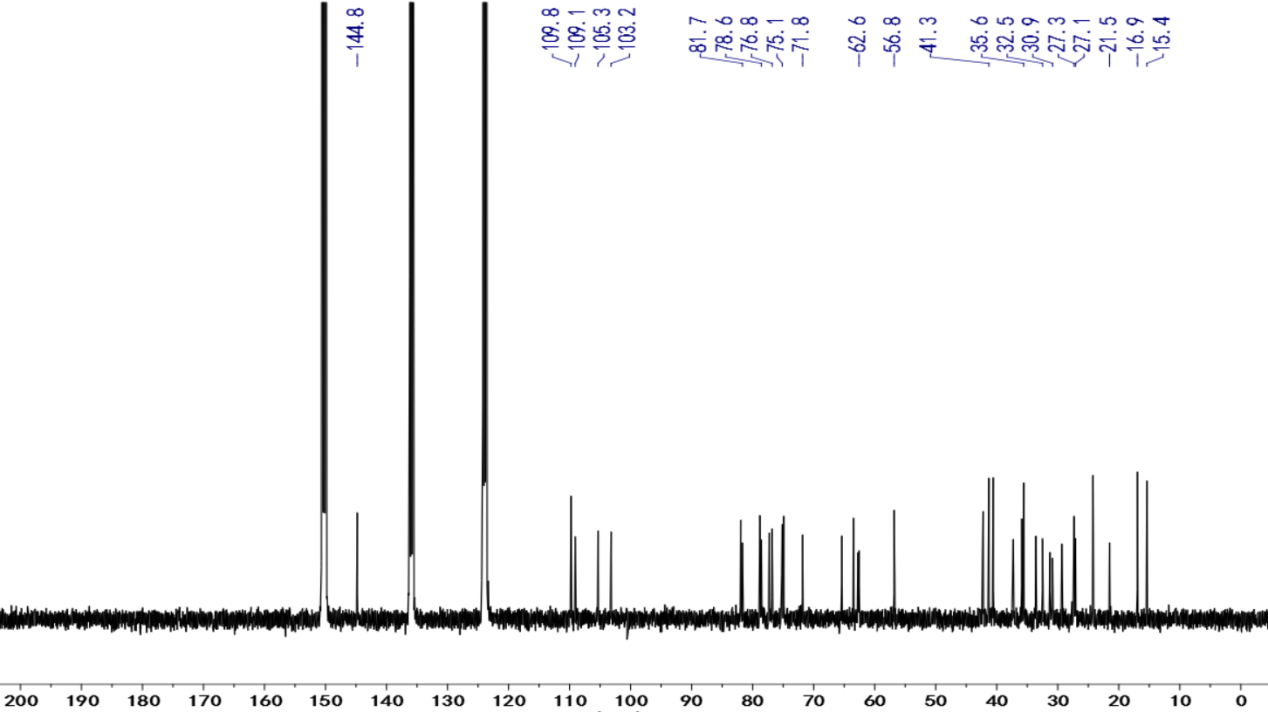


**Figure S66.** ^13^C-NMR spectrum (126 MHz, C_5_D_5_N) of compound **20**.


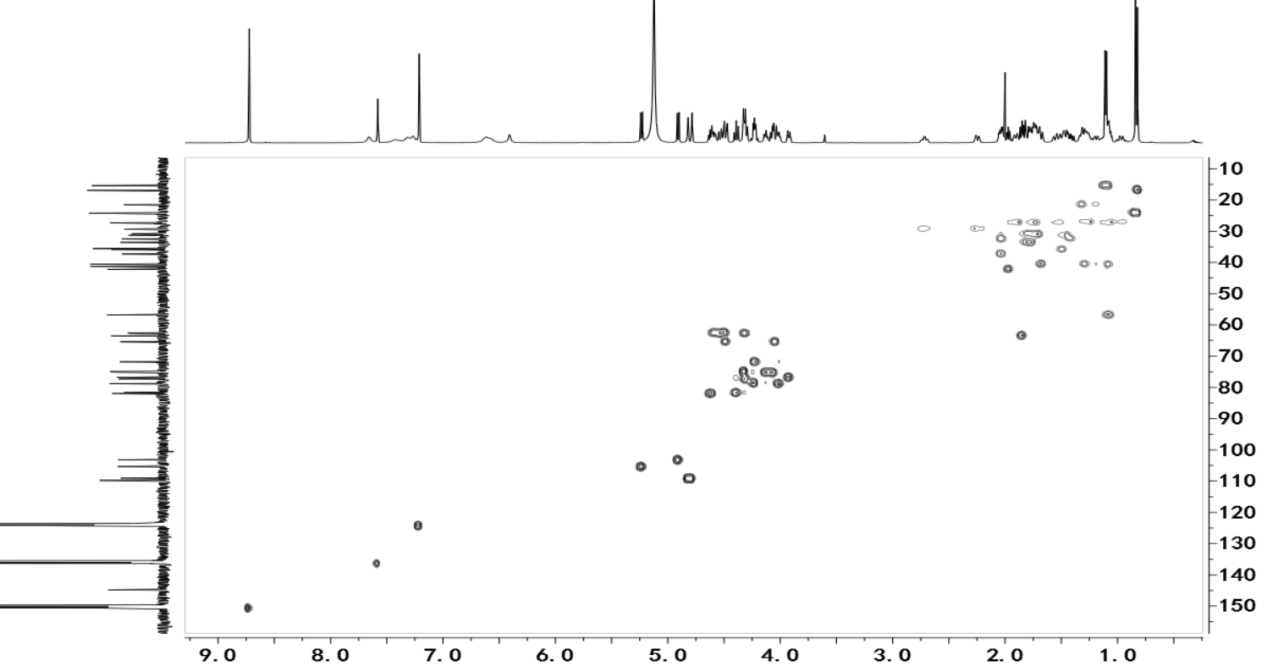


**Figure S67**. HSQC spectrum (500 MHz, C_5_D_5_N) of compound **20**.


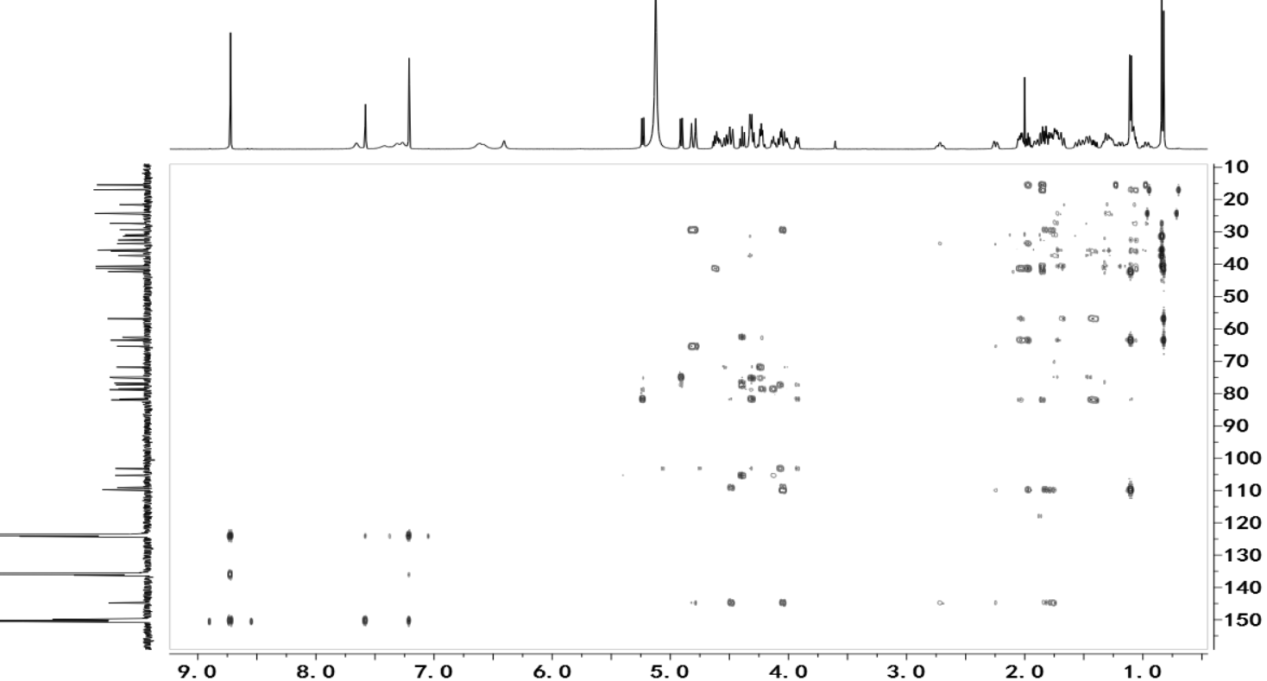


**Figure S68**. HMBC spectrum (500 MHz, C_5_D_5_N) of compound **20**.


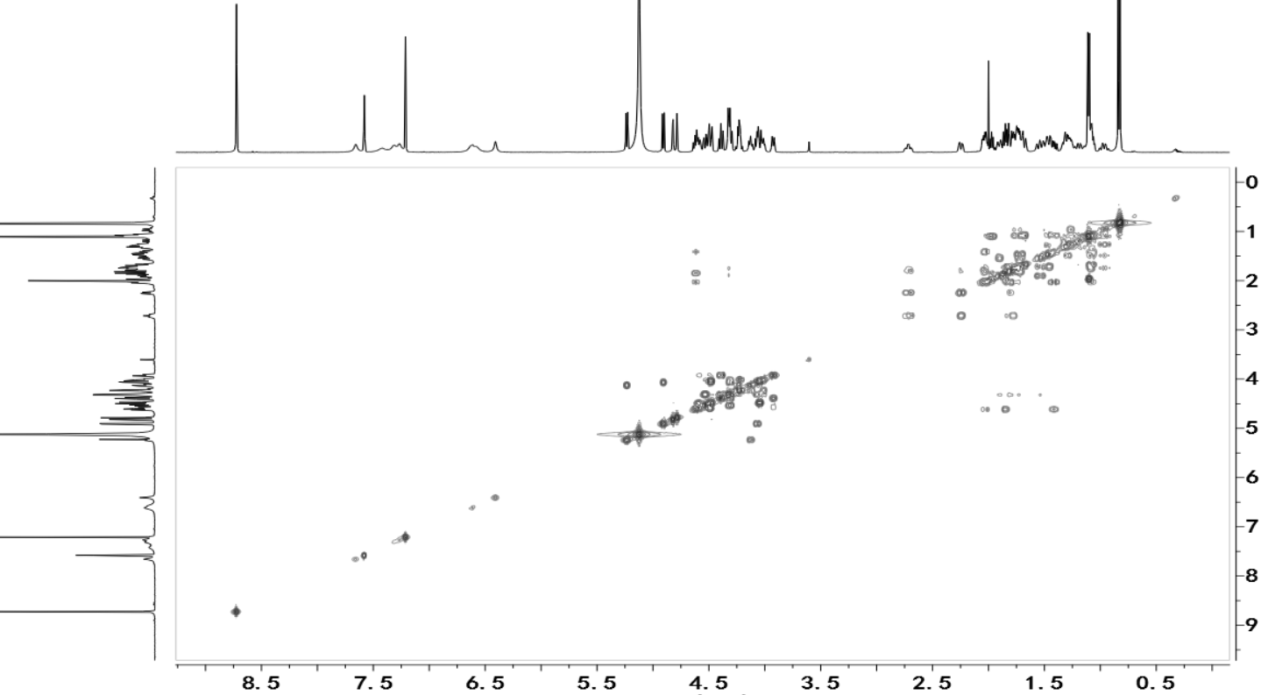


**Figure S69.** ^1^H-^1^H COSY spectrum (500 MHz, C_5_D_5_N) of compound **20**.


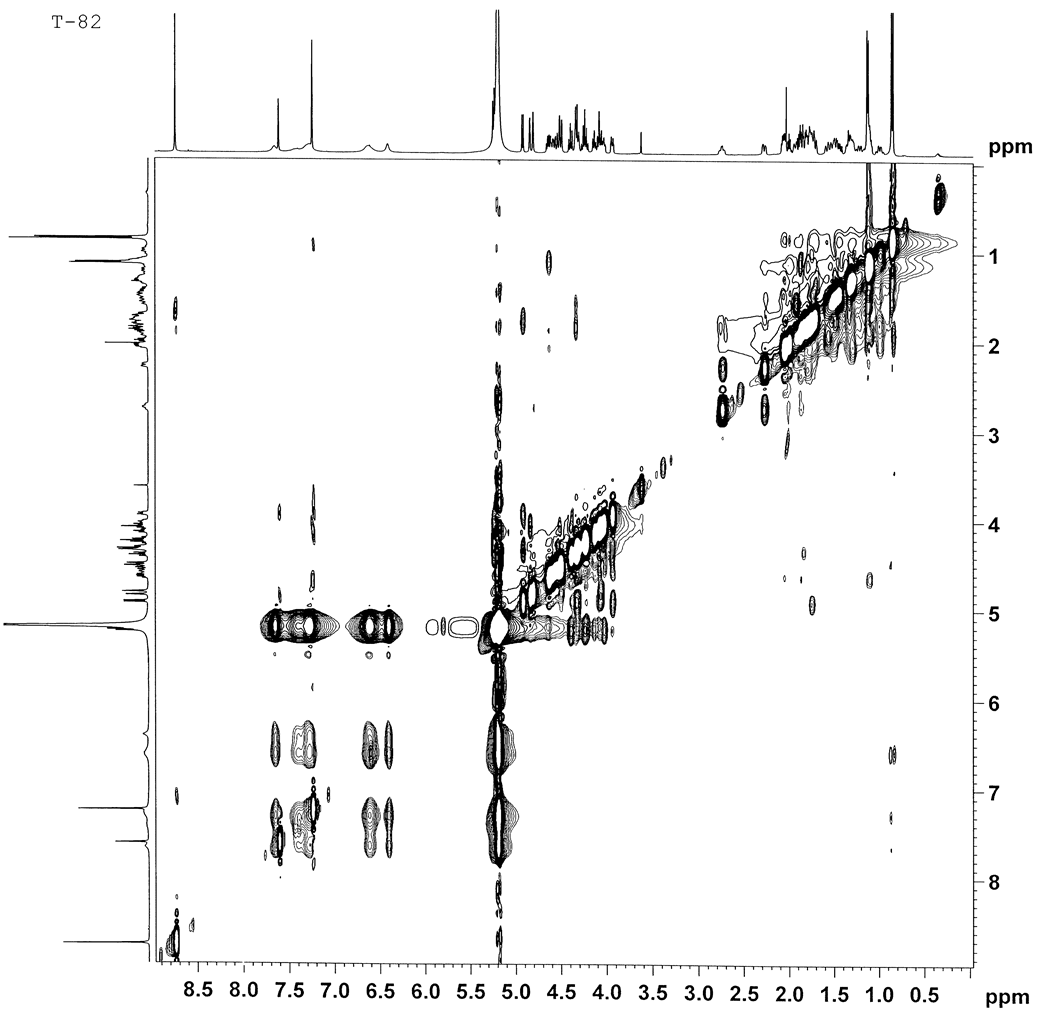


**Figure S70.** NOESY spectrum (500 MHz, C_5_D_5_N) of compound **20.**


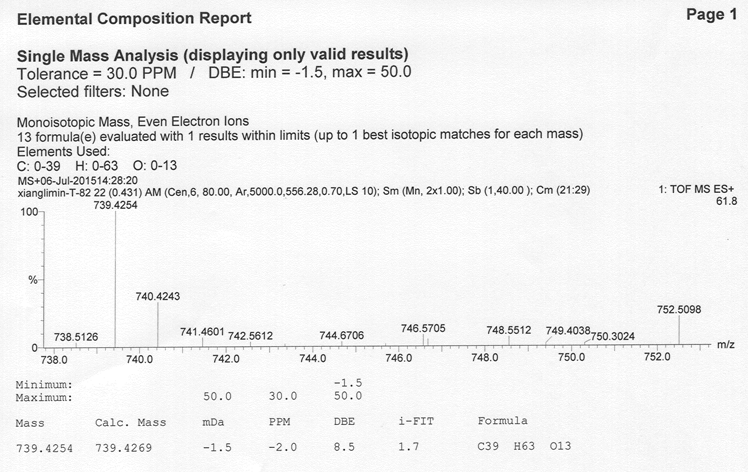


**Figure S71.** HRESIMS spectrum of compound **20.**

**Figure S72.** IR (KBr disc) spectrum of compound **20.**
